# A coffee berry borer (*Hypothenemus hampei*) genome assembly reveals a reduced chemosensory receptor gene repertoire and male-specific genome sequences

**DOI:** 10.1038/s41598-021-84068-1

**Published:** 2021-03-01

**Authors:** Lucio Navarro-Escalante, Erick M. Hernandez-Hernandez, Jonathan Nuñez, Flor E. Acevedo, Alejandro Berrio, Luis M. Constantino, Beatriz E. Padilla-Hurtado, Diana Molina, Carmenza Gongora, Ricardo Acuña, Jeff Stuart, Pablo Benavides

**Affiliations:** 1 National Coffee Research Center-CENICAFE, Manizales, Colombia; 2 Estadual Paulista, UNESP-Univ, São José Do Rio Preto, SP Brazil; 3 Manaaki Whenua–Landcare Research, PO Box 69040, Lincoln, 7640 New Zealand; 4 Department of Entomology, Pennsylvania State University, University Park, PA USA; 5 Department of Biology, Duke University, Durham, USA; 6 Instituto de Investigación en Microbiología Y Biotecnología Agroindustrial, Universidad Católica de Manizales, Manizales, Colombia; 7 Department of Entomology, Purdue University, West Lafayette, USA

**Keywords:** Ecology, Genetics, Molecular biology, Entomology

## Abstract

Coffee berry borer—CBB (*Hypothenemus hampei*) is a globally important economic pest of coffee (*Coffea* spp.). Despite current insect control methods for managing CBB, development of future control strategies requires a better understanding of its biology and interaction with its host plant. Towards this objective, we performed de novo CBB genome and transcriptome sequencing, improved CBB genome assembly and predicted 18,765 protein-encoding genes. Using genome and transcriptome data, we annotated the genes associated with chemosensation and found a reduced gene repertoire composed by 67 odorant receptors (ORs), 62 gustatory receptors (GRs), 33 ionotropic receptors (IRs) and 29 odorant-binding proteins (OBPs). In silico transcript abundance analysis of these chemosensory genes revealed expression enrichment in CBB adults compared with larva. Detection of differentially expressed chemosensory genes between males and females is likely associated with differences in host-finding behavior between sexes. Additionally, we discovered male-specific genome content and identified candidate male-specific expressed genes on these scaffolds, suggesting that a Y-like chromosome may be involved in the CBB’s functional haplodiploid mechanism of sex determination.

## Introduction

Coffee Berry Borer (CBB), *Hypothenemus hampei* (Coleoptera:Curculionidae) is the most devastating insect pest of coffee crops (*Coffea arabica* and *C. canephora*) worldwide. CBB integrated pest management (IPM) has advanced with improvements in sanitation practices and biological control, using both parasitoids and fungal entomopathogens (*Beauveria bassiana*). However, insecticides are still an important component of CBB IPM and a better understanding of CBB genetics, behavior and reproduction promises to improve CBB IPM^1,2^. Here, we describe a whole-genome sequencing effort designed to improve this understanding.

The CBB’s unusual life cycle specifically attacks and destroys developing coffee beans^3^. All of its life stages consume and develop inside the coffee fruit, where they are protected from direct control strategies, such as most entomopathogens and chemical insecticides. Adult females emerge and fly to new fruits where they burrow inside and deposit their eggs. The eggs hatch into small white larvae with an unusual sex ratio: typically, for every 10 females there is only 1 male sibling^4^. Males are less than half the size of females. Male adults are wingless and never leave the coffee berry. Thus, females are restricted to mating with the males already in the fruit before they emerge. Because these males are usually their siblings, CBB populations are highly inbred^5^.

Underlying this unusual life cycle is a highly anomalous genetic system. CBB is functionally haplo-diploid^6^; although both females and males are genetically diploid, males are functionally haploid. Functionally diploid females express and transmit both maternally and paternally derived chromosomes. Functionally haploid males express and transmit only their maternally derived chromosomes. Paternally derived chromosomes in males are silenced by heterochromatization, do not pair with their homologs during meiosis and are not present in sperm nuclei. Functionally analogous systems of paternal genome elimination/inactivation (PGE), have evolved independently in the insect orders Coleoptera, Diptera, Hemiptera, Phthiraptera and Collembola^7^. However, the mechanisms controlling PGE and sex determination in CBB are still unresolved. Earlier investigations have suggested that a presumptive Y chromosome^8^ or the endosymbiotic bacterium *Wolbachia* may play a role^9^.

Several studies have shown that CBB is attracted to coffee berries using visual and olfactory cues^10–12^. Developing coffee berries release volatile compounds that attract female CBBs^12–14^, and field traps have been developed that use methanol and ethanol as olfactory attractants^15,16^. Similarly, CBB-infested coffee berries release volatile compounds that may act as potential repellents that could be used in CBB control^17,18^. Additional research has focused on discovering attractants and repellents in non-host plant species^19,20^ These efforts indicate that olfaction-based technologies are promising methods for the agroecological management of CBB.

Development of future CBB control strategies will require a better understanding of multiple aspects of the insect’s biology. In an attempt to do that, a 163 Mb draft genome of the female CBB was published^21^. The analysis primarily reported the identification of genes that could reveal important aspects in processes such as digestion, detoxification and pathogen defense, as well as the documentation of multiple cases of horizontal gene transfer (HGT) from bacteria into the CBB genome. However, the identification of gene families involved in other important biological aspects remains unexplored. In the present study, we performed de novo sequencing and obtained an improved CBB genome assembly. Unlike the previous assembly, the new assembly identifies male-specific genome sequences. We focused this analysis in the identification of multiple candidate gene families related to chemosensation and plant host finding. Since both sexes were sequenced in this study, we also offer new insights to better understand the mechanism of sex determination in CBB.

## Results and discussion

### Genome sequencing and assembly

We performed a de novo genome sequencing and assembly of CBB using a hybrid approach by combining 454-FLX and Illumina reads from female and male individuals. A total of 3.02 Gb of high-quality 454-FLX sequences and 26 Gb of Illumina sequences were obtained in this study (Table S1), which represent approximate 19 × and 160 × genome coverage respectively based on a previously estimated CBB genome size of 163Mb^21^. The genome hybrid assembly approach we used involved an initial pre-assembly of the 454FLX data with Newbler and the Illumina data with ABySS^22^, followed by merging of these two pre-assemblies into a single genome consensus with Metassembler^23^. Our final hybrid *H. hampei* CENICAFE_Hham1.1 (Hham1.1) genome assembly had a size of 162.57 Mb, comprising 8198 genome scaffolds (Table 1). This assembly represents an improvement in sequence contiguity, containing a 36.3-Kb contig-N50; 340.2-Kb scaffold-N50 and 4.9 Mb for the largest genome scaffold, compared with a previously published CBB genome assembly^21^, which resulted in contig and scaffold N50 of 10.5-Kb and 44.7-Kb respectively and largest genome scaffold of 440-Kb. The Hham1.1 genome assembly completeness was assessed using Benchmarking Universal Single-Copy Orthologs (BUSCO)^24^. BUSCO recovered 98.22% of the 1066 Arthropoda core gene set, from which 96.25% were complete genes and 2% were fragmented genes (Fig. S1). BUSCO results indicate that almost the entire genome of *H. hampei* was sequenced and de novo assembled in this study.

**Table 1:** *Hypothenemus hampei* genome assembly (CENICAFE_Hham1.1) statistics.

Hybrid Genome assembly	
Total genome assembly	162,571,498 bp
Total ungapped assembly	147,450,652 bp
Scaffolds	8198
Largest scaffold length	4,907,154 bp
Scaffold N50	340,248 bp
Scaffold L50	89
Contigs	14,837
Contig N50	36,294 bp
Contig L50	936
G + C	32.32%

### Transcriptome assembly

Illumina RNA-seq data obtained from whole-body female and male adults were de novo assembled using rnaSPades^25^ and sequence redundancy reduced by CD-HIT^26^. The resulting transcript assembly was composed of 64,244 contigs (available at NCBI TSA accession: GIPB00000000.1). The average transcript length was 1103-bp, transcript N50 of 2145-bp and largest transcript of 26,019-bp. The transcript assembly completeness with BUSCO recovered 99.6% (98.97% completed and 0.65% fragmented genes) of the 1066 Arthropoda core gene set. (Fig. S1). Using TransDecoder^27^, we extracted 35,558 protein-encoding transcripts with full Open Reading Frames (ORFs), from which 33,378 (95%) were annotated against InterPro and NCBI NR proteins. As expected, top BLAST hits were against the Coleoptera species, including *D. ponderosae* (61%) *Sitophilus orizae* (22%), *Anoplophora glabripennis* (3%) and *Tribolium castaneum* (5.7%); whereas the remaining hits were against other insect species (14%).

### Gene prediction and functional assignations

We identified 18,765 gene models encoding 20,801 proteins on the Hham1.1 genome assembly using BRAKER2 gene predictor and all available RNA-seq evidence for *H. hampei* at NCBI. The number of gene models found here for our Hham1.1 assembly is slightly smaller than the previous gene prediction (19,222) performed on the first published *H. hampei* genome draft^21^. Completeness of the Hham1.1 gene set using BUSCO recovered 97.2% (94.1% completed and 3.1% fragmented genes) of the Arthropoda core gene set (Fig. S1). BLASTP found 18,364 (88.3%) Hham1.1 predicted proteins similar (e-value < 1e-8) to the NCBI refseq invertebrate proteins, whereas InterProScan detected 20,576 (98.9%) predicted proteins containing conserved protein domains.

### The repertoire of chemosensation-related genes

**Figure 1:**
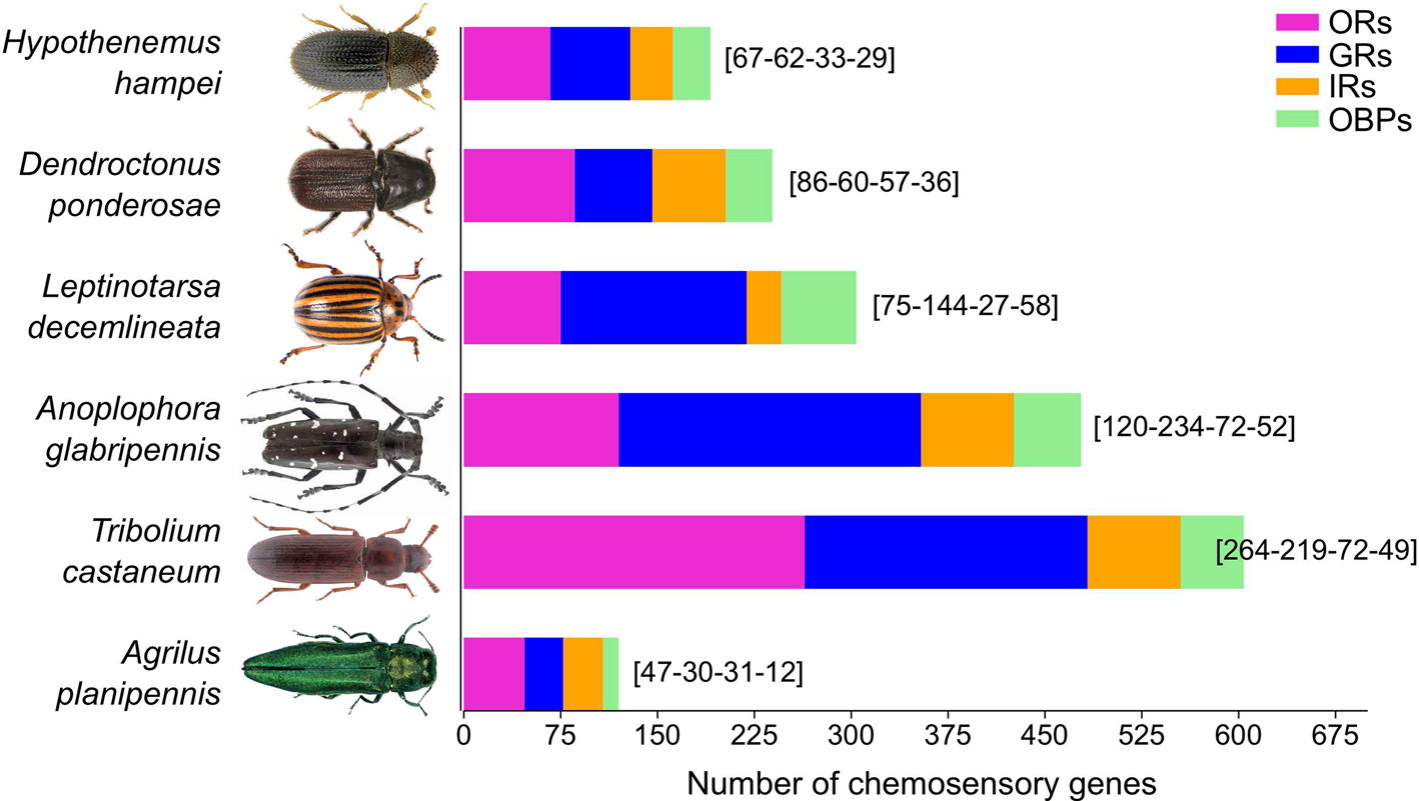
Number of chemosensory genes in Coleoptera insects. The digits in brackets close to histograms represent the number of odorant receptors (ORs), gustatory receptors (GRs), ionotropic receptors (IRs) and odorant-binding proteins (OBPs), respectively, obtained from genome annotations for *Hypothenemus hampei* (this study) (Curculionidae), *Dendroctonus ponderosae* (Curculionidae)^40^, *Leptinotarsa decemlineata* (Chrysomelidae)^49^, *Anoplophora glabripennis* (Cerambycidae)^40^, *Tribolium castaneum* (Tenebrionidae)^42^ and *Agrilus planipennis* (Buprestidae)^40^.

We used the current Hham1.1 genome assembly and available RNAseq data to annotate the major chemosensory-related gene families of *H. hampei*. In insects, chemosensory detection involves genes encoding for mainly three families of transmembrane receptors (chemosensory receptors); odorant (OR), gustatory (GR) and ionotropic receptors (IR). Additionally, a family of small soluble odorant-binding proteins (OBP) are believed to be involved in the olfactory process; however, recent studies have questioned whether they are directly involved in the process and simply modulate both the physiological response and the behavioral response^28^. Since the discovery of the first chemosensation-related genes in insects^29–31^, there has been an increasing interest in these protein/gene families as insect pest control targets due to their functional roles in detection of host/food and mate. Most of the research efforts to identify such insect control targets are mainly focused on OBPs and ORs. Here, we identified and classified most of the members for ORs, GRs, IRs and OBPs encoding genes within the CBB genome. In total, we annotated 191 candidate chemosensory-related genes (Fig. 1 and Supplementary File S1), being one of the most comprehensive gene repertoires identified in the Scolytinae subfamily, along with those from the mountain pine beetle *D. ponderosae* (239 chemosensory-related genes).

#### OR family genes

ORs are membrane-bound heteromeric protein complexes by which insects primarily detect most volatile chemicals. ORs are specific to insects^32,33^ and likely they evolved with the evolution of terrestriality in this group^34^. ORs are mainly expressed in olfactory receptor neurons (ORNs) within sensory appendages such as antennae and maxillary palps^30^. Olfactory function is provided by heteromultimers involving at least one ligand-specific OR and the coreceptor Orco, which is a highly conserved OR among insects^35,36^. OR gene content in insects varies from 10 in the human body louse *Pediculus humanus humanus*^37^ up to 385 in the leaf-cutter ant *Acromyrmex echinatiorin*^38^. In *H. hampei*, we identified 67 candidate OR-encoding genes (*HhamORs*), including Orco (Table S2). This number is smaller than those found in *D. ponderosae* (87 *DponORs*)^39,40^; *Anoplophora glabripennis* (132 *AglaORs*)^41^ and *T. castaneum* (299 *TcORs*)^40,42^. The variation in OR gene contents across different insect species has been attributed to evolution of OR genes following the birth-and-death model^43^ and are presumably associated with ecological adaptations within insect lineages or species. A large proportion of the *HhamOR* genes are located in tandem arrays over the genome scaffolds (Table S2). Among the candidate *H. hampei* OR-encoding sequences, 50 genes likely encode for full-length ORFs (peptides between 258 and 480 amino acids) based on the presence of predicted start and stop codons and sequence homology with other insect OR proteins in the NCBI nr database. The remaining candidate OR genes are missing the 5′ and/or 3′ ends mostly because incomplete transcript data or they occur in regions of truncated scaffolds. Using all available *H. hampei* transcript assemblies at NCBI, we detected transcript evidence for 43 of the 67 candidate OR proteins (Table S2). The full-length *H. hampei* Orco ortholog (*HhamOrCo*) was identified with high confidence in the CBB genome and transcriptome assemblies due to the high degree of amino acid sequence similarity with Orco proteins from other insect species.

**Figure 2:**
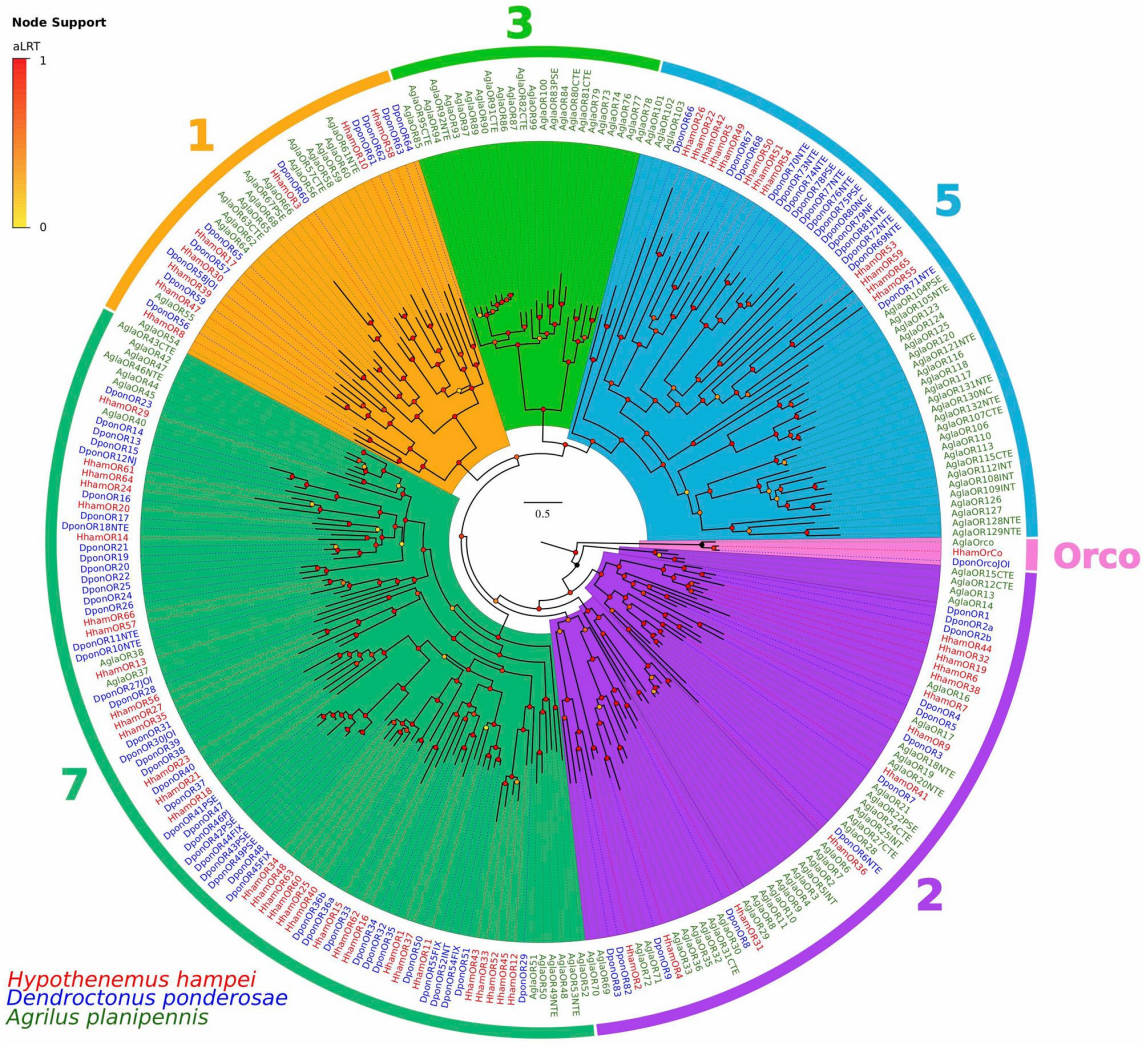
Phylogeny of odorant receptor (OR) family. OR protein sequences from *Hypothenemus hampei* (HhamOR), *Dendroctonus ponderosae* (DponOR) and *Agrilus planipennis* (AplaOR) were clustered by Maximum-Likelihood tree-building. Branch supports (aLRT; approximate likelihood ratio test) are shown as colored circles (yellow to red transition). Colored arcs indicate the clusters for OR families 1–3, 5 and 7; and the conserved OR coreceptor (Orco) clade.

We compared the amino acid sequences of the candidate *HhamORs* with those from the beetles *Dendroctonus ponderosae* and *Anoplophora glabripennis* through a phylogenetic analysis (Fig. 2). Based on previous studies that grouped the coleopteran ORs in seven subfamilies^44,45^, we were able to assign all the *HhamORs* within subfamilies 1, 2, 5 and 7. Similar to *D. ponderosae*, *H. hampei* seems to have lost ORs at least within subfamily 3. The *HhamOrco* clearly clustered with the *DponOrco* and *AglaOrco* proteins into a single clade with high confidence (aLRT = 1.0, Fig. 2), an indication of the high level of protein conservation among these ORs. Most of the *HhamORs* (32) and *DponORs* (46) were grouped into the subfamily 7, consistent with previous phylogenetic analyses that clustered most of the Curculionidae-specific ORs within this OR subfamily^39,40^. Additionally, 23 *HhamORs* clustered along with 29 *DponORs* in a clade within the subfamily 7 that appears to be a OR gene expansion in the Scolytinae^40^. As expected for the close phylogenetic relationship between *H. hampei* and *D. ponderosae*, we found 20 candidate 1:1 orthologous relationships among the Scolytinae ORs and none among the *A. glabripennis* and the Scolytinae ORs.

#### GR family genes

GRs proteins are members of the GPCR superfamily and are phylogenetically related to ORs in insects. This gene family is mainly expressed in gustatory receptor neurons on sensilla distributed on mouthparts, legs, antenna and ovipositors; and are responsible for sensing soluble chemicals by contact. Similar to insect OR gene evolution, GR genes evolve under the birth-and-death model with significant gene content variations among insect lineages^43^; ranging from 5 genes in the obligate mutualistic fig wasp *Ceratosolen solmsi*^46^ up to 431 genes in the German cockroach *Blattella germanica*^47^. In this study, we identified 62 genes encoding for 66 candidate GR proteins (*HhamGRs*) within the *H. hampei* genome and transcriptome assemblies (Table S3). The majority of the *HhamGR* genes identified likely encode for full-length ORFs (peptides between 227 and 461 amino acids), except for *HhamGR57* and *HhamGR58*. Four *HhamGRs* genes presumably encode for two alternative splice variants each (*HhamGR20a*/*b*, *HhamGR21a*/*b*, *HhamGR22a*/*b* and *HhamGR23a*/*b*). Most of the *HhamGR* genes occur in tandem arrays over the genome scaffolds, with the largest array composed by eight genes on scaffold HHAM00005. Transcript evidence was detected for 26 of the candidate *HhamGRs* (Table S3).

**Figure 3:**
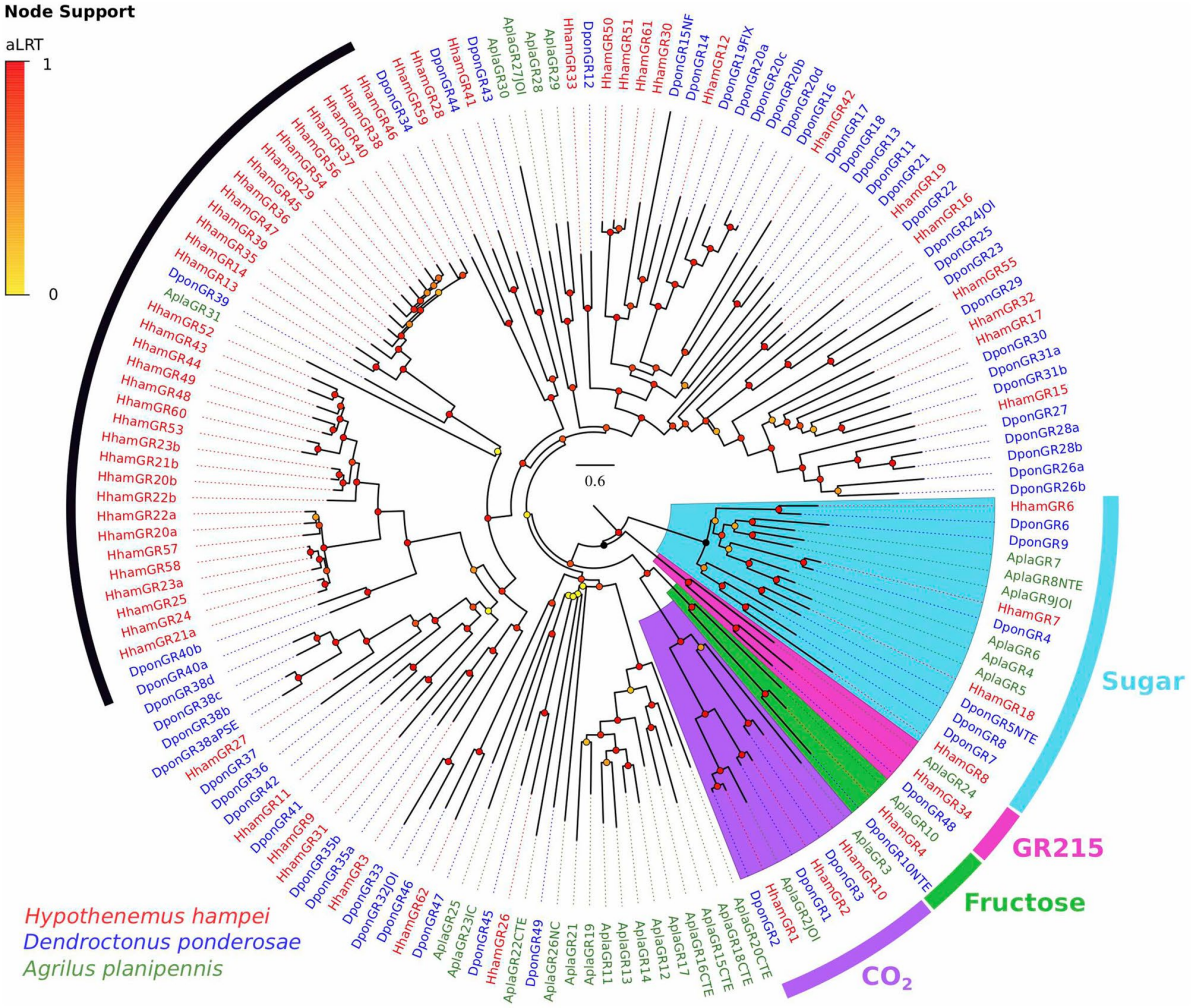
Phylogeny of gustatory receptor (GR) family. GR protein sequences from *Hypothenemus hampei* (HhamGR), *Dendroctonus ponderosae* (DponGR) and *Agrilus planipennis* (AplaGR) were clustered by Maximum-Likelihood tree-building. Branch supports (aLRT; approximate likelihood ratio test) are shown as colored circles (yellow to red transition). Thick colored arcs indicate the clusters for the conserved GRs for fructose, sugar and CO_2_. The remaining GRs are likely bitter receptors. The recently discovered “GR215 clade” is also indicated by a colored arc. The black arc indicates likely GR gene expansions in *H. hampei*.

A phylogenetic analysis of the *HhamGRs*, including the GRs from *D. ponderosae* and *A. planipennis* (*AplaGRs*), identified the putative *HhamGR* members of the most conserved GR families in insects (Fig. 3). Four *HhamGRs* (*HhamGR6*/*7*/*8*/*18*) are placed within the conserved sugar receptor clade, each of them with a likely 1:1 orthologous relationship with *DponGR6*, *DponGR4*, *DponGR8* and *DponGR5*, respectively. Three *HhamGRs* (*HhamGR1*/*2*/*10*) are clustered within the carbon dioxide (CO_2_) receptor clade and appear to be orthologous to *DponGR2*, *DponGR1* and *DponGR3*, respectively, while *HhamGR4* was placed within the conserved fructose receptor clade. The remaining *HhamGRs* are putative bitter-taste GRs forming separate clades between the Scolytinae species and *A. planipennis*, except for the recently discovered “GR215 clade”^40^ that clustered single GR members from each species. Among the putative bitter-taste GRs there are two likely gene expansions in *H. hampei* involving 14 and 19 *HhamGRs*, respectively (indicated by a black arc in Fig. 3), however; the ecological relevance of these *HhamGR* gene expansions will need further investigation. So far, the most comprehensive gene annotations for GR repertoires in Scolytinae beetles are found for *D. ponderosae* (49 *DponGR* genes) and the annotation presented here for *H. hampei* (62 *HhamGR* genes). In perspective with the GR content in other Coleoptera species such as *T. castaneum* (207 GRs)^48^, *Leptinotarsa decemlineata* (144 GRs)^49^, and *A. glabripennis* (234 GRs)^50^, the number of GRs in *H. hampei* and *D. ponderosae* is strongly reduced. Whether or not the repertoire of GRs in Scolytinae has been shrunk during evolution would be resolved when a larger number of Scolytinae species is screened for genome-wide content of GRs.

#### IR family genes

IRs are the most recently discovered chemosensory receptor family, proposed as the “missing” receptor repertoire and originally identified in *Drosophila*^51,52^. IRs are members of the ionotropic glutamate receptors (*iGluRs*) family, expressed in olfactory sensory neurons in the antennae and mouthparts and they respond to different chemical stimuli^52^. In our gene annotation analysis, we identified 33 candidate chemosensory IR-encoding genes (*HhamIR*) within the *H. hampei* genome and transcriptome (Table S4). In insects, IR gene repertoire is enormously variable in numbers, ranging from 10 in the human body louse *P. humanus*^37,53^, up to 604 in the German cockroach *B. germanica*^47,54^. From the annotated *HhamIR* genes, 27 likely encode for full-length ORFs (between 514 and 928 amino acids), whereas the remaining 6 are incomplete genes (between 311 and 832 amino acids) missing 5′ or 3′ ends. Transcript evidence was found for 27 of the candidate *HhamIRs* detected in this study (Table S4).

**Figure 4:**
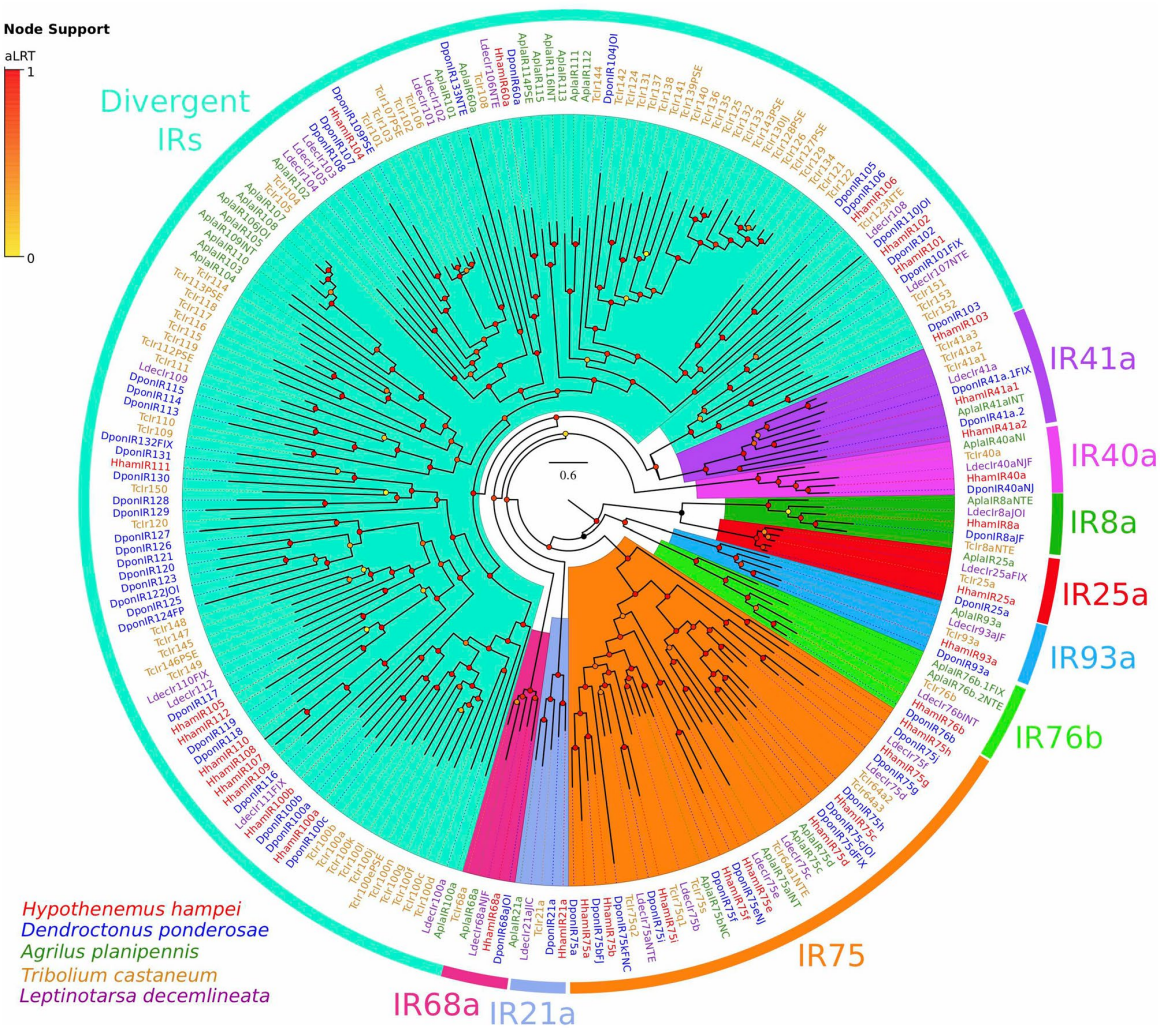
Phylogeny of ionotropic receptors (IR) family. IR protein sequences from *Hypothenemus hampei* (HhamIR), *Dendroctonus ponderosae* (DponIR), *Agrilus planipennis* (AplaIR), *Tribolium castaneum* (TcIr) and *Leptinotarsa decemlineata* (LdecIr) were clustered by Maximum-Likelihood tree-building. Branch supports (aLRT; approximate likelihood ratio test) are shown as colored circles (yellow to red transition). Colored arcs indicate the widely conserved lineages of antennal IRs (IR8a, IR25a, IR21a, IR40a, IR41a, IR68a, IR75 and IR76b) and the Divergent IR clade.

Insect IRs have been classified in two groups; the highly conserved “antennal IRs'' and the species-specific “divergent IRs”^53,55^. Using a phylogenetic analysis for the *HhamIRs*, including those for *D. ponderosae* (*DponIRs*), *A. planipennis* (*AplaIRs*), *T. castaneum* (*TcIrs*) and *L. decemlineata* (*LdecIRs*), we identified *HhamIR* orthologs for all conserved insect antennal IRs (IR8a, IR21a, IR25a, IR40a, IR41a, IR68a, IR75, IR76b and IR93a) (Fig. 4). The repertoire includes single *HhamIR* orthologs for all antennal IRs, except for two paralogs for IR41a (*HhamIR41a1*/*a2*), and nine members for the IR75 group (*HhamIR75a-i*). The remaining fifteen IRs are members of the divergent IRs, a subfamily of IRs that frequently shows species-specific gene expansions^53^. No prominent *HhamIR* gene expansions were detected within the divergent IR subfamily, contrasting with the species-specific gene expansions observed for the other Coleoptera species in the analysis (Fig. 4). Among the divergent IRs, we detected a *HhamIR60a* that grouped with the other conserved Coleoptera IR60a ortholgs with high support (aLRT = 0.97, Fig. 4), and two *HhamIR* members (*HhamIR100a*/*b*) within the IR100 clade (aLRT = 0.96, Fig. 4). The reduced number of divergent *HhamIRs* suggests that members of this group may have been lost and/or no significant diversifying selection is exerting gene expansion events in *H. hampei*.

#### OBP family genes

OBPs are small, extracellular, water-soluble proteins highly abundant in the sensillum lymph of chemosensory organs like the antenna. This protein family is likely involved in the solubilization and transport of odorants through the sensillum lymph^29^. The candidate OBP repertoire found in *H. hampei* (*HhamOBPs*) is encoded by 29 genes (Table S5) with likely full-length ORFs (peptides between 129 and 244 amino acids). Here were also covered 15 partial *HhamOBPs* previously identified in a transcriptome analysis^56^, which are now extended to full ORFs. The assigned OBP names were retained, except for *HhamOBP8.1* (now *HhamOBP3*), *HhamOBP13.2* (now *HhamOBP15*) and *HhamOBP30.1* (now *HhamOBP27*). The majority of the *HhamOBP* genes occur in tandem arrays, as seen in other insect genomes^57–59^, with the largest group composed by 12 genes, located in both DNA directions on a 68-Kb region in genome scaffold HHAM00008. All candidate *HhamOBPs* detected here matched with insect pheromone/OBP domains (PhBP/PBP_GOBP). The secretion peptide, a common structural feature of OBPs, was predicted in all candidate full-length *HhamOBPs*, except *HhamOBP1*, *HhamOBP21* and *HhamOBP25*. Transcript evidence was detected for all candidate OBPs (Table S5). Insect OBP gene repertoire ranges from 5 in the body louse *P. humanus*^37^, up to over 100 in mosquitoes^60^. Previously, OBP repertoires were identified in transcriptome analysis of Scolytinae beetles, such as *Tomicus yunnanensis* (45 OBPs)^61^, *Dendroctonus armandi* (15 OBPs)^61,62^, *D. valens* (21 OBPs)^63^, and *Ips typographus* (15 OBPs)^39^, and recently annotated in the genome of *D. ponderosae* (36 *DponOBPs*)^40^. The number of OBPs in *H. hampei* is not far different from the repertoire of OBPs identified in this species.

**Figure 5:**
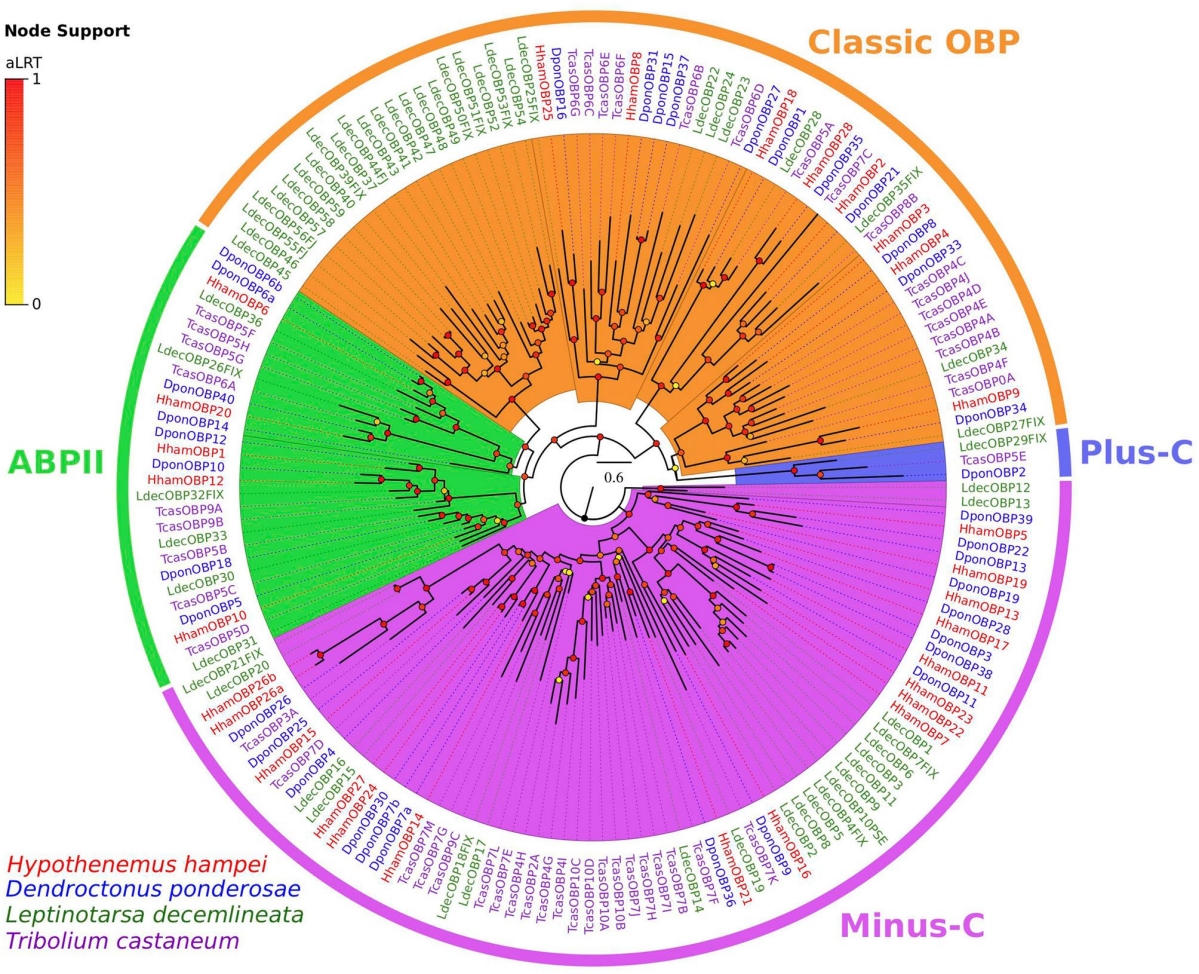
Phylogeny of odorant-binding protein (OBP) family. OBP protein sequences from *Hypothenemus hampei* (HhamOBP), *Dendroctonus ponderosae* (DponOBP), *Leptinotarsa decemlineata* (LdecOBP) and *Tribolium castaneum* (TcasOBP) were clustered by Maximum-Likelihood tree-building. Branch supports (aLRT; approximate likelihood ratio test) are shown as colored circles (yellow to red transition). Colored arcs indicate the conserved OBP classes Minus-C, Plus-C and antennal binding protein II (ABPII). The remaining OBPs are indicated as classic OBPs by an orange arc.

OBPs are classified in three major subgroups or classes: Classic, Plus-C and Minus-C OBPs^43^. The Classic class is recognized by a conserved pattern of six cysteine residues (6C) and includes the pheromone OBPs (POBPs), general OBPs (GOBPs) and antennal binding-proteins (ABPs). Plus-C class commonly shares a conserved pattern of 12 cysteine residues and a proline residue, whereas Minus-C class shares a conserved pattern of four cysteine residues (4C). A phylogenetic analysis of the *HhamOBPs* (Fig. 5), which included OBPs from *D. ponderosae*, *L. decemlineata* and *T. castaneum*, clustered 13 *HhamOBPs* within the Classical subgroup, including 5 ABPs. All “Classic” *HhamOBPs* share the conserved 6C pattern (Supplementary Fig. S2). The remaining 14 *HhamOBPs* were clustered within the Minus-C class and all of them share the conserved 4C pattern (Supplementary Fig. S3). Previous analyses have shown that OBP repertoires from most of the Scolytinae species have members in the Plus-C class^40,61^; however none of the *HhamOBPs* was clustered in this class. This indicates the possibility that members of Plus-C class have been lost in *H. hampei* or OBPs within this class were just missed during the gene annotations.

#### In silico* transcript abundance of chemosensation-related genes in H. hampei*

**Figure 6:**
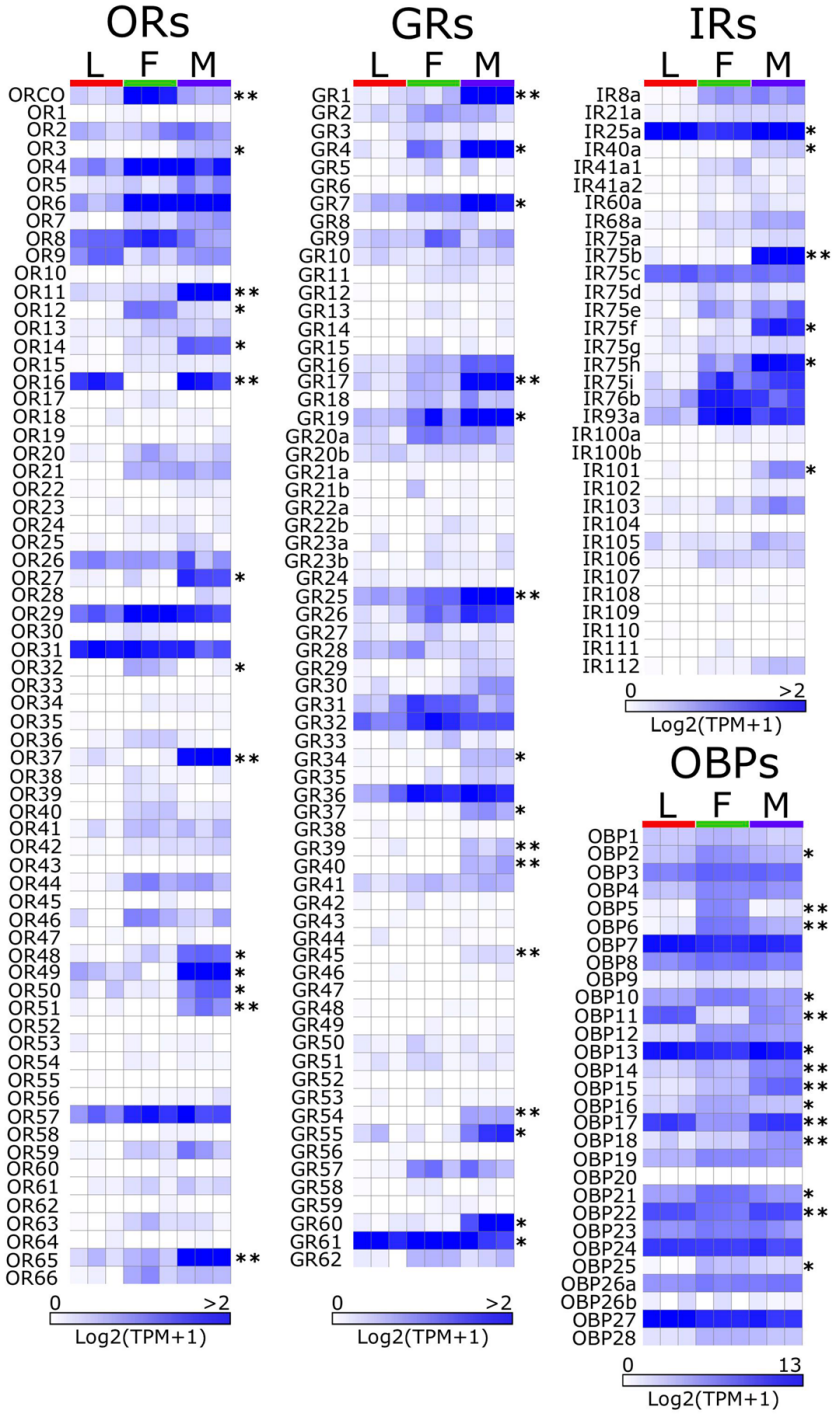
In silico mRNA abundance of chemosensation-related genes. Heat maps represent the abundance of mRNA reads for odorant receptors (ORs), gustatory receptors (GRs), ionotropic receptors (IRs) and odorant-binding proteins (OBPs) in RNA-seq libraries from females (F), males (M) and larvae (L) as estimated by Kallisto. Read abundance is expressed as Log2(TPM + 1). Black asterisks represent chemosensory genes with significant differences at mRNA abundance between females and males as calculated by Kallisto-Sleuth pipeline (False Discovery Rate [FDR] adjusted *p*-value: * < 0.01; ** < 0.001).

We quantified transcript abundance for the annotated *H. hampei* chemosensation-related genes by pseudo-aligning available RNA-seq data from whole-body larva, male and female samples (SRA accessions: SRR7788908—SRR7788916)^56^ against the predicted transcripts using Kallisto v0.46.1 ^64^. Transcript abundance was expressed as Transcripts Per Million (TPM). Mean transcript levels for *HhamORs*, *HhamGRs* and *HhamIRs* were classified as not detected (TPM < 0.02), low (0.02 ≤ TPM < 0.2), moderate (0.2 ≤ TPM < 2) or high (TPM ≥ 2) (Supplementary File S2). Mean transcript levels for *HhamOBPs* were also classified as not detected (TPM < 0.5), low (0.5 ≤ TPM < 50), moderate (50 ≤ TPM < 500) or high (TPM ≥ 500) (Supplementary File S2). Overall, chemosensation-related gene expression in whole-body is mainly enriched in the adults and few are not detected in any of the life stages (Fig. 6, Supplementary File S2). However, we believe that further gene expression analyses should be performed at tissue-specific level, including antennae and palps, for better comparisons and avoid bias toward whole-body gene expression.

Among the *HhamOR* genes, mean transcript abundance ranged from 0 TPM to 18.6 TPM. In the adult male, *HhamOR49* (18.6 TPM), HhamOR11 (10.7 TPM), *HhamOR65* (6.3 TPM), *HhamOR6* (4.3 TPM), *HhamOR37* (3.3 TPM), *HhamOR16* (2.5 TPM), *HhamOR4* (2.5 TPM) and *HhamOR57* (2.1 TPM) are the most abundant ORs with high expression levels (TPM ≥ 2); whereas 27 *HhamORs* genes had moderate expression and the remaining 32 had low or not detected expression. In the adult female, *HhamOR4* (4.8 TPM), *HhamOR6* (3.6 TPM), *HhamOR29* (3.3 TPM), *HhamOrCo* (2.9 TPM), *HhamOR31* (2.5 TPM), *HhamOR57* (2.3 TPM) and *HhamOR8* (2.1 TPM) are the most abundant ORs with high expression levels; whereas 22 *HhamOR* genes had moderate expression and 38 *HhamOR* genes had low or not detected transcript levels. In whole-body larva, *HhamOR31* (2.8 TPM) and *HhamOR16* (2.1 TPM) are the most abundant ORs, whereas only 13 *HhamORs* had moderate expression. The majority of *HhamOR* genes in the larva have low or not detected expression. Between males and females, 14 *HhamORs* are differentially expressed (False Discovery Rate adjusted p-value [q-value] < 0.01), with 11 of them significantly enhanced in the male (*HhamOR3*/*11*/*14*/*16*/*27*/*37*/*48–51*/65) and 3 enhanced in the female (*HhamOR12*/*32*/*OrCo*) (Fig. 6, Supplementary File S2).

Mean transcript abundance for *HhamGR* genes ranged from 0 to 8.7 TPM and are mostly enriched in the adult male (Fig. 6, Supplementary File S2). In the latter, *HhamGR4* (8.7 TPM), *HhamGR19* (7.5 TPM), *HhamGR25* (5.0 TPM), *HhamGR1* (3.8 TPM), *HhamGR60* (3.6 TPM), *HhamGR36* (3.0 TPM), *HhamGR7* (2.8 TPM), *HhamGR17* (2.8 TPM), and *HhamGR61* (2.4 TPM) are the most abundant GRs at high expression levels, whereas 24 are moderately expressed and the remaining 33 *HhamGRs* have low or not detectable levels. In the adult female, only three GRs (*HhamGR61* at 6.4 TPM, *HhamGR36* at 2.5 TPM and *HhamGR32* at 2.3 TPM) are highly expressed, 21 *HhamGRs* are moderately expressed and the remaining 42 have low or not detectable transcript expression. In the larva, most of the *HhamGR* genes (50 GRs) are expressed at low levels or not detected, whereas 15 are expressed at moderate levels, and only *HhamGR61* (3.2 TPM) is expressed at high level. We found 14 differentially expressed (q-value < 0.01) *HhamGR* genes significantly enhanced in the male (*HhamGR1*/*4*/*7*/*17*/*19*/*25*/*34*/*37*/*39*/*40*/*45*/*54*/*55*) and only *HhamGR61* significantly enhanced in the female (Fig. 6, Supplementary File S2).

Among the *HhamIR* genes, mean transcript abundance ranged from 0 to 4.8 TPM (Supplementary File S2). *HhamIR* expression in the male is high for 4 genes (*HhamIR25a*, 4.8 TPM; *HhamIR75b*, 3.1 TPM; *HhamIR75h*, 2.9 TPM; and *HhamIR75f.* 2.2 TPM), moderate for 16 genes and low or not detected for 13 genes (Fig. 6). *HhamIR* expression in the female is high for three genes (*HhamIR93a*, 2.8 TPM; *HhamIR76b*, 2.3 TPM; and *HhamIR25a*, 2.0 TPM), moderate for 12 genes and low or not detected for 18 genes. The majority of *HhamIR* genes in the larva (28 genes) have low or not detected expression; 4 genes have moderate expression and only one is expressed at high levels (*Hham25a*, 3.8 TPM). We also found that 6 *HhamIR* genes are significantly enhanced in the male (*HhamIR25a*/*40a*/75b/75f./75 h/*101*) in comparison to female (Fig. 6, Supplementary File S2).

Overall, mean transcript levels of *HhamOBP* genes (ranging from 0 to 7930 TPM) are higher than chemosensory receptor genes, as already seen in other insect species (Fig. 6, Supplementary File S2). Collectively, 4 Minus-C class *HhamOBP* genes (*HhamOBP27*, *HhamOBP13*, *HhamOBP7* and *HhamOBP24*) are the most abundant with high transcript levels in all life stages. In the male, 9 *HhamOBPs* have moderate expression and 14 have low or not detectable levels; whereas in the female, 12 have moderate expression and a similar number have low or not detectable transcript levels. In the larva, the majority of *HhamOBPs* (20 genes) have low or not detectable transcript levels. Between male and female, we found 14 *HhamOBP* genes differentially expressed, 7 of them significantly enhanced in the male (*HhamOBP11*/*13*/*14*/*15*/*17*/*18*/*22*) and the remaining 7 enhanced in the female (*HhamOBP2*/*5*/*6*/*10*/*16*/*21*/*25*) (Fig. 6, Supplementary File S2).

Differences in the expression levels of chemosensation-related genes between sexes of *H. hampei* could be associated to sex-specific biological roles. Earlier studies demonstrated that CBB females are attracted to volatiles released by developing coffee berries^11–13^. Etiologically, the CBB female is the only life stage with the capacity to search for new host plants since the male is apterous and never leaves the infested coffee berry^4^. These observations suggest there might be differences in olfactory-related gene expression patterns between male and female adults of *H. hampei* associated with host-finding. Among the differentially expressed chemosensation-related genes found here (Fig. 6, Supplementary File S2), several *HhamORs* and *HhamOBPs* were the only female-biased chemosensory genes, likely associated with the host plant finding process. ORs and OBPs are two important protein families associated with volatile detection in insects through the olfactory receptor neurons, primarily located in the antenna^65–67^. However, further gene expression analyses at tissue-level and functional analysis will be necessary to better understand the role of specific genes in host-attraction.

Interestingly, most of the differentially expressed chemosensory receptors (*HhamORs*, *HhamGRs* and *HhamIRs*) are male-biased. For example, 14 *HhamGRs* were significantly enhanced in the male and only one in the female. We speculate that some of these male-enriched chemosensory genes could be involved in male-specific biological processes such as female recognition for mating. Due to the cryptic habits of the CBB male and sib-mating behavior within the infested coffee bean^3^ that likely makes impractical any control based on mating disruption, there has not been interest in the study of chemical signals for reproductive attraction in this species. However, it has been proposed that the CBB male may use contact pheromones as cue for mating recognition based on the observation that the CBB male touches the female’s pronotum and elytra with his antennae and rostrum during the precopulatory phase^68^, a mechanism used by other coleopterans for mate recognition at short distances^69,70^. It is likely that CBB male use gustatory and olfactory signals for female recognition as seen in those insect species. Future research on these questions could bring a better understanding of the relationship between chemical communication and mating behavior in the CBB.

### Male-specific genome sequences: evidence for a presumptive Y-chromosome

#### Evidence of male-specific genetic content

CBB and its relative tropical nut borer (*Hypothenemus obscurus*) are the only two species in Coleoptera with evidence for heterochromatization of the paternally derived genome (paternal genome elimination, PGE) in somatic tissues of males^71^. In these species, PGE is the only evident genetic characteristic influencing sex determination; however, the mechanisms controlling PGE in CBB is still unknown. Early cytological observations found that female metaphase appeared to contain 2n = 14 chromosomes, whereas the male appeared to contain 2n = 14 + 1 ^8^. The extra chromosome in males was presumed as a male determining Y-chromosome, whose frequent loss during spermatogenesis would explain the disproportionate female:male (~ 10:1) ratio. However, later cytological analysis found no evidence of a Y-chromosome in male’s somatic tissues^6^. Due to these confounding previous observations, we decided to make cytological preparations from male gonads and from embryos looking for any evidence of an extra chromosome in these tissues. Meiotic chromosome spreads from males showed two types of metaphase I cells; either containing 14 or 15 chromosomes (Supplementary Fig. S4). Metaphase II cells in males also showed two types of haploid chromosomal patterns with either 7 or 8 chromosomes. In mitotic chromosome preparations from pooled embryos we also observed two types of metaphases containing either 14 or 15 chromosomes (Supplementary Fig. S4). Although we did not estimate the ratio of cells with 14 and 15 in any of the tissue preparations, the proportion of cells with 2n = 14 was clearly larger in all the cases. Thus, our observations suggest the existence of an extra chromosome in males, as previously reported^8^.

#### Detection of male-specific genome scaffolds

**Figure 7:**
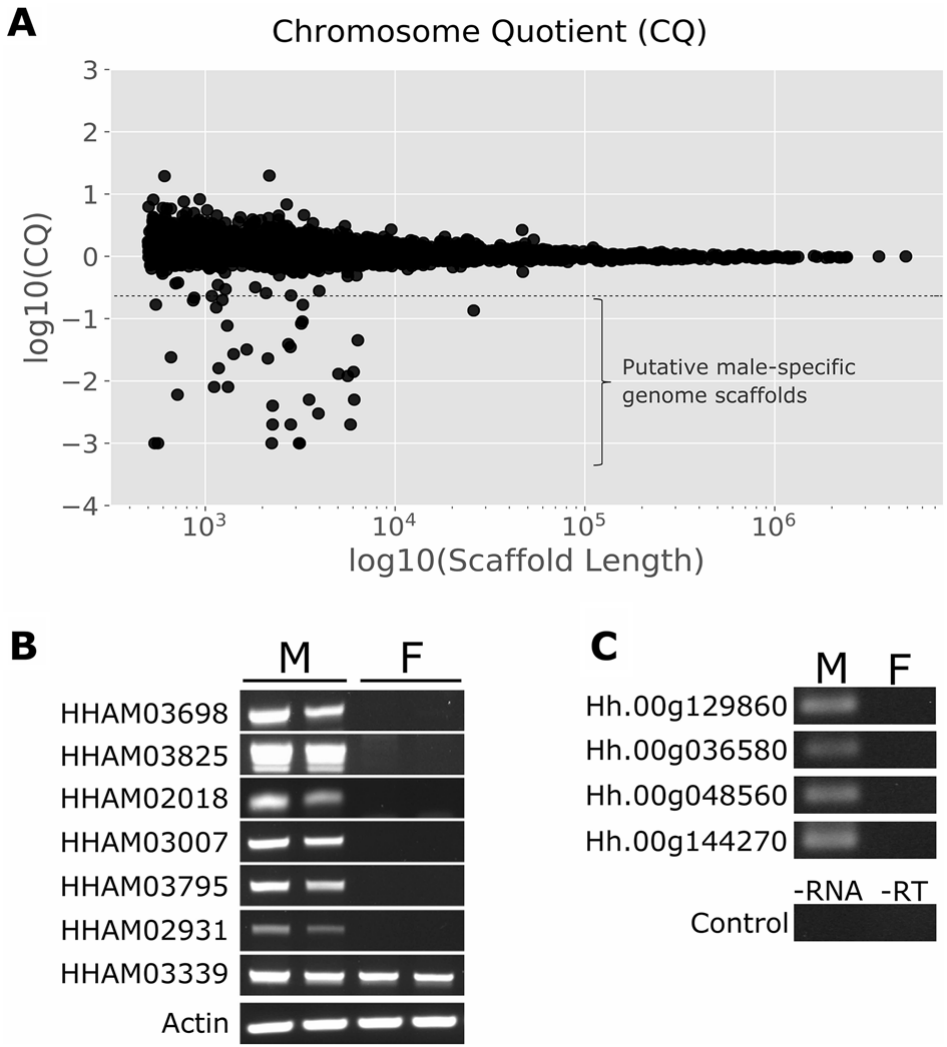
Identification of male-specific genome scaffolds. (**A**) The Chromosome Quotient (CQ) was plotted as Log10(CQ) across the genome scaffolds [Log10(Scaffold Length)]. Each black dot represents the Log10(CQ) of single scaffolds. Dots below − 0.7 (threshold indicated by a dotted line) were considered as male-specific. (**B**) PCR-DNA marker analysis for selected candidate male-specific scaffolds using genomic DNA from males (M) and females (F). Cropped images from several electrophoresis gels were combined for B. The full-length electrophoresis gel images are shown in Supplementary Fig. S5. (**C**) RT-PCR for candidate Y-linked genes using total RNA from males (M) and females (F). Control RT-PCR assays for gDNA contamination were performed using primers for Hh00g129860, with no male-RNA template (-RNA) or lacking retro-transcriptase enzyme (-RT). Cropped images from the same electrophoresis gel were combined for C. The full-length electrophoresis gel image is shown in Supplementary Fig. S6.

Using our CBB genome reference, we exploited the sex-specific genome Illumina reads to detect putative Y-chromosome genomic scaffolds in the *H. hampei* genome. To do that, we implemented the Chromosome Quotient (CQ) method^72^ to estimate the female to male ratio of the number of perfect read alignments to the CBB genome scaffolds. Based on these CQ values, autosomal scaffolds are expected to have no differences in total number of read alignments (CQ ≈ 1); however, Y-chromosome scaffolds should be mapped exclusively by male reads (CQ ≈ 0). Using a threshold of CQ ≤ 0.2 we identified approximately 120-Kb of genome sequences distributed in 36 scaffolds (average size of 3.3-Kb and range of 0.5–26-Kb) as presumptive Y-chromosome sequences (Fig. 7A and Supplementary Table S6). Male-specificity for 7 representatives of the candidate Y-scaffolds was additionally tested by PCR amplification. We selected these scaffolds for further testing since they showed potential protein-coding sequences with BLASTx hits against known proteins in the nr database (see the section “Gene annotation in candidate Y-chromosome scaffolds”). Specific primers for DNA regions on six of these scaffolds successfully amplified the expected DNA bands on male genomic DNA but failed to produce DNA bands on female genomic DNA samples (Fig. 7B and Supplementary Figure S5). Thus, these candidate Y-linked scaffolds were confirmed as genome sequences present only in the *H. hampei* male genome. The other selected scaffold (HHAM03339) unexpectedly amplified a DNA band on both sexes. A closer look revealed that the primers used to test this scaffold rest on a DNA region that presumably contain a repetitive sequence (as discussed below and shown in Supplementary Table S7), which could have caused non-specific PCR bands on both samples. Nonetheless, further cytological confirmation to locate these male-specific scaffolds on a presumptive Y-chromosome using in situ hybridization will be necessary.

Animal Y-chromosomes are frequently rich in repetitive sequences^73^. In one extreme example, *Anopheles gambiae* Y-chromosome contains massively amplified satellites and retrotransposons that count for ~ 98% of its ~ 180-Kb sequence^74^. Compared with the whole *H hampei* genome, which contains ~ 8% of repetitive sequences^75^, the candidate *H. hampei* Y-scaffold sequences contain ~ 55% (66-Kb) of repetitive elements as detected by the RepeatMasker analysis (Supplementary Table S3). Within those, the long interspersed nuclear elements (LINE); a group of non-LTR (long terminal repeat) retrotransposons, were the most abundant transposable elements (~ 90%). The remaining repetitive elements corresponded mostly to different DNA transposon superfamilies (TcMariner-like, Sola-1 and PIF-Harbinger-like) and variable tandem repeats. Interestingly, it has been reported that in the CBB male, transcripts encoding for transposon element (TE)-related proteins are overexpressed when compared with the female^56^. As previously reviewed^76^, TEs are mainly expressed in the germline of animals and differences in genomic imprinting between males and females could influence differences in TE activity.

#### Gene annotation in candidate Y-chromosome scaffolds

The sequences of the 36 candidate Y-scaffolds were submitted to BLASTx searches for detection of protein-coding sequences. Excluding those DNA regions with hits against repetitive elements, BLASTx detected potential protein-coding sequences in ten scaffolds (Table 2). Using ab initio gene predictor Fgenesh^77^, we found full gene structures in four of these scaffolds, which correspond to gene models in the BRAKER prediction (Table 2). Gene model Hh.00g144270 located on scaffold HHAM03795 likely encodes for a candidate protein containing the Jumonji-C (JmjC) and Jumonji-N (JmjN) domains. Both domains are typically found together in JmjC + N histone demethylases, a family of lysine-specific demethylases that regulate chromatin functional state^78,79^. The product of this gene model showed 73% similarity to the *T. castaneum* predicted lysine-specific demethylase 4B (Accession: XP_974381). Gene models Hh.00g036580 and Hh.00g048560, located on scaffolds HHAM03007 and HHAM02931 respectively, likely encode for two different serine/threonine-protein kinases. The product of Hh.00g036580 is 59% similar to the *Trichogramma pretiosum* serine/threonine-protein kinase PAK 3 protein (Accession: XP_014231282.1), whereas the product of Hh.00g048560 is 49% similar to the *D. ponderosae* serine/threonine-protein kinase 10-like protein (Accession: XP_019772232.1). Protein kinases are involved in regulation of protein or enzyme activity within a large number of cellular processes, including modifications of chromatin structure. Finally, gene model Hh.00g129860 on scaffold HHAM03825 likely encodes for a protein containing F-box and WD-repeat domains. This protein is 53% similar to the *T. castaneum* Fbox/WD Archipelago protein (Accession: NP_001164280.1). Fbox/WD proteins are part of the SCF (Skp-Cullin-F-box) complex that function as E3 ubiquitin ligases involved in protein degradation and protein functional modifications throughout ubiquitination. To obtain evidence of the expression of these candidate Y-linked genes, we performed RT-PCR on total RNA samples from males and females (Fig. 7C and Supplementary Figure S6). RT-PCR analysis detected mRNA expression for all four genes in the males but failed detection in the females, consistent with active genes within male-specific genome sequences.

**Table 2:** Candidate Y-linked genome scaffolds with detected putative protein-coding sequences.

Scaffold	Length	CQ^#^	BLASTx best hit^&^	E-value	Similarity (%)
HHAM02931	4064	0.000	Serine/threonine-protein kinase 10 [*Agrilus planipennis*] (XP_018327866)	4e−35	55
HHAM03007	3944	0.003	Serine/threonine-protein kinase PAK3 [*Sitophilus oryzae*] (XP_030749682)	2e−84	47
HHAM03698	3204	0.083	Uncharacterized protein LOC111419136 [*Orthophagus taurus*] (XP_022907672)	6.5e−45	33
HHAM03795	3138	0.001	Lysine-specific demethylase 4B [*Tribolium castaneum*] (XP_974381)	6e−42	84
HHAM03825	3121	0.001	Archipelago [*Tribolim castaneum*] (NP_001164280)	1e−117	45
HHAM06836	1317	0.007	Zinc finger protein 271-like [*Cyprinus carpio*] (XP_018970070)	1e−6	43
HHAM04470	2741	0.039	Zinc finger protein 271-like [*Cyprinus carpio*] (XP_018970070)	4e−6	43
HHAM00577	25,934	0.135	Hypothetical protein FQR65_LT17964 (KAF5269544)	1e−22	52
			Uncharacterized protein LOC115889429 (XP_030765277)	8e−20	36
HHAM02018	6036	0.014	Uncharacterized protein LOC111419136 [*Orthophagus taurus*] (XP_022907672)	6e−71	38
HHAM06939	1229	0.199	Uncharacterized protein LOC107398934 [*Tribolim castaneum*] (XP_015840004)	1e−47	34

^#^Chromosome Quotient (CQ).

^&^BLASTx search against the NCBI reference protein database.

Studies about chromosome imprinting and PGE in the mealybug *Planococcus citri* have shown that changes in histone modification and colocalization with HP1 protein (heterochromatin protein 1) are involved in heterochromatization of the paternal chromosomes in males^80,81^; however, the mechanism that triggers those imprinting marks has not been elucidated yet. Evidence for differential chromosome imprinting between CBB males and females and its relationship with PGE in males is still awaiting. Histones are subject to multiple posttranslational modifications including methylation, acetylation, phosphorylation and ubiquitination that can affect chromatin architecture and determine chromatin state and gene transcription^82^. Methylation of Lys 27 and Lys 9 of histone H3 (H3K27me3 and H3K9me3, respectively) are involved in the formation of heterochromatin^83,84^. Histone phosphorylation is best known for its function in cellular response to DNA damage; however, it plays crucial roles in chromatin relaxation/compaction and segregation during mitosis and meiosis^85^. Histone ubiquitination is heavily involved in regulation of chromatin and control of chromatin-related processes such as DNA damage repair, transcription regulation and the maintenance (“memory”) of epigenetic marks during mitosis^86^. Whether the product of candidate Y-linked genes in *H. hampei* could be involved in modifications of chromatin architecture or functional state is still unresolved. Further functional analysis based on gene editing or RNA interference may be useful to elucidate the putative role of these candidate Y-linked genes in sex-determination and male development.

## Conclusions

This genome assembly and analysis of CBB provides novel biological insights for the most relevant insect pest for coffee. We present a de novo genome assembly with improved sequence contiguity based on a hybrid assembly approach that includes 454-FLX and Illumina shotgun data. We have identified multiple candidate gene families necessary to understand the mechanism of host-plant attraction to offer future possibilities for exploration of olfaction-based control strategies. The repertoire of chemosensation-related genes identified in *H. hampei* (191 genes) is relatively small in comparison with the Scolytinae beetle *D. ponderosae* (239 genes) and the Coleoptera model species *T. castaneum* (573 genes), which represent a good opportunity for future molecular functional studies in the CBB olfaction process. Furthermore, differential gene expression patterns of chemosensory genes are likely associated with the pronounced behavioral differences in host-finding between CBB males and females. The reduced number of GR-encoding genes identified in *H. hampei* also supports the idea that this family of chemosensory proteins was reduced during evolution within the Scolytinae clade and may not represent major ecological relevance in bark beetles. Finally, the detection of male-specific genome sequences provides evidence for a presumptive Y-chromosome in CBB, which may be involved in sex determination in accordance with earlier findings.

## Methods

### De novo genome sequencing and assembly:

Insects used for DNA isolation were collected from a CBB population that have been maintained in the laboratory as an inbred line for more than ten years at the National Coffee Research Center—CENICAFE (Colombia). High-quality genomic DNA samples for males and females were extracted from pooled adults using DNeasy Tissue Kit (Qiagen). Whole genome sequencing was performed using Roche 454-FLX Titanium and Illumina HiSeq2500 platforms. 454-FLX single-end (SE) libraries were prepared from males and females separately, whereas a 454-FLX 20 Kb-insert mate-pair (MP) library was prepared only from females. 454-FLX library construction and sequencing were performed at the Colombian National Center for Sequencing (Antioquia State University, Medellin, Colombia). A short-insert 350 bp paired-end (PE) Illumina library from males and a 1.5 Kb-insert MP Illumina library from females were constructed and sequenced at the Purdue University Genomics Core (West Lafayette, IN). Raw Illumina reads were adaptor-removed, trimmed and filtered according to quality using default parameters with the Fastx-Toolkit v.0.014.

Whole genome assembly was performed using a hybrid approach involving the 454 and Illumina genome sequencing data produced in this study. GS De Novo Assembler (Newbler) was used to pre-assemble the 454 sequencing reads with default settings. ABySS assembler^22^ was used to pre-assemble the Illumina sequencing reads with default settings. Then, we used Metassembler to create a final draft genome consensus by merging the 454-FLX and Illumina pre-assembled scaffold sequences. For removal of sequence contamination, we ran a BLASTn search against the NCBI nucleotide database. After taxonomic inspection of significant hits (≥ 95% identity over 100 bp length), fungal and bacterial contaminating contigs were removed from the CBB genome assembly. Completeness of the genome assembly was estimated with BUSCO^24^ using the Arthropod ortholog dataset.

### RNA-seq and transcript assembly

Insects used for RNA-seq were collected from the same laboratory population described above and reared on ~ 70% humidity coffee parchment. Total RNA was isolated from pooled whole-body female and male adults (30 and 50 individuals, respectively), separately, using RNeasy Mini Kit (Qiagen) and including a DNase I step to remove genomic DNA contamination. Illumina RNA-seq single-end library construction using TruSeq RNA Library Prep Kit v2 and sequencing through a HiSeq2500 platform were performed by BGI (Hong Kong). Raw Illumina reads was adaptor-removed, trimmed and filtered according to quality using default parameters of the Fastx-Toolkit v.0.014. Transcript assembly was performed using rnaSPades v.3.14.0^25^ with default parameters. Transcript redundancy was reduced by clustering sequences with CD-HIT v.4.8.1^26^ at default options. Removal of sequence contamination was performed using BLASTn search as described above.

### Gene prediction

Protein coding genes in the CBB genome assembly were predicted under the GenSAS annotation platform^87^ using the following pipeline. RepeatMasker^88^ was used to screen and mask interspersed repeats and low complexity DNA sequences over the genome assembly. Full gene structure annotations were performed using BRAKER2 v.2.1.0^89^. RNA-seq reads produced in this study (SRA accessions: SRR11858905, SRR11858906) and others publicly available datasets at NCBI for *H. hampei* (SRA accessions: SRR7788908—SRR7788916, SRR2163439) were mapped against the reference genome using HISAT2 v.2.1^90^ and used for gene prediction training and final evidence for gene annotation within BRAKER2. Functional annotation of predicted genes was performed by identification of conserved protein domains using InterPro and BLASTP against the NCBI refseq invertebrate proteins.

### Identification and annotation of chemosensory family proteins

Available full protein sequences for OR, GR, IR and OBP from insects species *Dendroctonus ponderosae, Agrilus planipennis*^40^, *Tribolium castaneum* (Uniprot proteome ID: UP000007266), and *Drosophila melanogaster* (Uniprot proteome ID: UP000000803) were used as references for homology-based sequence finding with genBlastG (She et al., 2011) against the CBB genome scaffolds and TBLASTN search against the transcriptome sequences. The predicted chemosensory protein sequences from CBB and those from *D. ponderosae* were used as reference for new searches against CBB genome scaffolds and transcriptome as described above until no further new protein sequences were obtained. The candidate sequences were confirmed by BLASTP against the non-redundant protein sequences from NCBI. Phylogenetic analyses for each gene family were performed with SeaView (v4.0)^91^ as follows: multiple protein alignments were performed with Muscle and tree-building with Maximum-Likelihood (PhyML) method^92^ implemented with LG model. Branch supports were estimated using Approximate Likelihood Ratio Test (aLRT)^93^.

### In silico mRNA abundance of chemosensory genes

Transcript reads from whole-body female, male and larva RNAseq data previously published^56^ and available at the NCBI SRA database (SRA accessions: SRR7788908—SRR7788916) were processed with Trimmomatic v.0.39 ^94^ for adapter removal (MINLEN:50, ILLUMINACLIP:/TruSeq2-PE.fa:2:30:10:2:keepBothReads). Transcript abundances (expressed as TPM) were obtained by pseudo-aligning the filtered RNAseq reads from each life stage against *H. hampei* transcript reference using Kallisto v0.46.1 (option: -b 100)^64^. The transcript reference was prepared using the *H. hampei* BRAKER predicted coding-sequences (CDS), where the corresponding BRAKER sequences were replaced with the *H. hampei* chemosensory CDS sequences. Identification of differentially expressed chemosensory genes was performed using Sleuth^95^ with a likelihood ratio test according to the pipeline in the officially supported walkthroughs (https://pachterlab.github.io/sleuth_walkthroughs/trapnell/analysis.html). Statistical significance was established using false discovery rate (FDR) adjusted p-value (α = 0.01). Transcript abundance heatmaps were plotted with Matrix2png (https://matrix2png.msl.ubc.ca/) and edited with InkScape v1.0 (https://inkscape.org).

### Chromosome preparations

Gonads from adult CBB males were dissected in 1 × PBS buffers under a stereoscope. CBB embryos and gonads were transferred to 30 μL of hypotonic solution (68.44 mM NaCl; CaCl_2_ 0.90 mM; 1.34 mM KCl; 1.19 mM NaHCO3) containing 1% colchicine in a microcentrifugue tube. Tissues were gently disrupted in this solution using a pestle and incubated during 3 h in the dark. Tissues were fixed by adding 200 μL of ethanol:acetic acid (4:1) (fixative solution) and incubation for 2 min. The tissue was collected by centrifugation (3000 rpm, 4 min) and washed in a new fixation solution, repeating the process twice. The samples were finally resuspended in a 20 μL fixation solution and spreaded on microscope slides. Chromosome preparations were stained with 5% giemsa or DAPI. Images were registered under light and fluorescence microscopes.

### Detection of male-specific genome scaffolds

Identification of putative male-specific genome scaffolds was performed using the Chromosome Quotient (CQ) method^72^. First, repetitive sequences were masked from the CBB genome scaffolds using RepeatMasker^88^. Then, the genome Illumina reads from the female (NCBI SRA accession: SRR11579638) and male (NCBI SRA accession: SRR11579639) libraries were mapped as single-end reads against the hard-masked version of the CBB genome assembly using the perl script CQ-calculate.pl^72^ with settings: “-l 100” and “-norm 1.2”, to generate CQ values. We used a threshold CQ ≤ 0.2 to identify putative male-specific scaffolds. Validation for male-specificity of selected genome scaffolds was performed by standard PCR amplification with specific primers (Supplementary Table S8) and PCR products separated by agarose gel electrophoresis.

### RT-PCR analysis of candidate Y-linked genes

Total RNA samples were isolated from separate pools of 15 whole-body CBB males and females using ISOLATE II RNA Mini Kit (BioLine; London, UK) following the manufacturer's protocol and including the genomic DNA digestion step with DNase I. RT-PCR was performed with MyTaq™ One-Step RT-PCR Kit (BioLine) in 20 μL reactions containing 100 ng of total RNA template, 1 × MyTaq One-Step Mix, 10uM of each gene-specific primer (Supplementary Table S4), 0.2 μL reverse transcriptase and 0.4 μL RiboSafe RNase Inhibitor. PCR cycles were performed with 45 °C for 20 min and 95 °C for 1 min, followed by 35 cycles of 95 °C for 10 s, 60 °C for 10 s, and 72 °C for 60 s. RT-PCR products were finally separated by agarose gel electrophoresis.

## Supplementary Information

Supplementary Information.

## Data Availability

All CBB whole-genome and transcriptome sequencing in this study is available under the NCBI BioProject PRJNA626647 (https://www.ncbi.nlm.nih.gov/sra/PRJNA626647). This Whole Genome Shotgun assembly project has been deposited at DDBJ/ENA/GenBank under the accession JABRWK000000000. The version described in this paper is version JABRWK010000000. BRAKER gene prediction and annotation are available at https://osf.io/vgb5e/?view_only=1dc51c5f4964450d97887ba947e814ee.
