# Supplementary material for: A coffee berry borer (Hypothenemus hampei) genome assembly reveals a reduced chemosensory receptor gene repertoire and male-specific genome sequences
Source: Sci Rep. 2021 Mar 1;11:4900. doi: 10.1038/s41598-021-84068-1 (PMC7921381; doi:10.1038/s41598-021-84068-1)
Supplement: Supplementary file 1 — Supplementary Information. [file 41598_2021_84068_MOESM1_ESM.pdf]

A coffee berry borer (*Hypothenemus hampei*) genome assembly reveals a reduced chemosensory receptor gene repertoire and male-specific genome sequences

Lucio Navarro[1*], Erick M. Hernandez-Hernandez[2], Jonathan Nuñez[3], Flor E. Acevedo[4], Alejandro Berrio[5], Luis M. Constantino[1], Beatriz Padilla[6], Diana Molina[1], Carmenza Gongora[1], Ricardo Acuña[1], Jeff Stuart[7], Pablo Benavides[1].

[1]National Coffee Research Center - CENICAFE, Manizales, Colombia.
[2]UNESP-Univ. Estadual Paulista, São José do Rio Preto, SP, Brazil.
[3]Manaaki Whenua – Landcare Research, Lincoln 7640, New Zealand.
[4]Department of Entomology, Pennsylvania State University, University Park, PA, USA.
[5]Department of Biology, Duke University, Durham, USA.
[6]Instituto de Investigación en Microbiología y Biotecnología Agroindustrial. Universidad Católica de Manizales, Manizales, Colombia.
[7]Department of Entomology, Purdue University, West Lafayette, USA.

SUPPLEMENTARY TABLES AND FIGURES:

Supplementary Table S1. Genomic DNA sequencing library statistics:

**Sequencing library**	**Reads**	**Bases**
Single-end 454-FLX female	3,013,872	1.72-Gb
Single-end 454-FLX male	1,244,500	0.75-Gb
Mate-pair 454-FLX female	1,184,589	0.55-Gb
Illumina mate-pair female	69,587,001	14.1-Gb
Illumina paired-end male	58,808,862	11.9-Gb

Supplementary Table S2: Candidate odorant receptors (ORs) genes.

Name	Length (aa)	Genomic position [scaffold:start..end(strand)]	Associated locus	Transcript evidence[#]	Status
HhamOrCo	480	HHAM00180:109692..111690(+)	Hh.00g173970	yes	full
HhamOR1	386	HHAM00170:27228..28608(+)	Hh.00g031880	no	full
HhamOR2	423	HHAM00001:2705458..2707416(-)	Hh.00g170470	yes	full
HhamOR3	385	HHAM00710:6982..8404(-)	Hh.00g165020	yes	full
HhamOR4	409	HHAM00181:40595..42646(+)	Hh.00g026910	yes	full
HhamOR5	382	HHAM00007:828120..829493(+)	Hh.00g111200	yes	full
HhamOR6	379	HHAM03362:488..1905(+)	Hh.00g158330	yes	full
HhamOR7	374	HHAM00005:1358233..1360737(-)	Hh.00g105690	yes	full
HhamOR8	386	HHAM00052:79416..83189(+)	Hh.00g032660	yes	full
HhamOR9	389	HHAM00003:339829..341191(+)	Hh.00g051450	yes	full
HhamOR10	383	HHAM04158:333..2016(-)	Hh.00g010090	no	full
HhamOR11	393	HHAM00129:162423..163806(-)	Hh.00g003510	yes	full
HhamOR12	383	HHAM01718:428..1691(+)	Hh.00g163180	yes	full
HhamOR13	378	HHAM00040:99960..101696(+)	Hh.00g135260	yes	full
HhamOR14	393	HHAM00013:781623..784535(-)	Hh.00g128540	yes	5’ missing
HhamOR15	381	HHAM00011:1293836..1295228(+)	Hh.00g023640	yes	full
HhamOR16	367	HHAM00170:4837..9837(-)	Hh.00g031820	yes	full
HhamOR17	351	HHAM00170:140511..143624(-)	Hh.00g031940	no	full
HhamOR18	375	HHAM01100:1537..2908(-)	Hh.00g084430	no	full
HhamOR19	384	HHAM00049:351646..353077(-)	n.a.	no	full
HhamOR20	383	HHAM00040:121726..123467(+)	Hh.00g135270	yes	full
HhamOR21	402	HHAM01100:7097..8562(-)	Hh.00g084440	yes	full
HhamOR22	373	HHAM00090:195877..198111(+)	Hh.00g074490	no	full

HhamOR23	331	HHAM02680:2179..4058(+)	n.a.	no	full
HhamOR24	302	HHAM00343:56583..60387(-)	Hh.00g125120	no	full
HhamOR25	383	HHAM00768:1301..2721(-)	Hh.00g136690	yes	full
HhamOR26	375	HHAM00079:307217..308872(+)	Hh.00g076140	yes	full
HhamOR27	387	HHAM00023:994446..997231(-)	Hh.00g025830	yes	full
HhamOR28	142	HHAM03502:417..3361(-)	Hh.00g098130	no	5’ missing
HhamOR29	416	HHAM00028:48381..50770(-)	Hh.00g039300	yes	full
HhamOR30	369	HHAM00258:13348..17887(-)	Hh.00g120260	yes	3’ missing
HhamOR31	418	HHAM00001:2943825..2945401(+)	Hh.00g170770	yes	full
HhamOR32	379	HHAM00049:345061..346475(+)	n.a.	yes	full
HhamOR33	288	HHAM02926:178..1112(-)	n.a.	no	3’ missing
HhamOR34	365	HHAM00121:244804..246131(-)	n.a.	yes	full
HhamOR35	326	HHAM00123:34847..36082(-)	n.a.	no	full
HhamOR36	397	HHAM00128:196282..199955(-)	Hh.00g095180	yes	full
HhamOR37	385	HHAM00170:38990..40465(+)	Hh.00g031890	yes	full
HhamOR38	381	HHAM00049:375160..376582(+)	Hh.00g058180	no	full
HhamOR39	258	HHAM00052:45505..46548(+)	Hh.00g032630	no	full
HhamOR40	321	HHAM03550:2188..3306(+)	Hh.00g092710	yes	3’ missing
HhamOR41	349	HHAM01018:372..2488(-)	Hh.00g047090	yes	full
HhamOR42	424	HHAM00090:185140..187423(+)	Hh.00g074480	no	full
HhamOR43	327	HHAM02926:1559..2847(-)	n.a.	no	full
HhamOR44	331	HHAM00049:341487..343452(+)	Hh.00g058140	yes	full
HhamOR45	349	HHAM05338:1119..2312(+)	Hh.00g046620	no	3’ missing
HhamOR46	190	HHAM04728:178..998(+)	n.a.	yes	5’ missing
HhamOR47	393	HHAM00052:47610..51944(-)	Hh.00g032640	yes	full
HhamOR48	382	HHAM00121:239435..241707(-)	Hh.00g046420	yes	full

HhamOR49	373	HHAM00002:2922559..2924227(+)	Hh.00g007870	yes	full
HhamOR50	331	HHAM00002:2908180..2914144(-)	Hh.00g007850	yes	5' missing
HhamOR51	322	HHAM00002:2919939..2921231(-)	Hh.00g007860	yes	5' missing
HhamOR52	344	HHAM00007:1643326..1644643(+)	n.a.	no	3' missing
HhamOR53	357	HHAM00009:1917038..1918527(-)	Hh.00g152270	no	full
HhamOR54	322	HHAM00011:1202803..1203840(+)	n.a.	no	5' missing
HhamOR55	340	HHAM00017:257310..260371(+)	Hh.00g80380	no	full
HhamOR56	363	HHAM00023:989275..992175(-)	Hh.00g025820	no	full
HhamOR57	313	HHAM00040:239177..241933(-)	Hh.00g135380	yes	5' missing
HhamOR58	286	HHAM00049:596006..599725(-)	Hh.00g058440	yes	5' missing
HhamOR59	271	HHAM00088:269964..271167(+)	Hh.00g036880	yes	full
HhamOR60	383	HHAM00138:3145..4567(-)	Hh.00g032400	no	full
HhamOR61	305	HHAM00149:136641..144298(+)	Hh.00g185240	yes	5' missing
HhamOR62	273	HHAM00170:1252..2173(+)	Hh.00g031810	no	3' missing
HhamOR63	308	HHAM00183:110..1316(+)	Hh.00g096270	no	5' missing
HhamOR64	306	HHAM00379:9410..12869(-)	Hh.00g013870	yes	5' missing
HhamOR65	352	HHAM02109:2576..5086(+)	Hh.00g037010	yes	full
HhamOR66	363	HHAM00407:2588..4158(+)	Hh.00g009250	yes	full

n.a: no available

Supplementary Table S3: Candidate gustatory receptors (GRs) genes.

Name	Length (aa)	Genomic position [scaffold:start..end(strand)]	Associated locus	Transcript evidence[#]	Status
HhamGR1	423	HHAM00225:130515..132101(-)	Hh.00g037690	yes	Full
HhamGR2	438	HHAM00005:1335881..1338258(+)	Hh.00g105650	yes	Full
HhamGR3	372	HHAM00008:493969..495143(-)	Hh.00g065120	no	Full
HhamGR4	425	HHAM00124:180008..183009(-)	Hh.00g175310	yes	Full
HhamGR5	227	HHAM00009:1722657..1723389(-)	Hh.00g151930	no	5' missing
HhamGR6	391	HHAM00018:763315..764756(+)	n.a.	no	Full
HhamGR7	418	HHAM00012:126952..129785(-)	Hh.00g179090	yes	Full
HhamGR8	364	HHAM00018:764863..766360(+)	Hh.00g119120	yes	Full
HhamGR9	383	HHAM01151:3947..5145(-)	Hh.00g103560	yes	Full
HhamGR10	461	HHAM00003:2042126..2043873(-)	Hh.00g055240	yes	Full
HhamGR11	368	HHAM00020:412105..413447(+)	Hh.00g049260	yes	Full
HhamGR12	362	HHAM00058:398494..399619(+)	n.a.	no	Full
HhamGR13	338	HHAM00602:16922..18104(-)	Hh.00g137070	no	Full
HhamGR14	393	HHAM01096:297..1611(+)	Hh.00g033490	no	Full
HhamGR15	382	HHAM00004:894907..896145(-)	Hh.00g028250	no	Full
HhamGR16	358	HHAM00039:377817..379242(+)	Hh.00g016240	yes	Full
HhamGR17	389	HHAM00003:842260..843483(-)	Hh.00g052560	yes	Full
HhamGR18	388	HHAM00018:754147..755556(-)	Hh.00g119100	yes	Full
HhamGR19	408	HHAM00583:15006..17693(+)	Hh.00g000770	yes	Full
HhamGR20a	358	HHAM00005:870238..872575(+)	Hh.00g104990	yes	Full
HhamGR20b	350	HHAM00005:871467..872575(+)	Hh.00g104990	yes	Full

HhamGR21a	357	HHAM00005:873335..875684(+)	Hh.00g105000	no	Full
HhamGR21b	350	HHAM00005:874576..875684(+)	Hh.00g105000	no	Full
HhamGR22a	356	HHAM00005:876417..878742(+)	Hh.00g105010	no	Full
HhamGR22b	351	HHAM00005:877638..878742(+)	Hh.00g105010	no	Full
HhamGR23a	359	HHAM00005:858189..860548(+)	Hh.00g104970	no	Full
HhamGR23b	350	HHAM00005:859440..860548(+)	Hh.00g104970	no	Full
HhamGR24	357	HHAM00005:865068..868055(+)	Hh.00g104980	no	Full
HhamGR25	359	HHAM00005:844183..846719(+)	Hh.00g104950	yes	Full
HhamGR26	374	HHAM00005:1273826..1275407(+)	n.a.	yes	Full
HhamGR27	346	HHAM00011:1621423..1622626(-)	Hh.00g023850	yes	Full
HhamGR28	378	HHAM00016:791049..792908(-)	Hh.00g091540	yes	Full
HhamGR29	376	HHAM00035:619081..620262(-)	Hh.00g061290	no	Full
HhamGR30	341	HHAM00033:190620..194652(-)	Hh.00g093350	no	Full
HhamGR31	388	HHAM00573:1390..2599(-)	Hh.00g102260	yes	Full
HhamGR32	364	HHAM00079:229484..232212(-)	Hh.00g076060	yes	Full
HhamGR33	337	HHAM00082:339593..340658(-)	Hh.00g089950	no	Full
HhamGR34	433	HHAM00086:277428..279854(-)	Hh.00g182510	yes	Full
HhamGR35	369	HHAM00104:1067..2230(-)	Hh.00g108650	yes	Full
HhamGR36	337	HHAM00120:57933..58999(+)	n.a.	no	Full
HhamGR37	371	HHAM00120:79856..80868(+)	n.a.	no	Full
HhamGR38	323	HHAM00210:74776..75935(+)	n.a.	no	Full
HhamGR39	345	HHAM00229:111428..112597(-)	n.a.	no	Full
HhamGR40	346	HHAM00229:109364..110524(-)	n.a.	no	Full

HhamGR41	358	HHAM00244:74461..75534(-)	Hh.00g071600	no	Full
HhamGR42	334	HHAM00314:4657..5781(-)	n.a.	no	Full
HhamGR43	359	HHAM00485:548..1689(+)	Hh.00g092130	no	Full
HhamGR44	355	HHAM00571:24049..25171(-)	Hh.00g073820	no	Full
HhamGR45	374	HHAM00620:4773..5948(-)	n.a.	no	Full
HhamGR46	382	HHAM00620:17180..18378(-)	n.a.	no	Full
HhamGR47	371	HHAM00620:22439..23605(-)	n.a.	no	Full
HhamGR48	352	HHAM00634:6301..7413(-)	Hh.00g029880	yes	Full
HhamGR49	339	HHAM00634:22222..23296(-)	Hh.00g029890	no	Full
HhamGR50	348	HHAM00860:8967..10285(-)	n.a.	no	Full
HhamGR51	347	HHAM00860:12121..13430(-)	n.a.	no	Full
HhamGR52	325	HHAM00955:3922..5043(-)	n.a.	no	Full
HhamGR53	349	HHAM02269:3907..5025(+)	Hh.00g086400	no	Full
HhamGR54	378	HHAM03169:1251..2437(+)	Hh.00g173610	no	Full
HhamGR55	350	HHAM00018:532426..533521(+)	Hh.00g118870	yes	Full
HhamGR56	355	HHAM01837:1470..2645(+)	n.a.	no	Full
HhamGR57	270	HHAM00005:847,763..848,583(+)	Hh.00g104960	no	3'missing
HhamGR58	257	HHAM00005:855,731..856,616(-)	Hh.00g104960	no	3'missing
HhamGR59	373	HHAM00008:515706..517001(+)	Hh.00g065140	no	Full
HhamGR60	346	HHAM00165:12,666..13,794(+)	Hh.00g024370	yes	Full
HhamGR61	339	HHAM00860:14,406-15,717(-)	Hh.00g165930	yes	Full
HhamGR62	401	HHAM00220:38,654..40,304(-)	n.a.	no	Full

[#] n.a: no available

Supplementary Table S4: Candidate ionotropic receptors (IRs) genes.

Name	Length (aa)	Genomic position [scaffold:start..end(strand)]	Associated locus	Transcript evidence[#]	Status
HhamIR8a	840	HHAM00148:143817..147538(-), HHAM03495:967..2553(-)	Hh.00g092280, Hh.00g181790	yes	full
HhamIR21a	671	HHAM00016:589935..592387(-)	Hh.00g091280	yes	full
HhamIR25a	928	HHAM00004:1247..4715(-)	Hh.00g027250	yes	full
HhamIR40a	832	HHAM00005:1361122..1365705(-)	Hh.00g105700	yes	5' missing
HhamIR41a1	589	HHAM00008:714082..716206(-)	Hh.00g065180	yes	full
HhamIR41a2	570	HHAM00008:711250..713291(-)	Hh.00g065170	yes	full
HhamIR60a	635	HHAM00052:309424..311829(-)	Hh.00g032900	yes	full
HhamIR68a	704	HHAM00503:15386..20473(+)	Hh.00g088380	yes	full
HhamIR75a	632	HHAM00040:48467..50801(-)	Hh.00g135170	yes	full
HhamIR75b	513	HHAM00009:227251..229050(-)	Hh.00g150250	yes	5' missing
HhamIR75c	397	HHAM04234:389..2053(-)	Hh.00g186250	yes	5' missing
HhamIR75d	580	HHAM00019:1043113..1051235(-)	Hh.00g031530, Hh.00g031540	yes	full
HhamIR75e	659	HHAM00018:653215..658301(-)	Hh.00g119030	yes	full
HhamIR75f	311	HHAM00059:440114..441318(-)	n.a.	yes	5' missing
HhamIR75g	566	HHAM00002:3032240..3034503(-)	Hh.00g008120	yes	full
HhamIR75h	837	HHAM00003:1961280..1981555(-)	Hh.00g055070	yes	full
HhamIR75i	420	HHAM00059:436474..437955(-)	n.a.	yes	5' missing
HhamIR76b	553	HHAM00165:162811..171401(-)	Hh.00g024430	yes	full
HhamIR93a	836	HHAM00114:240752..243937(+)	Hh.00g075320	yes	full
HhamIR100a	661	HHAM00026:754997..756979(-)	n.a.	yes	full

HhamIR100b	633	HHAM00036:481124..483022(+)	Hh.00g057180	yes	full
HhamIR101	575	HHAM00003:1932892..1934669(-)	Hh.00g055040	yes	full
HhamIR102	588	HHAM00003:1930125..1931993(-)	Hh.00g055020	yes	full
HhamIR103	555	HHAM05592:2..1621(-)	Hh.00g136210, Hh.00g136220	yes	full
HhamIR104	527	HHAM00029:423349..425120(+)	Hh.00g126650	no	full
HhamIR105	520	HHAM00044:343118..344677(+)	Hh.00g134470	yes	full
HhamIR106	565	HHAM00029:207075..208831(+)	Hh.00g126480	yes	full
HhamIR107	539	HHAM00012:352796..354415(-)	Hh.00g179480	no	full
HhamIR108	534	HHAM00012:354880..356484(+)	Hh.00g179490	no	full
HhamIR109	512	HHAM00012:356672..358210(+)	Hh.00g179500	no	3' missing
HhamIR110	514	HHAM00012:62069..63719(+)	Hh.00g178920	no	full
HhamIR111	560	HHAM00057:44620..46349(-)	Hh.00g129930	no	full
HhamIR112	541	HHAM00012:59389..63722(+)	Hh.00g178920	yes	full

n.a: no available

Supplementary Table S5: Candidate odorant-binding protein (OBP) genes.

Name	Length (aa)	Genomic position [scaffold:start..end(strand)]	Associated locus	Transcript evidence	Status
HhamOBP1	144	HHAM00009:177271..178364(+)	Hh.00g150150	yes	full
HhamOBP2	244	HHAM05218:1271..2107(-)	Hh.00g133250	yes	full
HhamOBP3	149	HHAM00035:36850..38409(-)	Hh.00g060810	yes	full
HhamOBP4	145	HHAM00035:19736..21140(-)	Hh.00g060790	yes	full
HhamOBP5	131	HHAM04151:1566..2013(-)	Hh.00g125310	yes	full
HhamOBP6	135	HHAM00048:199550..200439(+)	Hh.00g141660	yes	full

HhamOBP7	139	HHAM00008:764008..764479(-)	Hh.00g065240	yes	full
HhamOBP8	135	HHAM00147:195236..195639(+)	Hh.00g124760	yes	full
HhamOBP9	144	HHAM00005:581870..582326(+)	Hh.00g104530	yes	full
HhamOBP10	135	HHAM00001:1102090..1102606(+)	Hh.00g167790	yes	full
HhamOBP11	152	HHAM00008:749558..750064(-)	Hh.00g065200	yes	full
HhamOBP12	140	HHAM00012:730159..730860(+)	Hh.00g180060	yes	full
HhamOBP13	137	HHAM00008:748242..748713(+)	Hh.00g065190	yes	full
HhamOBP14	138	HHAM00008:783942..784660(+)	Hh.00g065270	yes	full
HhamOBP15	151	HHAM00008:794717..795194(+)	Hh.00g065300	yes	full
HhamOBP16	129	HHAM00008:765341..765682(-)	Hh.00g065250	yes	full
HhamOBP17	144	HHAM00186:145479..146123(-)	Hh.00g048190	yes	full
HhamOBP18	186	HHAM00003:1042921..1043881(+)	Hh.00g052920	yes	full
HhamOBP19	131	HHAM00008:727379..727826(-)	n.a.	yes	full
HhamOBP20	134	HHAM00011:1136913..1137367(-)	Hh.00g022150	yes	full
HhamOBP21	134	HHAM00008:781984..782385(+)	Hh.00g065260	yes	full
HhamOBP22	161	HHAM00008:762537..763043(-)	Hh.00g065220	yes	full
HhamOBP23	136	HHAM00008:763356..763718(-)	Hh.00g065230	yes	full
HhamOBP24	134	HHAM00008:792340..792723(+)	Hh.00g065290	yes	full
HhamOBP25	137	HHAM00011:1292848..1293319(+)	Hh.00g023630	yes	full
HhamOBP26a	151	HHAM00006:1129081..1129588(-)	Hh.00g099880	yes	full
HhamOBP26b	137	HHAM00006:1131466..1131830(-)	Hh.00g099880<br>Hh.00g099890	yes	full
HhamOBP27	134	HHAM00008:786229..786585(+)	Hh.00g065280	yes	full
HhamOBP28	177	HHAM00178:61429..63025(-)	Hh.00g063200<br>Hh.00g063210	yes	full

Supplementary Table S6: Chromosome Quotient (CQ) for *H. hampei* genome scaffolds.

Scaffold	Length	Female reads	Male reads	CQ
HHAM02931	4064	42	87073	0
HHAM03795	3138	6	3444	0.001
HHAM03825	3121	7	4522	0.001
HHAM05566	2239	5	7712	0.001
HHAM08091	563	1	1355	0.001
HHAM08139	541	1	1137	0.001
HHAM02100	5822	10	5407	0.002
HHAM04308	2819	3	1565	0.002
HHAM05516	2262	1	531	0.002
HHAM03007	3944	315	90156	0.003
HHAM05512	2263	156	29266	0.004
HHAM01994	6087	6	1104	0.005
HHAM03339	3521	18	2899	0.005
HHAM07706	714	2	284	0.006
HHAM06836	1317	15	1518	0.008
HHAM07082	1113	12	1313	0.008
HHAM02179	5629	16	1089	0.012
HHAM02421	5033	28	1815	0.013
HHAM02018	6037	18	1058	0.014
HHAM07010	1176	12	622	0.016
HHAM05846	2137	948	34121	0.023
HHAM07845	660	406	14143	0.024
HHAM06723	1410	72	2247	0.027
HHAM06511	1654	98	2541	0.032
HHAM04328	2807	174	4130	0.035
HHAM04470	2741	153	3266	0.039

HHAM01897	6360	174	3224	0.045
HHAM06851	1305	98	1056	0.077
HHAM03698	3204	46	461	0.083
HHAM03638	3245	132	1227	0.09
HHAM00577	25934	6930	42866	0.135
HHAM07040	1142	21	116	0.151
HHAM03620	3264	686	3432	0.167
HHAM08122	549	56	278	0.168
HHAM07433	864	123	522	0.196
HHAM06939	1229	717	3006	0.199

Supplementary Table S7: RepeatMarker analysis for male-specific *H. hampei* scaffolds.

pValue	Score	Method	SeqID	Begin	End	Repeat	Type
-	14	RMasker/TRF	HHAM00577	870	922	+ A-rich	Low_complexity
6.80E-75	656	WUBlastX	HHAM00577	1885	2904	+ Mariner-19_LMi_	DNA/TcMar-Tc1
-	15	RMasker/TRF	HHAM00577	2906	2940	+ (TTATT)n	Simple_repeat
1.00E-28	31	WUBlastX	HHAM00577	3527	3958	- Sola1-1_AP_tp	DNA/Sola-1
1.30E-67	122	WUBlastX	HHAM00577	3608	3967	- Sola1-9_LMi_tp	DNA/Sola-1
1.60E-36	252	WUBlastX	HHAM00577	3952	4341	- Sola1-9_LMi_tp	DNA/Sola-1
1.30E-67	453	WUBlastX	HHAM00577	4188	5198	- Sola1-9_LMi_tp	DNA/Sola-1
-	13	RMasker/TRF	HHAM00577	6839	6868	+ (CGACA)n	Simple_repeat
2.90E-01	34	WUBlastX	HHAM00577	7232	7504	- HARB-3_STu_tp	DNA/PIF-Harbing
3.20E-21	118	WUBlastX	HHAM00577	7289	7516	- Harbinger-5_XT_	DNA/PIF-Harbing
3.00E-05	114	WUBlastX	HHAM00577	7533	7769	- Harbinger3_DR_t	DNA/PIF-Harbing
4.80E-04	53	WUBlastX	HHAM00577	7563	7829	- MtPH-M-3-Ia_tp	DNA/PIF-Harbing
1.50E-21	133	WUBlastX	HHAM00577	7575	7769	- Harbinger-1_XT_	DNA/PIF-Harbing
2.90E-01	29	WUBlastX	HHAM00577	7635	7868	- HARB-3_STu_tp	DNA/PIF-Harbing
-	13	RMasker/TRF	HHAM00577	7780	7819	+ (AATGT)n	Simple_repeat
1.90E-20	96	WUBlastX	HHAM00577	7844	8023	- Harbinger-1_NV_	DNA/PIF-Harbing
8.20E-22	23	WUBlastX	HHAM00577	8046	8126	- Harbinger2_DR_t	DNA/PIF-Harbing
-	14	RMasker/TRF	HHAM00577	8365	8382	+ (ATC)n	Simple_repeat

1.40E-67	658	WUBlastX	HHAM00577	8885	9643	- Mariner-13_HSal	DNA/TcMar-Marin
8.60E-08	22	WUBlastX	HHAM00577	19490	19537	- CR1-12_NV	LINE/Rex-Babar
-	16	RMasker/TRF	HHAM00577	19669	19688	+ (T)n	Simple_repeat
1.00E-156	1127	WUBlastX	HHAM00577	19931	21994	- SARTTc5_pol	LINE/R1
2.90E-30	66	WUBlastX	HHAM00577	21728	22072	- I_Ele16_pol	LINE/I
2.30E-33	48	WUBlastX	HHAM00577	21951	23006	- I-72_AAe_pol	LINE/I
1.00E-156	322	WUBlastX	HHAM00577	21975	22832	- SARTTc5_pol	LINE/R1
5.20E-02	36	WUBlastX	HHAM00577	23276	23659	- I-3_AC_gag	LINE/I
8.30E-01	46	WUBlastX	HHAM00577	23318	23587	- L1-5_SBi_gag	LINE/L1
4.20E-23	162	WUBlastX	HHAM00577	23393	24934	- SARTTc2_gag	LINE/R1
1.30E-18	176	WUBlastX	HHAM00577	23447	23731	- UN-22004035_Fsc	LINE/R1
-	12	RMasker/TRF	HHAM01897	583	622	+ GA-rich	Low_complexity
1.10E-33	186	WUBlastX	HHAM01897	1192	3138	+ SARTTc2_gag	LINE/R1
-	14	RMasker/TRF	HHAM01897	2342	2383	+ (AGGAAT)n	Simple_repeat
9.60E-21	204	WUBlastX	HHAM01897	2386	3084	+ SART-4_MOc_gag	LINE/R1
5.80E-172	1582	WUBlastX	HHAM01897	3179	6094	+ SARTTc5_pol	LINE/R1
1.10E-19	24	WUBlastX	HHAM01897	5792	6154	+ Tad1-20_BG_pol	LINE/Tad1
2.80E-05	30	WUBlastX	HHAM01897	5969	6223	+ UN-L1_Mdo3_pol	LINE/L1
4.20E-09	133	WUBlastX	HHAM01994	2	274	+ SART-4_APi_pol	LINE/R1
7.50E-05	91	WUBlastX	HHAM01994	2	436	+ R1-1_SIn_pol	LINE/R1
2.90E-03	91	WUBlastX	HHAM01994	5	352	+ SART-1_MOc_pol	LINE/R1
-	15	RMasker/TRF	HHAM01994	503	515	+ A-rich	Low_complexity
-	36	RMasker/TRF	HHAM01994	516	549	+ (AGGTT)n	Simple_repeat
-	15	RMasker/TRF	HHAM01994	550	564	+ A-rich	Low_complexity
-	17	RMasker/TRF	HHAM01994	725	758	+ (T)n	Simple_repeat
7.00E-18	33	WUBlastX	HHAM01994	2517	2966	+ UN-22004035_Fsc	LINE/R1
1.00E-165	1062	WUBlastX	HHAM01994	2966	5908	+ SARTTc5_pol	LINE/R1
-	16	RMasker/TRF	HHAM01994	3702	3747	+ (ATTAT)n	Simple_repeat
-	13	RMasker/TRF	HHAM01994	3761	3790	+ GA-rich	Low_complexity
-	12	RMasker/TRF	HHAM01994	4554	4588	+ A-rich	Low_complexity
-	17	RMasker/TRF	HHAM01994	4903	4948	+ (AATATAA)n	Simple_repeat

-	13	RMasker/TRF	HHAM01994	5305	5344	+ (ATTAA)n	Simple_repeat
7.20E-04	20	WUBlastX	HHAM01994	5829	5927	+ ORTE-5_AAe_pol	LINE/RTE-ORTE
3.00E-151	296	WUBlastX	HHAM02018	226	801	- SARTTc3_pol	LINE/R1
8.90E-83	321	WUBlastX	HHAM02018	572	1450	- Waldo-6_AAe_pol	LINE/R1
3.00E-151	408	WUBlastX	HHAM02018	794	1456	- SARTTc5_pol	LINE/R1
7.90E-98	366	WUBlastX	HHAM02018	818	1600	- R1-2B_DK_pol	LINE/R1
5.20E-11	66	WUBlastX	HHAM02018	1285	2214	- CR1-47_AAe_pol	LINE/CR1
6.20E-21	128	WUBlastX	HHAM02018	1345	1983	- CR1-108_AAe_pol	LINE/CR1
3.00E-151	756	WUBlastX	HHAM02018	1453	3141	- SARTTc5_pol	LINE/R1
2.90E-22	24	WUBlastX	HHAM02018	2965	3207	- Crack-24_AAe_po	LINE/L2
9.40E-18	31	WUBlastX	HHAM02018	3040	3171	- UN-I-1_AF_pol	LINE/Tad1
1.80E-02	42	WUBlastX	HHAM02018	3576	4142	- I-61_AAe_gag	LINE/I
1.40E-01	30	WUBlastX	HHAM02018	3576	4355	- Jockey-2_HMM_ga	LINE/I-Jockey
1.10E-128	93	WUBlastX	HHAM02018	3633	4049	- R1-1B_DK_pol	LINE/R1
2.10E-39	352	WUBlastX	HHAM02018	3702	5030	- SARTBm3_gag	LINE/R1
-	16	RMasker/TRF	HHAM02018	5058	5148	+ (TTTTAGA)n	Simple_repeat
1.10E-34	130	WUBlastX	HHAM02018	5186	5596	- SARTTc2_gag	LINE/R1
-	18	RMasker/TRF	HHAM02018	5965	5986	+ (T)n	Simple_repeat
2.30E-11	35	WUBlastX	HHAM02100	527	757	+ SARTTc2_gag	LINE/R1
9.30E-28	60	WUBlastX	HHAM02100	701	1429	+ SARTTc1_gag	LINE/R1
3.60E-25	235	WUBlastX	HHAM02100	1462	2169	+ SARTTc3_gag	LINE/R1
1.50E-14	137	WUBlastX	HHAM02100	1477	2526	+ SART-4_MOc_gag	LINE/R1
-	11	RMasker/TRF	HHAM02100	1538	1576	+ A-rich	Low_complexity
-	12	RMasker/TRF	HHAM02100	2558	2594	+ (CTTTTA)n	Simple_repeat
3.00E-133	84	WUBlastX	HHAM02100	2720	2920	+ Waldo-1_AAe_pol	LINE/R1
1.70E-126	94	WUBlastX	HHAM02100	2753	3001	+ R1-1_DEl_pol	LINE/R1
7.60E-122	100	WUBlastX	HHAM02100	2753	2941	+ LINER1-2_NVi_po	LINE/R1
3.00E-35	139	WUBlastX	HHAM02100	2872	3414	+ R1-5_BM_pol	LINE/R1
1.50E-179	1538	WUBlastX	HHAM02100	2971	5631	+ SARTTc7_pol	LINE/R1
2.20E-156	1470	WUBlastX	HHAM02179	182	3076	- SARTTc5_pol	LINE/R1
4.40E-26	20	WUBlastX	HHAM02179	2975	3097	- Loa-1_RPr_pol	LINE/R1-LOA

6.70E-02	55	WUBlastX	HHAM02179	3246	3902	- Tx1-19b_Lch_gag	LINE/L1-Tx1
6.90E-28	294	WUBlastX	HHAM02179	3387	4274	- SARTTc1_gag	LINE/R1
6.50E-41	327	WUBlastX	HHAM02179	3531	5198	- SARTBm3_gag	LINE/R1
8.20E-21	20	WUBlastX	HHAM02179	5481	5624	- SARTTc2_gag	LINE/R1
4.90E-136	1221	WUBlastX	HHAM02421	1	2199	- SARTTc7_pol	LINE/R1
-	12	RMasker/TRF	HHAM02421	1218	1262	+ (TTTCTA)n	Simple_repeat
8.60E-04	26	WUBlastX	HHAM02421	2083	2223	- UN-CgT1_pol	LINE/Tad1
8.30E-20	21	WUBlastX	HHAM02421	2328	2399	- BS2_pol	LINE/I-Jockey
3.10E-03	50	WUBlastX	HHAM02421	2388	2804	- Nimb-11_LMi_gag	LINE/I
2.70E-01	29	WUBlastX	HHAM02421	2433	3167	- I_Ele42_gag	LINE/I
5.70E-55	484	WUBlastX	HHAM02421	2550	3962	- SARTTc1_gag	LINE/R1
4.10E-22	70	WUBlastX	HHAM02421	3570	4256	- SARTTc2_gag	LINE/R1
-	12	RMasker/TRF	HHAM02421	3849	3880	+ (CTCCTT)n	Simple_repeat
-	49	RMasker/TRF	HHAM02421	4975	5019	+ (AACCT)n	Simple_repeat
-	19	RMasker/TRF	HHAM02931	2429	2467	+ (ATAATT)n	Simple_repeat
-	13	RMasker/TRF	HHAM02931	2757	2808	+ (TAATAT)n	Simple_repeat
1.50E-08	116	WUBlastX	HHAM03339	10	405	+ Waldo-1_AAe_gag	LINE/R1
1.30E-18	178	WUBlastX	HHAM03339	70	387	+ UN-22004035_Fsc	LINE/R1
-	13	RMasker/TRF	HHAM03339	430	473	+ A-rich	Low_complexity
2.20E-67	239	WUBlastX	HHAM03339	485	1096	+ R1-5_BM_pol	LINE/R1
1.10E-163	1548	WUBlastX	HHAM03339	581	3430	+ SARTTc3_pol	LINE/R1
-	12	RMasker/TRF	HHAM03620	3	31	+ (AATA)n	Simple_repeat
-	12	RMasker/TRF	HHAM03620	119	147	+ A-rich	Low_complexity
-	14	RMasker/TRF	HHAM03620	642	690	+ (AATTA)n	Simple_repeat
-	18	RMasker/TRF	HHAM03620	980	1001	+ (T)n	Simple_repeat
5.00E-95	891	WUBlastX	HHAM03620	1990	3261	+ Mariner3-1_HSal	DNA/TcMar-Tc4
-	68	RMasker/TRF	HHAM03638	1	61	+ (AGGTT)n	Simple_repeat
7.40E-124	207	WUBlastX	HHAM03638	65	652	+ SART-1_CF_pol	LINE/R1
8.10E-65	179	WUBlastX	HHAM03638	65	772	+ R1-5_BM_pol	LINE/R1
6.10E-184	1650	WUBlastX	HHAM03638	188	2989	+ SARTTc1_pol	LINE/R1
-	12	RMasker/TRF	HHAM03638	3101	3119	+ (AAC)n	Simple_repeat

1.40E-03	20	WUBlastX	HHAM03698	295	525	+ Waldo-1_AAe_gag	LINE/R1
2.00E-16	29	WUBlastX	HHAM03698	334	588	+ R1-7_DPer_gag	LINE/R1
5.50E-22	74	WUBlastX	HHAM03698	382	861	+ SARTTc2_gag	LINE/R1
-	11	RMasker/TRF	HHAM03698	1419	1462	+ A-rich	Low_complexity
7.50E-11	90	WUBlastX	HHAM03698	1483	2010	+ LINER1-3_NVi_ga	LINE/R1
4.20E-27	61	WUBlastX	HHAM03698	2031	2219	+ SARTBm3_gag	LINE/R1
-	38	RMasker/TRF	HHAM03698	2516	2623	+ A-rich	Low_complexity
-	13	RMasker/TRF	HHAM03698	2750	2778	+ A-rich	Low_complexity
1.30E-07	134	WUBlastX	HHAM03698	2783	3187	+ SARTTc7_pol	LINE/R1
5.80E-07	68	WUBlastX	HHAM03825	893	1492	+ REP-7_CCri_1p	Unknown/Helitro
6.80E-08	84	WUBlastX	HHAM03825	1061	1522	+ REP-5_CCri_1p	Unknown/Helitro
3.70E-154	1360	WUBlastX	HHAM04308	432	2780	- SARTTc7_pol	LINE/R1
-	41	RMasker/TRF	HHAM04308	2782	2819	+ (CCTAA)n	Simple_repeat
9.80E-11	25	WUBlastX	HHAM04328	103	315	+ L1-29_ACar_pol	LINE/L1
1.10E-12	20	WUBlastX	HHAM04328	106	402	+ RTE-7_Hmel_pol	LINE/RTE-RTE
7.70E-138	1277	WUBlastX	HHAM04328	160	2070	+ SARTTc7_pol	LINE/R1
-	13	RMasker/TRF	HHAM04470	2079	2132	+ A-rich	Low_complexity
-	28	RMasker/TRF	HHAM05512	578	640	+ (TAA)n	Simple_repeat
-	12	RMasker/TRF	HHAM05516	98	140	+ (TAAGAA)n	Simple_repeat
2.10E-184	887	WUBlastX	HHAM05516	148	975	- Mariner-7_LHu_t	DNA/TcMar-Tc4
1.30E-32	206	WUBlastX	HHAM05516	151	1689	- Mariner-15_AEc_	DNA/TcMar-Tc4
1.10E-33	214	WUBlastX	HHAM05516	163	1413	- Mariner3-1_HSal	DNA/TcMar-Tc4
3.40E-57	420	WUBlastX	HHAM05516	691	1605	- Mariner3-3_LMi_	DNA/TcMar-Tc4
2.10E-184	730	WUBlastX	HHAM05516	1054	1893	- Mariner-7_LHu_t	DNA/TcMar-Tc4
-	12	RMasker/TRF	HHAM05516	2211	2254	+ (TTTTTA)n	Simple_repeat
-	11	RMasker/TRF	HHAM05566	1143	1197	+ (TTTATTA)n	Simple_repeat
3.90E-18	162	WUBlastX	HHAM05846	1183	2058	+ Mariner-25_SIn_	DNA/TcMar-Marin
2.90E-30	171	WUBlastX	HHAM05846	1663	2058	+ Mariner-21_AEc_	DNA/TcMar-Marin
-	26	RMasker/TRF	HHAM06511	1	25	+ (AGGTT)n	Simple_repeat
-	51	RMasker/TRF	HHAM06851	1251	1305	+ (A)n	Simple_repeat
-	13	RMasker/TRF	HHAM06939	753	785	+ (TTCATT)n	Simple_repeat

-	19	RMasker/TRF	HHAM07040	413	473	+ A-rich	Low_complexity
-	18	RMasker/TRF	HHAM07040	1031	1076	+ A-rich	Low_complexity
-	12	RMasker/TRF	HHAM07082	394	422	+ (AATA)n	Simple_repeat
-	12	RMasker/TRF	HHAM07082	510	538	+ A-rich	Low_complexity
-	14	RMasker/TRF	HHAM07082	1033	1081	+ (AATTA)n	Simple_repeat
-	18	RMasker/TRF	HHAM07433	144	165	+ (T)n	Simple_repeat
2.40E-24	256	WUBlastX	HHAM07433	643	864	- Mariner3-3_HSal	DNA/TcMar-Tc4
1.00E-11	175	WUBlastX	HHAM08122	2	235	+ Gypsy-39_DPu_po	LTR/Gypsy
1.10E-04	107	WUBlastX	HHAM08122	2	334	+ Gypsy-16_DPu_po	LTR/Gypsy
2.00E-03	90	WUBlastX	HHAM08122	2	406	+ Gypsy-21_DEl_po	LTR/Gypsy
1.40E-04	107	WUBlastX	HHAM08122	38	277	+ Gypsy-59D_CCri_	LTR/Gypsy
-	28	RMasker/TRF	HHAM08139	414	459	+ (A)n	Simple_repeat
-	48	RMasker/TRF	HHAM08139	470	518	+ (TAGGTTAGGA)n	Simple_repeat

Supplementary Table S8: Primer sequences used in this study.

Sequence target	Forward	Reverse	Product size
Male-specific scaffold primers			
HHAM03825	ACGTCGCTAGAAACAACGGT	TTCCTTCAATGGCACGGTGA	763
HHAM03795	GGCTTTGGGGCTGCATATTG	TTAACTGCGCCGAAGCTGTA	790
HHAM03698	ACGGTACAAGTAATGGGGCG	AGGTTTGGGTCATCTGGCTC	970
HHAM02018	TGTTTCACAAGACGGGCTGT	ATACCCTAGTGCAGGTCGGT	463
HHAM03007	CAACGAGGACCCTCAACAGG	GGGTGTAGTCACCTTCGTGG	663
HHAM02931	AATTGGGCGAGGGATCGTTT	ACATGGGGTACCTGCCAATG	732
HHAM03339	CGAGTCACGGAGTTGTTCCA	TCGGTGGCATATAGCAGCAG	617
Actin	AGCCAACAGAGAAAAGATG	ACCAGCCAAATCCAAAC	208
Male-specific candidate gene RT-PCR primers			
Hh.00g036580	GACAGCCAAAGGAGAGTTGC	CAGCCCCCTCCAAGGTATTC	188
Hh.00g048560	CTCAAGGCATTTGCAGTGGC	GTGCCATCCAACATGGGGTA	189
Hh.00g129860	CGTCCACTACGATGGGCATT	AGTTTGCACCTCCCACACTC	184
Hh.00g144270	TGGAGTTTCGACCCACATGG	TTCCTGACGAATGGGAGCAG	179

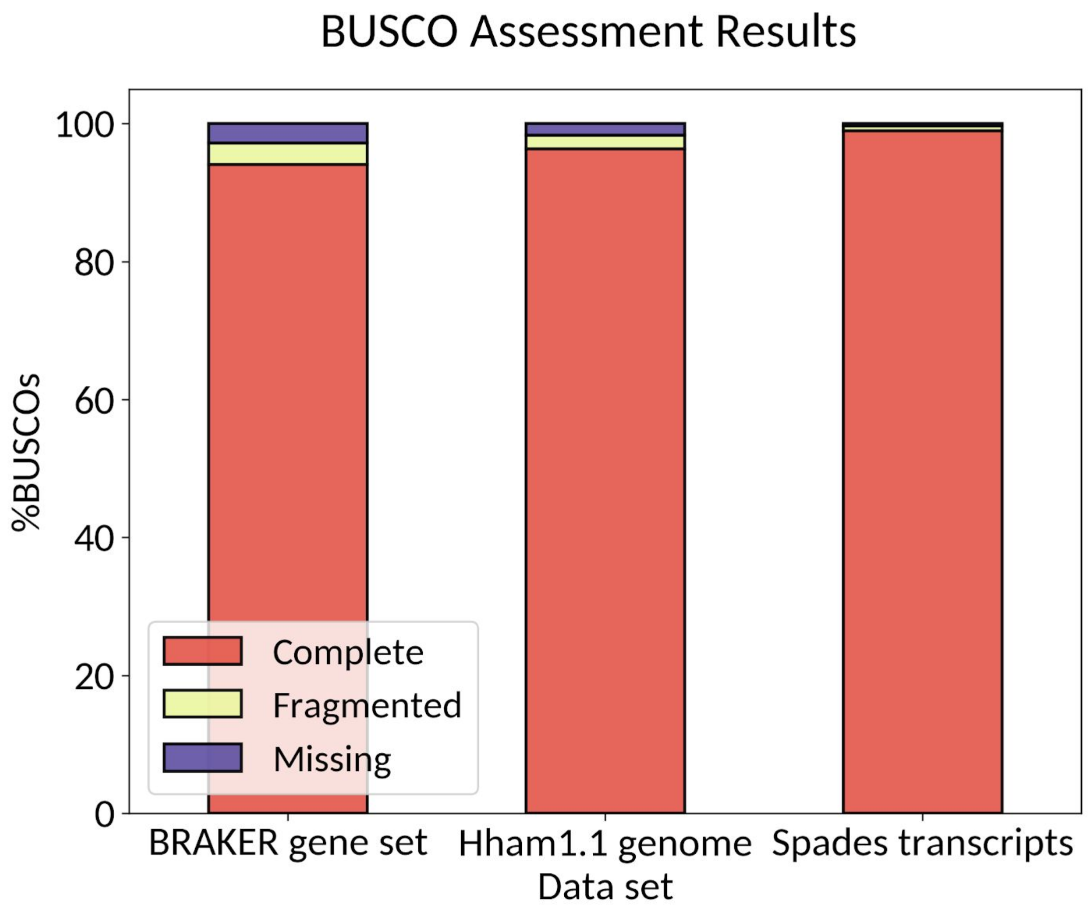

Supplementary Figure S1. Genome completeness. Benchmarking Universal Single-Copy Orthologs (BUSCO) assessment for completeness of the (A) *H. hampei* PB1 genome assembly, (B) EvidenceModeler gene prediction and (C) Evigen-CBB transcriptome assembly.

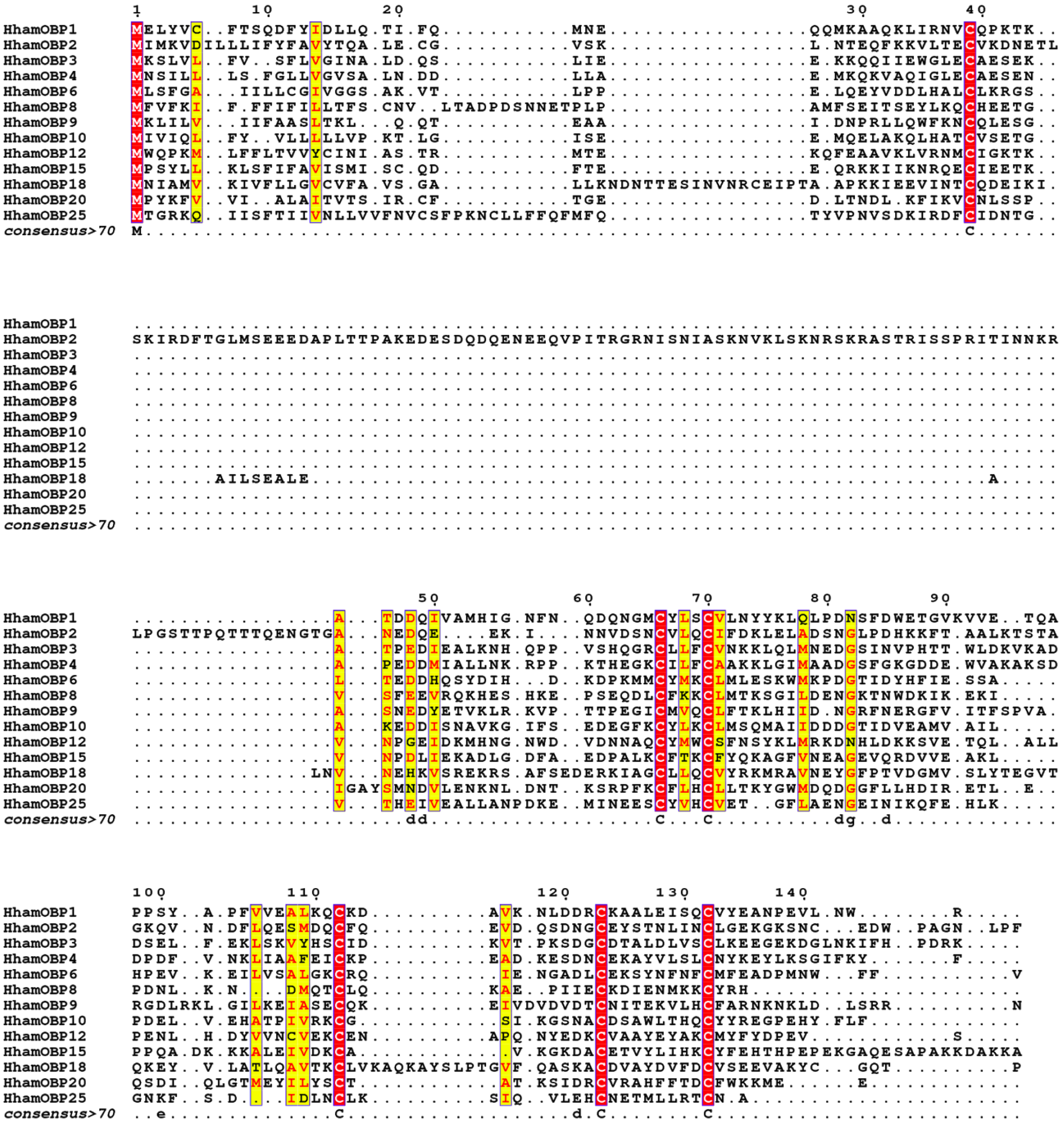

Supplementary Figure S2: HhamOBP members of the Classical OBP family. Protein alignment was performed with T-Coffee (http://tcoffee.crg.cat). Conserved 6C (Cystein) pattern are highlighted in red boxes (except the first methionine [M]).

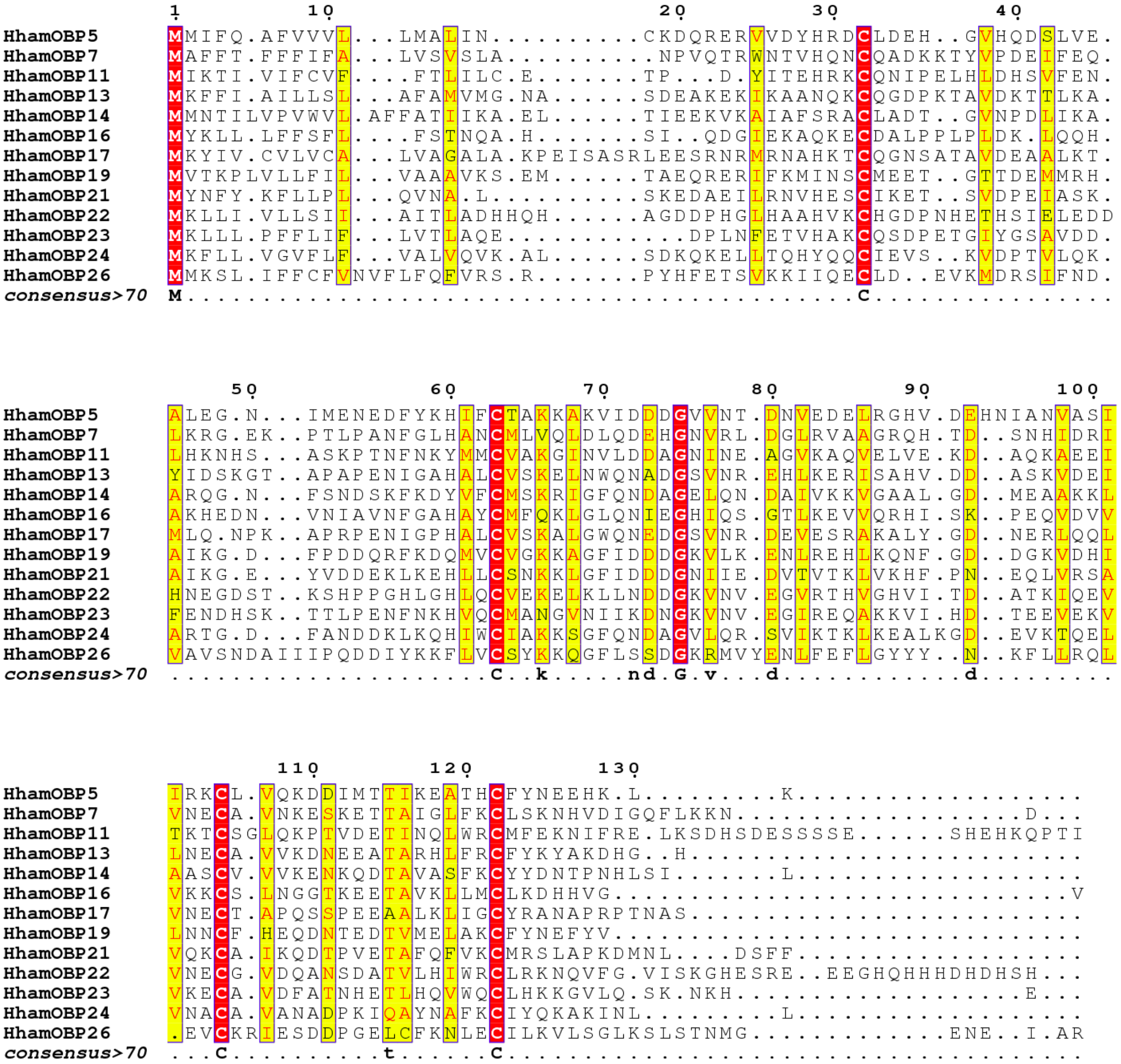

Supplementary Figure S3: HhamOBP members of the Minus-C OBP family. Protein alignment was performed with T-Coffee (http://tcoffee.crg.cat). Conserved 4C (Cystein) pattern are highlighted in red boxes (except the conserved methionine [M] and glycine [G]).

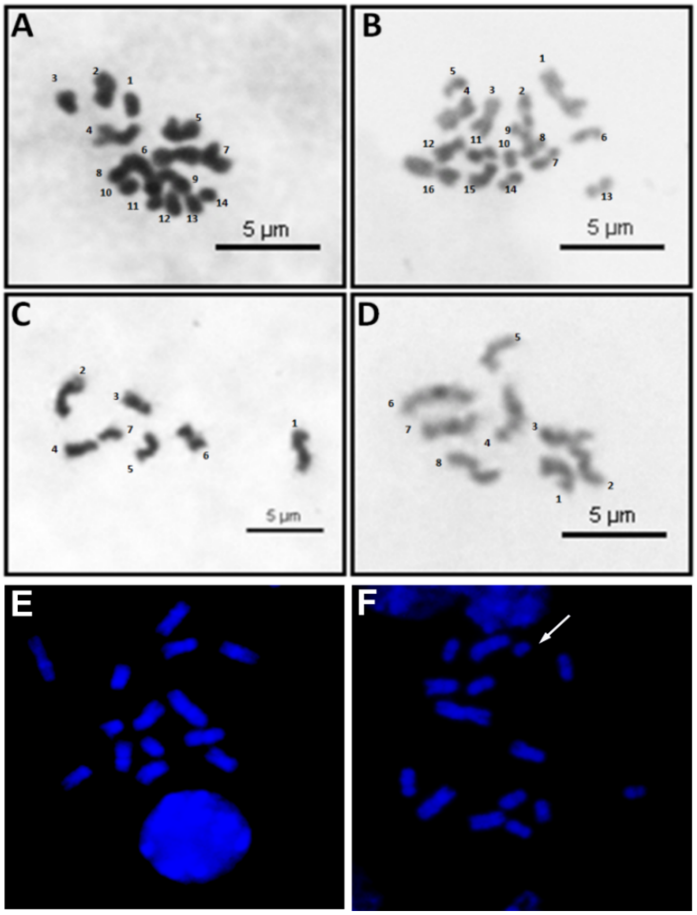

Supplementary Figure S4. Cytological analysis in the coffee berry borer. Meiotic metaphase I from male gonad cells containing the normal 7 diploid chromosomes (2n=14) (A) or containing an extra chromosome (14+1) (B). Meiotic metaphase II from male gonad cells with the normal haploid chromosome number (n=7) (C) or containing the extra chromosome (7+1) (D). Metaphase preparations (DAPI staining) from CBB embryos showing cells with 14 chromosomes (E) and cells with 15 chromosomes (F). Arrow in F denotes the extra chromosome.

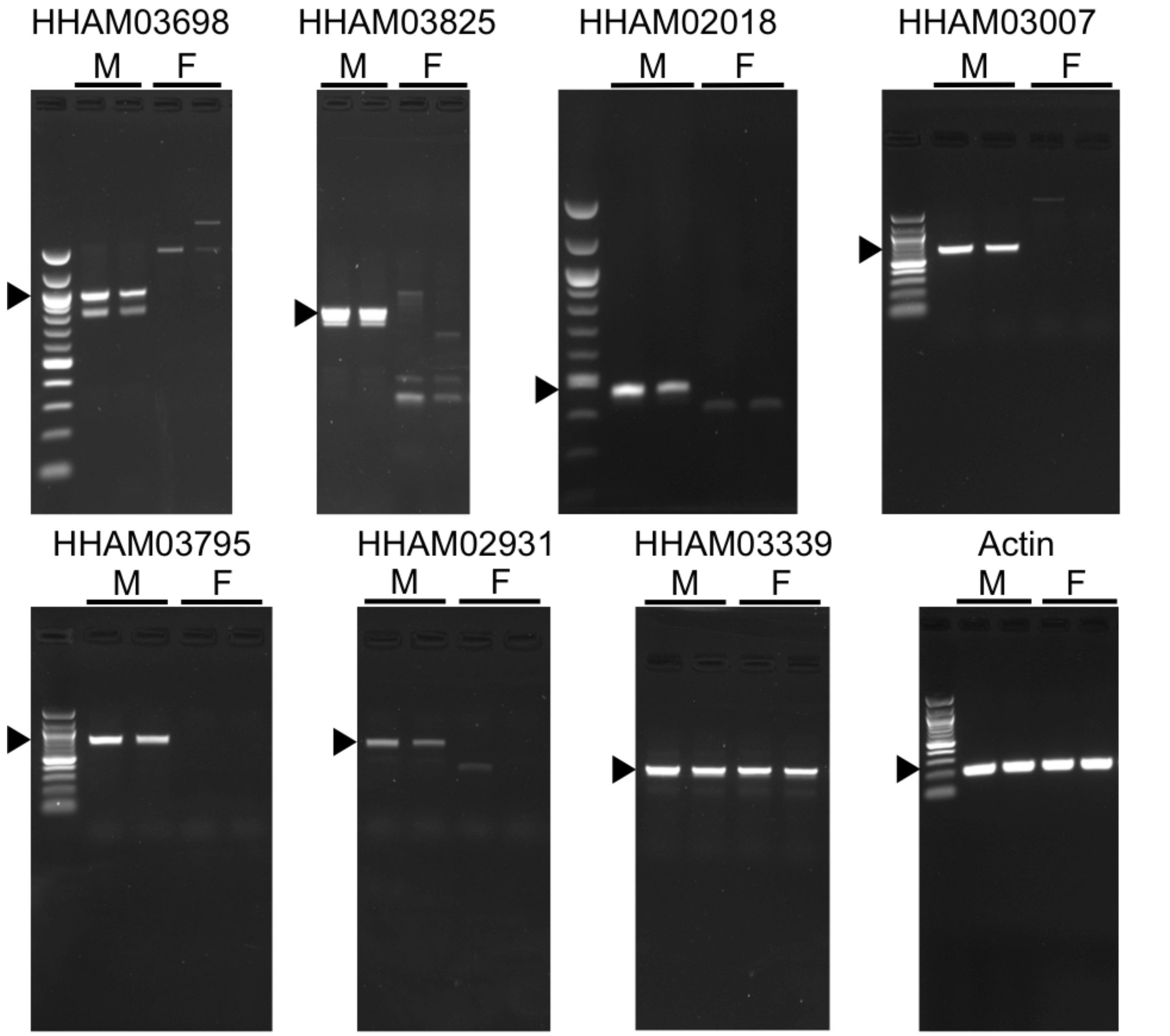

Supplementary Figure S5. Full-length gel electrophoresis of PCR detection for DNA markers associated to candidate male-specific scaffolds HHAM03698, HHAM03825, HHAM022018, HHAM03007, HHAM03795, HHAM02931 and HHAM03339. Genomic DNA samples from adult males (M) and females (F) of *Hypothenemus hampei* were used as template for PCR using primers in Supplementary Table S4. Black arrows (▶) indicate the expected DNA band size for each marker.

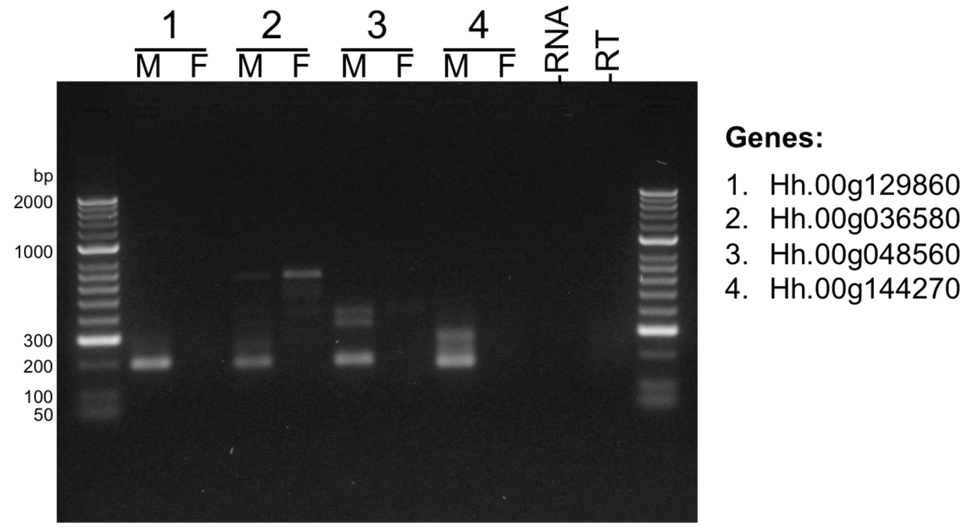

Supplementary Figure S6. Full-length electrophoresis gel image for RT-PCR analysis of candidate Y-linked genes using total RNA from males (M) and females (F). Control RT-PCR assays for gDNA contamination were performed using primers for Hh00g129860, with no male-RNA template (-RNA) or lacking retro-transcriptase enzyme (-RT).

Supplementary File S1. Protein sequences for chemosensory genes from *Hypothenemus hampei*

A coffee berry borer (*Hypothenemus hampei*) genome assembly reveals a reduced chemosensory receptor gene repertoire and male-specific genome sequences

Lucio Navarro[1,*], Erick M. Hernandez-Hernandez[2], Jonathan Nuñez[3], Flor E. Acevedo[4], Alejandro Berrio[5], Luis M. Constantino[1], Beatriz E. Padilla-Hurtado[6], Diana Molina[1], Carmenza Gongora[1], Ricardo Acuña[1], Jeff Stuart[7], Pablo Benavides[1].

[1]National Coffee Research Center - CENICAFE, Manizales, Colombia.
[2]UNESP-Univ. Estadual Paulista, São José do Rio Preto, SP, Brazil.
[3]Manaaki Whenua – Landcare Research, Lincoln 7640, New Zealand.
[4]Department of Entomology, Pennsylvania State University, University Park, PA, USA.
[5]Department of Biology, Duke University, Durham, USA.
[6]Instituto de Investigación en Microbiología y Biotecnología Agroindustrial. Universidad Católica de Manizales, Manizales, Colombia.
[7]Department of Entomology, Purdue University, West Lafayette, USA.

```
>HhamOrCo
MINKFKVAGLVADLMPNIRLIQASGHFMFNYYADDTGSLHLLRVGYSCMHLFLVLVQYGC
IFGNLIKEKDNVNYLAANTVTILFFTHCITKFVYFALQSKMFYRTLGIWNQSNSHPIFQE
SNNRYHALALKKMRTLLYIILIGSIFSVCAWTGITFVEDSVHLVKDPDNENGTIVEAIPK
LLIKSWYPFNAMSGIPYYIALVYQIYYVTFSLLHANLLDSLFCSWLIFACEQLQHLKEIL
KPLMELSAALDTYVPKSADLFKSSGSATSHDNLIENDYNAKNDELKGIYSTSQELGNLHF
RSGALQTFGQGGGGVGPNGLTKKQELMVRSAIKYWVERHKHVVRLVTAIGDAYGVALLFH
MLTATIMLTLLAYEATKIDGINTYAATTLGYLIYSLAQVFHFCIFGNRLIEESSSVMEAA
YSCHWYDGSEEAKTFVQIVCQQCQKALSISGAKFFTISLDLFASVLGATVTYFMVLVQLK
>HhamOR1
MSVSLYKVAKFFMLPTGIWKLPISNNPHVQTAYSIYSVFVLLLYISYTLSMVIRFIELCY
ELEISQLYVFLTLTILILEINYKILIYLKNGIPQMFAVVINREEIVLKTKEKEILDVYLQ
QVRYYKFATFCQCFCSFSGITWFILVNLYTKYYVGVNEHFMYELWFPFDKEQHEKFVVFY
NILLAYYGFLFNCASQTPLQTLMVFSTSQLRILQVSLRKCCDLEEQPNQDRIKMIKKLIK
EHQFLIEFIKDLNAAIQNTIFLEFIVESVHGAGALLQIISVKSMIEIPYASMYLILLVIN
MSALAWSANEVIVQSTNIANAVYESNWMNQSKEIKQLLFFILERAQKPLSLRIGSFGPMN
AEAALMTAKGSYTYAQFVITNRARKK
>HhamOR2
MPPGEAPDYFDMHYQFSAPYLLIPPKNSILKTIFWVVAVPHFFLTCMTTFLEYTKLFVGG
SGNFSADILNFGISTLHLMAVSRVSRWFFVKSHYDKVLKQVREIHTELYYFDYDHVDDSS
SKPIKKYVEGQTLDTRRHCLKISVAFITFVWINIIISYVTNYFTFPEVERWNPHKNKTST
YRDYPYPLYYPFDISISDGYYWLGFFYQPYAFLFLMCAFLCIDCLTVNTIIHLTSFINIL
NFAISCVDRNLNKSLSFEGSLKITEKRILKCIKMSEKIYKCCKMLNDVCSSQLFFQQICL
STVMCCSVYRVTSRPSSSEMIYLSSIVFSVALEMFNVAWQNQNFTLKAFELLSNIYELNW
LSYSVKLRRVLLCFMMRIQKPFHFTMGLGFPLEIGVFLTMVKSSYSFYALITQSGNHLSY
DQN
>HhamOR3
MYALTKDKPFYCALLLLIVFLWYPPTNHRLYKKWFFGFSAALRTISLLTAMGTFAHLILA
IKNNADIDISEDIGDLTGFVGCMTICLNFLMYHSEWAKLFNQLSDFKHFGQPPEYEKVVK
QGNFTTLMCIIYTIPGMLSYCCMSYIDIPNCKKNNILKGLNEPCGMINPTWLPLEEMKGT
YFIMFYFLQAVGIFIYLPSSFVICTVPWEAVGVIVARINHLKGIFKDVFRQDDEDRCIGQ
```

LQYCIQYHQDIIKTSQELSFLVRRTVKNLFLLIAMIIGSLGSQVLKSSTPKAVTFVLGYI
MAMFFICHAGQRLIDESLDLTNQIYDSKWYDMKPKIKRDIAFVLARCQKPMTLTGPPSMI
CLGHVLFNTIIKTSYSYLTLLNEMV
>HhamOR4
MPFLYLGDNWKRLLYSVYSYTLVTTASIYVISEIIAIHEAFGDISMILALLAMLLTHMVG
MGKISMLIFLKGRIQNISNKLLDRQFEYAPLDDFQPGEKLQGEKSFLMLITLFLFGLYNF
VGVSAHISSALTLNRNTVDHNFASNTTCKSFIPYKFYFPFDVDTVNGCHYLLGYMDLCLD
ILAGYIADTIFLILLHLLATQLNILKEALITIRKRCEHKSKLRDMENPALETEMYNEMKR
CIKHLYSLIEVSTEMENTYSFITLGQVLASLLIMASCLFTAASVPIGSPIFFSQMEYFIA
VICQISMYCWFGNQITLAVSVRLVAKVTIFNFGWFLEQGLDIPNAIYSSDWYDSSPRFKH
SMVMTMIRMKRPLFVSIGKFTPLSLATLLSVIRGSFSYFTLFQKAKNQS
>HhamOR5
MARIWDEMVSLGSKFFWAISPRNIQMTNVKDKDYEFFPGGYIFGIEFFCYQRILYKLYWL
LLYLNLPLLFMELWCFVNGENPSMSLIVKHQFTIVVHVTGILFQLIIMQEREKIYKANET
AKKLQWSLNFLNSKVKRIVAMRSVAINICMTLSYFVIILYLYNIPKEIQKSLLMQKIVQL
NQNSLTYTVLGMKLLNIIFLNYFIILMPLVHIYFTLEGEFQVFLLAEYVSKLIFHLMRTG
GKANGKFVSEKLRKIVKRQNELKQFYLDNEKLVNTAGIKFLFIMSLYVAIIIYAIFEKAL
ETTTIISCILMAFLNTVCATVGGQRYEDQIRVYAELCKMPWYDWSNENKRFYQLILIGSS
KIPRVAIFQDTYLNYSLGLKVG
>HhamOR6
MVLFNFLKYAQYPLRISAVWPQNNPSCILSFRMMLTSLIIGSFVVGQLYNSALLLSSFVK
FSQSLYLLISYLNVCIKIIILHSKKGQFLKLIDRIENQYFTKYERVYQHFLINVDKMLNR
VNWIYWTMVMISYIFFGIFPLVDSSKPLPIDFPHFNDGPFHIPFYIYEMISLFAAAFCNT
ALDMLFVGLSTVALVQLIIILNNKLKDTNKNVKCLKFYCDENRQELTVEYIKECCEHYIEI
EVFLENIESIFSVTLFIQLGMSVFAICNGGLMLINVEIFSFQGVSVIFYILSMVVQLGVY
CWFGNNVYFESLEIIHSCSLSNWYEESHDVKKMLLILMERTKRPLEIKALNFTTINADTF
VAILKWSYSYFALLNNFTN
>HhamOR7
MDISKYFQLPRIMLILSGTWPQKSPFQKYFQKGYLLTEFLIVSLSVKACFSLIYDLNTFF
KKLVLVSSLVMTTVKGWVFLGNGKHVLELYEYLNDPHFINVPQHLEKHMIDTMDITKKTM
QWFLTVIFVLLCLINILPFLTDDGVMFIPITLFGHFKWFMMVMQILNVDDIGLKTFGLDM
LFVYATCVIKAQLCILSGKIRFIGSEYQDSENGKSAETSLTDCVVHHYKIARLTELTQTI
FSFVLFVQYIESVITVCTVGFQLLTTSLFSLDFLVMSLYCNAALFQLVVYCWFGNEIIVY
SADISNACYDCNWLNMSTTARNMLFLIMERSKRPFYLTAGNFFTLSLQSLTSVLKSAYSI
VALMDRMSEMNESN
>HhamOR8
MKFFTFNIKVLKMCFLWPTDISRKENGKSRYAKDLIIIFSVMPCFIPILADFFAQLFEGV
PNLTEAVENMIALNCIIGMIYMIFCFVNNRHTIIDLMFEIDNFEKYGNSSITRDVDDKAN
LFSKVFLFYGILGNFVYMLMPQLSANKCHMARTPEMIQKGIPCGLVVRSIFPFKFDYTPV
FEIVFIHQIYTCTIVSIVVLILTTTLWGFLMHIVNQLEHLRSFVNSLKEDPQTKFLQRLI
YIIHYHNDIIRFSEKTAKAFSTMLLFYVTLTSLVLSVLCFEIIMVNDFEDSLRFSLHFIG
WVAILFSVCYNGQLVIDESIGIANDIYSLDWFLHPADTQKKIQTILMRSQKPLVFSAASM
GVISLPEFLKVFSSAYSFFTLLLKLK
>HhamOR9
MKRDEKQLKWRDVIRVTEKMLTITEIWPTQQNSLIRTIKITFFFICCIIFDVTLFDELQN
LLELQDYQALAMHLSTFSLYIGFSIKIILFQFTKIEPMGSMLRSIEDPIFDEYPPALEKY
KTKCVRISNGVALFYFSSVAISILVYLLKPLYTEYTLPITFSHELSFVTHYALYFLQSFC
CYYLVMIGISYDLIVIGLINIATTQLDILREQITNFKPEHQSEKLMEDEEYKFFSRCIQK
HEAILKYVGETEDVFTLIFLAQCLITIFAFCNGLFQLAHNGELFSIEFYFNCSFTFDVLF
EMGVPCWFATLLTNKSVEVSDACYNYNWLHSSTKVKKLLLIIQCRGQRPLYITAGKITHL
SLQAYLQVLKSGYSYFALMQTLYQQKNQW
>HhamOR10
MYEPKKNYQFYTTLRHMQFLHYYPRPCDLDDDGNFTKKYAILSLIVTTLPLATIFIILVH
CITAHRNNLPIDTTEDIAILVQFCNSMFILGRFQWHFQRTNRLYQAIVWTNENFGTTIQD
TVDKCNIYSMAFLIYCYLAATVFLITRLVFTKANCEKINQDKNINEICGTMFPAWWFTEV
DSIFILCLINFFNLLPIGFNVIPSGLIMIHIWEIAIMVKEKIRQLKENISEIFDDSNPKE
NLRYCIKYHQAILQVCTEFNLFVKGYLGHMSFMSAVVLSYLLANTIKNPDYGYIFDFAGY

IFTQFVLCYAGQVIYDETIDIKNAIWKSNWHRDLTLAKDLQIVMMRSQKAMFLEGFLGGS
FNFETWILILKTAYTYTTLLNSQ
>HhamOR11
MSHDIFLKIPKFCLVLGGFWPFKISDNPLYVAIYRVYNRFQTISYVCFIASLLINLILLT
LRHDQPTRIFASINVFIIVMETCIKLLLFQIEKIPDMFAHTMRNEEMIFSSDDYEIKAEY
FRQAKYCRKVNILQCGSTTFSCVTFAAVALIQLFKADDMSIYKKQPFMHDIWYPFLSIEN
HMGVVVFTNLFVVCQGACFNSATQCTFIGLMIYSSMRFRLLHIKIKKFGLTPQENPLALL
EELIVEHQDLLQFVKTLNERTKYVMLLEFLLNAVSLASGLLQLVMIKTITQLFSICAIIL
LQLIQIFVLAWSANEISVASLSIADAVATSNWIGQALMVKKLLLIVLMRAQVPVGLTAGP
FFNMSTVTAVNVKILLPVYIHIFQLYEGYFFRL
>HhamOR12
MDNRSIIQHLCNHMIFAGIWPNPQIRNFFLKKIYKPYITIMYIAIVITGICLCLEFEMLL
RDLNSKKMINSMTILFPSILIMIKIVIFKSGKVMTLINTIVKEETKIFTLENEEIQKIYN
GFVDYARKSASIIVKLIIMSTGSTLFGLSTALMSNQIEEKPTIVLVYLPFDEDKYYGASI
TIQIYWYCTASIYYSLFQNMYISLMTFVKGYLRILQYKFENFQAKSFIEDTQRVKSIIYS
HQFIIKFVDELNEIIKWWLLMEFLFSSVNIACVIFGLINANIFKDTINAVSLIIVLFIQQ
FIFAWHANEITLESYQISLSIYKSRWYNTTVIKNLLNTVMFRAQKPLYIMLGNFKPLTTD
TVISVSLRSLYLIYLSTFLLKIH
>HhamOR13
MSLIGLSAIKATGIKGILYRWYFRIIFSYFVLFIFTQIIQVCLYLKDGNKEAFAAAIGAA
LTYTTCLIKVNVFKSASFNKIINRVTKQEQELLADTTYDKNVRVIHKIHVISSKKLELGY
VIFILFGGLFYIIQPALKRLLLNYDTTSSLILPSWFPFDVKDYWWTAYCLQFLPCLCGSS
YGFLPQCFIQTLLGYIVAQYEILKYLFKNFSDACNYSLNVKGVEMNEKNQIMFLKKLIRH
HQFILSLMADINKCLQTAIFIDFSVSSFQLAMIAYQLMIVQGLDRAIVSIYFFATNVQIF
LLYWKGNEILYQSQQVAVSIAQSEWNTYSTKISKMMQFVMLRSQKPVYLSIGSFAAVNFG
MLLQIYKTIYSFFCLLIK
>HhamOR14
LKIYGKSAYFYLTTRLCILYGIIPWQLIFNHNKFRQKIYRIYSILIIGCYVLMYATACIQ
LGTLFSEDDLSIKKLSENLTVSFINGITIARQLVIKSNAKFRKILRKAIESEQQIYDCED
EKTSRIYTRFARNVNRNVIRYLASLSILFIQFCVIPAITDPVEIQVNNNRTKLVRALPLP
LWFPFDQQKHYTMTYFLDLLNCCIAGGFLTCTDLLMVIMIAYPLGQLKIVQTNLENFEIF
KRKFRYDGKMNAHLAFIEIIKKHYDVIEYINDFNDIMALVALIDFLQSSIQVACVFTQIL
EQSNFFTVGLVFSFFMSMIVRVFLYYYYANKVTSLVNLAYSIWTCNWYEQPTEVIFMMRM
FIMRCQKPLNFKIGSFGVMSMQAFLSCTYGILT
>HhamOR15
MKSLIKLPKLFMIFVGVWPSKFVLNQHTRRIYNVYRISFVAIFILFYMSLATECLLLITN
SSQTERANSASSILISVSIGAIINFTYYINELALMYELIATVEMEVFNMKYDNDIRRSYE
NTLKYAKYLYTSIIIPSAFSVITYTVTAFAELKTVGYSGWDFNKKSFMYELYIPVDKFKY
FNWIILFNLYVGFVCVALHVVYHIMFYGLVVFAAEQLKSIQIPNVDSHSIIKRFVVDHQD
LLKFVNSLNQKVKFPILLEFCVQSFTAASVLFQIITTRSAADLVFPSIFILIVSLEILAL
GWTSNEIKIQSLSIGDAIYESLWYKQDEYTKKLLLIMVTQAQKPLSLTIGPFQPMTTDTI
IFTAKASYSYLTLMKNLLKKY
>HhamOR16
MKLHPSIYKVTKLFMIGVGIWISPFGDKSMWKNVCLKYSLIVRGVYFVSNASLIVKAVIL
VFHHTEHQNVFGIVSLMALITMINIKVVVYKKSHIPELFKTIAQQEDSFFQTKADAEYIE
YCNYFNKIWIIQIITTTTSVYLYIIFHFYKKFTNQLLEEENFMYELWIPFIKNTNNFVFI
VIKLILSQMGILFHCTIEMVMLSLMIFIDAQLKMLQAKVKKLSKEDRVRNIIHEHQFIIQ
FVERLNKSINYVLDSINLAAGMLQIVMVSSECDGNYFFDSLFLLLLLQLVILAWNANEIT
IQSVNVAQAIYESNWYDQSEHLKKILLIMITRVQKPLYLSIGPFRPMDMQAGLIFEYYMI
KIFYFRL
>HhamOR17
MNNVYACFQCFHLPLTCHILREADSEPVDSYKFGVVCLVSLWGVIWINGFCIKNRHLLVS
LVEALSDFEHWEPPPAYNDFCRKMDWFSKIHFLYLSAGCSAYFLGFAPMHSKRCEEINRE
LNLTEICSLLLPVYVPLIEKSKFGEFPTFLLIDLIQCVVIFYMYVVCGTIVWLNVEMVEY
ICMRIRHLKIMLMDALKHPNALFRQEHFTKAVKYHGVILRMERLADKFFGTELFLHVVLT
GAILGVTVFVMIDGKNVDPVMILIGWLNAILMGCLAGQRLINESSSIHDVLFETDWYDFQ
LALKKDLIFFLLKAQKPMRIRAGNVIMTNALIIQILRTTYSYTAFLSSINK

>HhamOR18
MSVEYKKSFLGLVRPLILVAGFWPLPLTYQKCYNIYSIIVKFSFLICLITILYEMLHILQ
HHYDLNIIITTFGMVITVGKMLIKLSIYHKFNTWALLKQVVEKDEEIKKSDDENVKASYL
KKIQLINIYVTILVMITAVGQIGICTSGEYNSFQLTNSTKKQYLMFKSLFPHQMDNYYYV
LTFQLYWAWIIGLINTITTVLFLALLVYARFLLEGLQIYSGKFIIDLLANQKGKNNIETF
KKFLDRHCYVIEFVQDLNQHLKYITLLQFIFDSVDLASILSTTEFKSGLENLWLYFYVII
LIIQIYLLGWNCNEITVQSIQLAETLYDTNWYLLSREDLFNLQFIIARAQKPLFMTIGPF
SPMTTSSVITVWKYI
>HhamOR19
MVLLNLLKYSQYTFEVTALWPQNKSSWKFLSRTIFTLMVIGIFAVCQLYNSALLLPNFIT
FSQSLYFLISKQFLKLIDRIENQYFTKYERVYQHYLVNVDKTLYQVNWVYCTLVMISSIF
TGIFPGNNSNWIQMKISVLVVDPSKPLPMDFPHFNDGPFHIPFYIYEMVSLLVAAFCHIA
MDMLFVGLTTVALVQLIILNNKLMDKNKNIKYSKFYCDGNRHKLTVEYIKECCEHYIEIE
GFLENIENIFSVILFIQLGISVFAICNGGLLLISVKIFSLQGLSVIFYTMALFIELGIYC
WFGNNVYFESLKVIHSCSLSHWYEESNGVKKMLLILMERTKQPLQIKALNFTTVNASTFI
SIILKWSYSYFALLNHFTNKFPFK
>HhamOR20
MKCAPLFGVFPGDILFPANKFTEKCYSIYSRACLVFLGIFLLTSYIQFVIIVFSDQIDYE
ELSRNFVIIPIFTVTMVRNILIQTPRFAEMIRKILRTERILENAHDKEIREICRSHSIEL
NRNIKIYLAMMIITEFSFISRPVLTPEIEIQINHNETLLVRELSLSLWLPFNQRDHFKGA
YMFQIVYVIFVSCFETFGEFVLIAVIVFPIIQLKVLQHIFYNLEHYYMMMMKNHGFRTID
EAAFHTLKQCVDWHKEILNYVDEFNSLMSTCMFLDFIQSSLQIACVLIDMLTTKMVLMQF
VAESGYLGILMFRLFLYYSYANDVIVMSQGLALAIYQSQWYEQSGQVKFMTFMLIMRNQK
ALNYKLGIFGYMSLNTYLSVSYV
>HhamOR21
MGNSSNQQLISYLKILLMCGGLWYYPISKNYFVQKLFDFYSLLARIICIAFWFGLTGEFL
RLIIFKYDLSLILSSASVVFNDTKICLKVIIFLKYKTLNLLDEIMEKEKEIWMQQNEEIT
KFYKSMVRTAKFFLAGMVGSTFMAISLLEVSGVLVYFAIKESNFLNNRTDEAHVMYQTII
PLDKMKETKYYFSTQVMWSYIGLIFNSSTHGILTVLLIYVKTQLVILQIKLRNLIGADVS
EFEEVDQESTRITFIKLIGDHQHIIGVVAYLNNHLKYAIMLDFLLSSLDLASVTMGILQT
LAARNVLTLLFLLTFLSVLVFQIFILSWNCNEVAIESSAVGDALYQSKWYLLNRETQAMV
LLTLLRTKKPLYMTIGAFGPMTTASAVLVLKGAYSYVSLIRG
>HhamOR22
MHFTTFKQNIFFDWEPLPLMKFLAMLSRRIGRYKVPYFRYLGFIFGIVYLFCQITCYTFF
PHNDIKYIFNLTISFYLVVAVICQTFIMNIEKLMVVQQEFYLPLDFGGPRIKKTIIKAIQ
YEEIRCLITTLLIIVVIPCKINPNTLSERDHVAAILQKFCPKYEIIYLLIHTLFYLLSMP
SVLAWHLVFMYFNWHVQFQVRTLIAHVERSLNYDECIADQLYQAYVRKQLKIVLKRHSEV
IRYHRLTMQHLKVSITSLLLGLFGSFVCFMGFWIIRVYHTMDSISIVLAFLFAVDSLGYS
AQEYQDQFLTVYDYALKVPWYQWDISNRKAYLLLIHKSSLMIPYPFFNTCVNRQFELELL
KTIYSLGSIFINI
>HhamOR23
MPDTSNEQLISWIKVPLILAGLWNLKISDNVFIKKCYFLYSLIMRTGCFFFWVSLVAESV
RLIVKKYEMSIIIPSVSLTITEYKMVAKLLIYLKHDTLNLFGKIIEMEREVWICTSEEIK
SLYRKNILYVKIATSFIGFYALLSVSILQISGIIGTLHIKTHNALTNDTVEPHLMYPLIL
PFNKTQHLHWHFSMQLLCAWNGVLYYLLTELIFIVILVYAAGQLQILQVRFRNFLEPNFS
IDATDEEIRAKIFVLKGLVREHQNTIDFIEDFNDRMKYGTMIEFLVTSVDIASVCLGLLK
VICFVCLLIVYKKNCTNHANFMKNNGIYLGL
>HhamOR24
MAGDEINILETARCFVVAIIINVPILIIFLVQQQPYLQMLFKDIYDTEEFFRNTDDYVLK
EIYDNSVKNYINNSKILSILLSGVALVLFSRPFVEYEEHLENGVVDIKRPLPADLWFPYD
TQTHFWVNEYYVLRYMGCIQCAIFSYFVMFCVIFYAIGQLRIMHYIINNFKTYQSKLVPE
ESEIDPAEFTFKFLINKHQKIIKFIDTINKALRLVVMLDFLQNSIQITCVLVEIYEVLKR
KGELLTNITYQVEWYNESQLFKFMLKMFIMRCSKPLFIRIGSFGVIGLPSLISVRINLDF
LF
>HhamOR25
MGLLNMSTLMLQLGGAWPLEEGESKPKKIFYKMYGNFLIISFVVFNVFLSLGFIRLILKK
ESFGRLSNSLSVLITLLLMILNITIFNQKKVAYLCQDIKIYEKNYLLNAKDPEISVIYEA

ILKKSKILNTFTLVTSFFGTLSLIGVSLLWVHNAGSNFWESDAPFMFELYVPFDRQKYYG
LVIIANIFIACLGTIFYIAVQTTFYGLFMYGNLRFQILQLKFKKFSTYNEEDSFEGLKML
KTEHYDAINFIDTLNDKINHAVMSTFMVNSIKLASGVIALMDQHIEELLFPMIFLTVIFA
EVYFLGWTCNAIQDQSLRVAERIYAIQWYDKGKNFKTMVQMMMMRAQKPANIKIGPLGIM
TMRTILSTVKTAYSFITLILNLR
>HhamOR26
MELEKSFPLHHLLAVKFKQSIEPYKKVTIFLSIIGIFLIILQFVTLFLKKNVDKANFLLV
SAISYSTTSWITVIYLMDISSLLKYYKSSWWSLSGCFKEVMCQDRKLLLSNLLWILGLLY
PLAGNENIRNIDCAYAVLKEIHPAFAEIYFIIMTTYLYVTSIVVVALPATLFYSIWMQQS
QFSKLAEYIDETLGVTVLVIDENYHNNVSEKLNFIIKRHVQLLGFFHKRTEYLSIKITGF
LFGLCGGFLAISAAVNIIKGERIFLSIILISIVCMGLEMDTGATQAYENAHEAIFYAAMM
ARWESWNLKNRKTYFMFLHQTSQMNVFTKFVNVRVNRSFQLKNLFSGYISNRSALNGNGQ
TSEYHKYKKNLEDTF
>HhamOR27
MPEHTPQIFRYLIIIFKIFGFWRQKISNVLFYKILYILFLIVSQIGFFSFGAGLAAKLID
IYFHGYSVALFDVLMFFCSFFTLMSRNILVLTHGTQHIILRVVKEEKKTFEENIDFVKKI
FERSTIFLRRILNFLLVSTLFTILGLFLANSIQYVNHRNDLDNKPLPSPTIWYPFDENEH
YFSTLLISIIQPSCCYGVHVVGNSTIVALMIYAKTELTVLNTCMRNLHLHRNTEDEIFQN
LRKLAIKHQRIIKFVEELSMMIRFVILIDFCVTTIVIATSHTSLVTKMFQVFLIVSEWFQ
IFSVSWFANEIRIESTELSGALYETKWYNYSPRIKQLISIMMVRSQKPLTMHIGPFDVLT
INAGLRALRATYSYITLFLVMAPTEEN
>HhamOR28
KFFRFIDTINNALHLVIIFDFLQNSVQITCVLIEIYEVLKWEGCKTEDEIFKSGMLITLS
KLTLSLINQCLFNLSLTLENSGEWYNESRHFKFMLKMFIMRCLKPLSIKLGPFGIIGLPS
LISVRINLDVSFNSRTFFTFRF
>HhamOR29
MTSDILSPMLEEVEEENIEEDEENEKDDEPEDETSNIVSNPGDLLSMAPLSSISETSLAD
KESWKILPKNRLLQIPMHMATALGIWPILLTNNNLFKTIYKIVGSFLYYYHLTYVFRCYY
KLVLLFVAKPLDYVEILQNLCITLIYSVCRFRLRSFNTKEIKALFQQVIDTETKFLECPD
KEIEKIYLLGPLLVDLPTVVINNETVVLKQLPLSTWWPFDTQQHFWLAYSWSIFDGTLGT
ALVISSDMLTFSLVVFAVSQLDILSYRLSQFKNDKDFIELIRQYQEIIDYIEAFNKAMKY
VMLFEFLQCSFQWAIIILQLLVMQINVTNVIFVGEFFVTMVIRIAIYYFNGNEIIFKSRK
LSWDLWNTNWYEKPPQVKQKLIIFMIRAQRPLRFMIGPFGMMSLETFVSVFEFRWF
>HhamOR30
MAEIKVLSFHIQILELLFIWPKSSKSIINICFIYLVCCLMFVGITPTYWTIVSLGNYEIE
AFLEASTSIFNTLGYAVAYYSLLKNKGVIEKIIKDINLFQKYCPSNIIQEVDTKFTRLTK
FLFIYIILGISTNAVWKLISIDSCRAERGAKLLKHDPCGLPSRNYFPFDVSKPEIFWVYF
VLQNLCALHACLVFLMASSLFLGLLAHISTQLTYCAHEFEKFNYQQEWTTLNKDFLIYVN
YHRAILTYAKDVFRIFRSTIDVYIAITSVSIAIIGYQIVTSKDLDKRIIFTMLLCGWQTI
FFLICNSIQNVRDKSISVSEALYNSKWTTKLDKTSLRLNIIMIISRAQKPISYAIPCIGV
MSLERFLAV
>HhamOR31
MNKEQALSFFNVNRWMLRCAGLWRPEIKNQYWQRLYTIYGIVVFLFVNLWFTATEFISIF
YTMKNQFEFIKNINFFLTHFMGAIKVVFWYFYGDRLIGIMISLGTPGSLYEAYQSFQPQE
ILLKYKTIGQKYSLLFLSLAYATLISSYIFPTITALRCLIASKSGNSTLDLPSRLPYYSW
MPFPYTTPETYLLALAYQAGPMFSYAYSIVGMDTLYMNIMNCAAGNVSIIQGAFQTLSDR
CINRQLNKKVTENGHFENEVIVKSLKMEMETIIKHLQVTFKSCDDLEKLHRFVTLCQVTA
TLFILCTSLYLVSIAPPLSKQFLAECVYMLAMLFQLYLYCWFGNEVTLKFQELPQYIWEN
NWIATNTSFKKSMIFTMMRAKTPVYFTAGNFSRLTLATFMSILKTSYSIFALIKNTSD
>HhamOR32
MVLFDFLKYSQFALETTAVWPQNKSSWMFLFRTILTLGVIGIFAGCQVYNSALLISSFVK
FSQSLYLLNSYLNVCIKIIILQLKRKAFLKLIHRIENQYFIKYEQVYEHLIANVDKTINT
IQWIYWSSAIFCCIFTSIFPVVDPSKSLPMEYPHFNNGPFHIPFYICEMISLFMCAICNT
AMDMLFVGLSTVALVQLIIILNNKLKDTSKIVKCSKFYSDENRHKLTVEYIKECCEHYIEI
EGFLENIENIFSVILFIQLGISVFAICNGGLMLINVEILSLQGLSVIFYIMAMVMELGVY
CWFGNNVYFESLEIIHSCSLSNWYEESHDVKKMLLILMERTKRPLEIKALNFTTINANTF
VAILKWSYSYFALLNNFTN

>HhamOR33
MDDQQSTIKDNRSILKYLQTQMIVGGFLPSFKIENWILQKMFNFVVHLLRFYYMAIVALM
CFELFHLLPRGDMENVTRNMSLTIPGILMVVKLFVLKSRNAVKLLNTIIEEEYKIFSCES
ISIHNIYFSSVTHAQNCTKIFNIMSLLCGFAIFIATSLSGLLIEQLQKPLLILDQLSFNE
FNKQNHYGLYLTNQNIWIVLATLYYNLFLNFYVTLLAFVKQYKFENFQLKSRLEEFQHAR
KLLHSHQSVIILVNDVNKALKWWLFVEFALSSISIASVIIQVTLVSKS
>HhamOR34
MGLLDISTLLLQLSGLWPVENNKSKLKKTFYNIYRRFLVIWFVIFCIFQSLGFIRLILKK
ESFGRLSNSLTILLTVILMILNITIFNKKKVAYLCQNIIIYEKVHLLNTKDLKMSVIYQA
ISKKNKFLKIFTLLTSILGTLLFIGVSLLLLYNAGPDFWKSDAPFMFELYVPFDRQNYYW
FIIIENIFIVCSGSMFYITCQTTFYGLFLYGTLRFQILQLKIEKLSIYGGENSFEKLRSL
ILDFIDTLNEKISYAVMSAFMVNSIKLGSGVIALMQDLKELPFHMVFLTVIFTEIYFLGW
TCNGIQDQSFRVAERIYAIQWYDKGKDFKVMVEMMMMRAQKPSNIKIGPLGVMTVNTIVS
VRVLF
>HhamOR35
MWRHKISNFRFYRILYFLFTIAFQLCYISLGIGMVLKLILIYNQGNTVELFDLLLYIFAY
VTLIARNIQIQTPGIQRLLLKMINEEKRSFEENTKYIRDIFENSRKFLNRILKFLTVSAL
LTLAAHFLANVIQFINHKNDLDNKPMPSPGIWYPFNGNKYYFLTILISSFQSFSGSCIHF
VCNGTLFALLIYITTEQKALNSFIKNLQLCSETEQGILENFINELGVRLRLLFLIDFCMV
SVIIALNVFEMFQLPVSSSTKMFNLLFIGIELFQLFSISWFANEIRIQVFLLSTTLTINY
LEISVMHDRYLNSQSNKNYISLFLFH
>HhamOR36
MLLKEYPNENLSIIQSIYLEKTILIWIGLYPRFQSKYSILNYYLRKFLFYIFIILFILSL
YAFSIINFNERDKIFLQFFVAILASAILTKTLNLSINSHKIVNLEFSLTNKILNYDLTEE
QKNYITTNLRKVEKIANFFRIATACNLPNYIIFMAFFSSEKYGLPIPSYYPWGTENITTF
VINYSLQTFALLNLALINTTANHLFIKICAILYTEFHVLKWNLTDMNFNEIVRNKLVKNV
LLHQHIILMSKQMESIFAFGIFGQFFFSTIGICFVAMYLILPMNTENDTTFVQYLTGAYA
LIYMFLDITLYCIFGHLVISESLSVSSAIYSSNWYDIHPMLRKDLIVFRESARIPATLTV
GKLFPLTFETLIKARINNLIINEAFFLKIFTNFYSNT
>HhamOR37
MLDTSIYKASKIFMILAGIWKAPISRNPFIQEFYNKYSISIMTIFILNIFSMCFKIYQMI
PNRKLEDIYTDITLVIIVIEITYKVLIFLKSEISELFSIVVNREEEILISNDQEVKDIYL
KQVKYYKMASFWQCFCIGSGIVWFFGLNIYKKYVIGLKPNEHFMYQIWFPFDELKHDTLV
TSYNFFIGLYGLFFSCASRAPLQTLMCFSAAQLMILRQNILRTLEIEDEYVKFEAIKNLA
QEHQFLIEFVEKLNRVIKDILLLDYLMESVNGAGGLLQLFSITNLIEIPYCLIFIGNMMT
TLTLTAWSANKIIDQSRNIGNTIYNTKWTEQSESTKKMLLIMIHRSQKPLTLDIGPFLTM
DASATVSAFKAMYSYATFMRQVRNN
>HhamOR38
MIRYNFLRYPQHVLQIIALWPQNKSSWIFLFRMILSFTAIGSITLCQLYNSALLLSNFIT
FSQSLYMFISCLNTCIKIAVLYLNKTAFLKMIDKIDNKHFTKFEQIYQHLIDEVETTAGK
VHWNYCSIVVLTLIATGIYPVVDPSRYLPIDFPHFNEGPFHIPFYIYEMISLILAAGCNV
GIDLLFVGIAAVPLVQLIIILNNKLIDTKKNIKCLKFYSDEDRNKLTVKYLKECCDHYIEI
EDFIKNSQNIFAAILFIQLGISIFAICNGGLMLINLDLVSLQSLSIICYIMAMLLQLGIY
CWFGNNVYFESLKVIHSCSLSHWYEESNGVKKMLLILMERTKRPLQFKALNFTTINANTF
VAILKWSYSYFALLNNYKNTR
>HhamOR39
MSSSTQFRIMRLHTHLLKFLLIWPSDSFSEKLNWFVIHGCLTISIFFSLPVFSAVAYQFY
VGIDDLNTLLEALIGVLNIIISDVTYLSFLRKQKEIQKVIDDIYWFVKYCGFELIKKTDE
EIVRLTKCILYYVILGVTLNLMWPLLSIKSCVASRKSEFYIRHDPCGMPTQNMYPLDASK
GKTFLLFFTIEAIYCYHTCLFFSLATVIFIGFLKHISAQLKYCAYKFEHVFDDVSNNVNS
IEKVQQEFIHLIKYHQSIFQYTTNMFGIFDIMIVVYIGITSFTLAIIGYQIVSPTTNDED
RIRYTILLIGLVLLFYSISYYGQQVHDEFINIGDAIFKSQWYENGIALRLHLFLLIVSTR
TKRGFDFKVKLLGSLSLIVFMRVMKRAYQMFALLLTVANDTH
>HhamOR40
MLMEEVMGLLHISTLILQLSGLWPIEDNKSKLKRTFYKIYSNFLLVWLMVYDVFLSLGFI
RLIVKKESFERLNRSLFVFLTTIIIIPNMIIFKQKKVGNLCQDIMIYEKAHVLNTKNPEL
SAVFQAILKQSNFFTIFTLSTCFVGTSTFIGMSLLVLYNTGPSFWKSDAPFMYELYIPFD

KQKYSWFIIIVQILTAYSASLVYVTIQTTFYVLFMYGTLRFQILQLKIDKLSIYGGENSF
EKLRSLILEHQDIINFIDTLNEKISYAVMSAFMVNSIKLGSGVFSLIDQSSEGLLFPIIY
VTLIFGEVYFLGWTCNEIQDQ
>HhamOR41
MLINIFVCVITFGHAMAVGNFLISTTDITKITEIFLFSMTQVGLVNKLLNFQFRFQTIVL
LDKMVSTNIFKPSIETEQKMFENAFRKCQIVLNPFLVLCFLTVASFGGVPALANIKMKTK
IYPFPGKFPFNPDNYFLLIFGLEILVIAVSAWNNGSMDCLFVKHTVIATTMFAILCEKLK
NLLNVNNGKPGSVEFRIKKAVIYYNTIIRYVLKIEKAFSYGIFVQFLCSAVIICLTGFQL
LVVSAGNAELVRLVIYLLVMTYQLVLYCWYGHILMEKSKEITNACYTGNWHNMSVKEQKM
LIMVMERAKRPVSIRAAGIFQLNLSTLMTILRSSYSYFAVLQRLYVPTY
>HhamOR42
MNFTIFKQSISSDWEPLHLLHFLAMLSGRAGNYKLSYIRYAGLIFGIIYLFCQITCYTFF
PHSDIKCIFKLTTSLYLFSAVISQTFIKNIEELLAVQHEFYIPLDSGGARIKQKIIKAIQ
HQEIRCLIIIALMISVIPYEINPNTLSERDHVAAILQKFCPKYKFIYLLFHTIFFLSSVP
SVLGWHSLFLYFNWHVQFQVRTFIAHVENLCKYVEYPIYDEPIRDQFYQAYVRRQLKIVL
KRHSEVLSYNRLTMKYLRVNISSLLLGIFGSSILDHAVNAMSLVIAFLCAIDTIGYASQE
HEDQFLSVYDYALEAPWYQWDIQNHKIYLLLIRKSSLTIPFPFFNTCVNREFELEDIRCV
YQPLKYFCSVYTTGTSNFPTSKLSQVGIYTHKNKLKMRPKKNMSTIVGSVSLFEFMRITL
SDDF
>HhamOR43
MDDPQSTIKDSRSILTDLWQQLIMGGFLPRFKIENWFLQKIYKFYIHGLQFCYVTIVALM
CFEFICILPSGDMEKVTLNISFSIPSVLLIMKLFVLKSGNIMKLLNIIIEEEYQIFSCEN
TSIRNIYYNFAAYVPNCIKIYKIISFWSSLAVLIVTSLLGLLIEQLQKPLLTLDQLSFNE
FNKQNHCGLYLTIQNSWAYAKKLLHNHQFVTKLVNDVNKAPKWWLFVELAFSSFSIASLI
IQITLARHTSSVGFAVVVVITMFIQQFILTWFANDVAVEQKAYMYLSEWYRTSTDIQILF
KIAMIRSRKPLYIYLGNFKPLTTQTSW
>HhamOR44
MGFENLIKIPKFQLEMLALWPKKMSPMYWFRSVLTYFGVFILTASQLYNSALLYSNFVKF
SESLYILISLLNGFEKIVVLALKKRTLLGIIEKLNTTQFTKHEKFHRDLIAEVEKIINLM
QWVFSIMATLCLVPTVVSPIFDSKSFPLEFPHFHDNPYYIPFYLFQVWSLILCTYSNTPV
DELYVGITAALLTQLRMLNNRLKNTKKNIQEMKFYSQESRDKFIGNVNQSQTVANIFYVL
TLFVESGLYCGFGHYIYEESLKISSSCYFSHWYEESNDAKRIVQILMERTRRPLEVRSLN
FFSLNFNTYQIIVKWSYSYFTFLLHNFQNQH
>HhamOR45
MDASKSAIKDHLSILEHLRKQMIAGGFLPGFKIENWFLQIIYKCYVHGLRFCYVTIVTVI
TFEFFYLLPGGNMKDVSLNICFAIPGILMVIKIFILKNENTMKILNTIIEEHKIFSCESI
SIHNIYFSSVTHAQNCTKIFNILFLSTGYTAAIATSLSGLFIEQLQKPLLILDQLSFNEF
NKQNHYGLYLTIQNLWCFLSLLYYNLFLNYYVVLLTFVNALIRILQYKFENFKSKSRLEN
FQNAKKLFRNHHYQIIFYRLVNDVNNALKWCLLMEFALSSSSIASVIIQVLLVSKNHISF
VGFLVIMVLILFIQQFILAWFANDVAVEVIKFIAHIYSSEWYRTSADIQ
>HhamOR46
MQACVMDGLMLWVTGIAYIACQTTFYGLFMYGTLRFQILQIELDKLSSYKEEDSFERLRS
LVLQHHNIINLAIMSTFMVNSIKLASAVIALFVRILFTTIFVTVIFAEVYFLGWTCNGIQ
DQSLRVAESIYAIQWCDKGKYFKVMVEMMMMRAQKPSNIKIGPLGVMNIDTILSTVKTAY
SFITLILNLR
>HhamOR47
MDEPQTFVILRLHVKVLKYLLIWPLNFLNKKQNVYLRYGFFYYCLCTSFPVFSAAIYQFW
VRIDDLKILLEALVGVGNITGYIIAYVCFLKKQKQIEELLYDFKQFIEFSELKLIEETER
KVAKYTKYLLTYVTVGLMLNFIWQMFTTESCELQRGSEFYVKHDPCWMPVRNIYPFDASD
RKYFWVVFPIESIYAYHISCFFSLATSTVMGFLMHISAQFKCCLDKFDHVFDDVEDNEIS
HNKKLDEFIGIIIYHQRILVYSQKLFKVFNAMIIVYISLTSLLVAVIGYQIVNPKINTTD
RIKYIMLLIAWCLLVYLICYNGQRVQDDAVKIGQAIFKSHWYKQGVSIKLKKYIMFTIAR
SQRPLEFKAPLLGSISLMEFMRVIFGLETLILF
>HhamOR48
MGLLNISAVLLQLSVSWPIEDNKTKLENTFYKIHRYFVVICITFFCIFQSLGFIRLILKK
ESFGRLSDSLLILLIITLLLVNKIIFNQNRVLYLFQDIIIYEKAYLSITNDPEMLVIYQA
IVKKSKFFNIFILLSCFLGNLFFIGVSFLILNNEGSNFWESDTPFMYELYIPFDRQRYSW

LVIVVELCMAYSSSLLYVTIQTTFWVLFMYGILRFQILQLKIDKLSIYGGENSSLILEHQ
NIIKFVDSLNERISYAVMLTFMVNSMKLGSGVIIIVLMVQIQDLKELPFHMVFLTVIFTE
IYLLGWMCNGIQDQSFRVAERIYAIQWYDKGKDFKVMVEMMMMRAQKPSNIKIGPLGVMT
VNTIVSTVQTAYSFITLMLNLR
>HhamOR49
MGWLKEELISICQAIFSTWSIKNNFLTQTLKDKDYELSLISYVYCIKLPCHYKVLHKFYW
LVVYLGILSILLEFICILTTEMSLKIFIQHEFFPLIRFIGVILLIIFLQQRDEIIRAEHQ
TRKLQWNLSGLTTSIKRSIAKRGIEQSFILEILSFFGILYICFRFIELKKDIGNDRLYYS
WIFRNCPSIYFTVQSEIQILLLANYVRQLSDKFYETNKDNQVYICLKAVVKRQMELKRYF
LDSQELISYAGFQYLFLVFISVTFVSYATLEKQLNLGISINSAVIFIISSTYTMYNGQKY
QDAWEEFYEALCSMPWYTWSRKNQEFYYTIILGARKNTQVVMLNNVSINYDLLLKVWKYA
YSILNFVYRTHNK
>HhamOR50
RTFLRKTYKPFYFPDINYELSMVTYIYCLKFTCHYNILNKFYWFIVYLTAIFLLLECCCF
LRTPMTTAVIIEHGFPFVVQLIGGCCLVTFLSRRKLILKAEKKTKVLQWNLKHFKPSVKR
AIAKRGVKQSFILEILALLGMVFVYFSIFELKQSLFLQMIMRYDKHFLTWTVHVLQILDL
WIVAYFIIIMPFIQIYFSVESEIQIFLLTLYVNKVSEDLRRSKHHKVVHNHLKTIISRQE
TLKKLEKLFFLIELPLNNMMAFTILSIFTMYNGQRYENEWSRFYETLYTMPWYKWNLENQ
KTYLLMMTGSSKILQIKVFDTTAINYILLLK
>HhamOR51
ISYELSMVTYIYCLKLPCHYKILNKFYWFIVYVMAIFLLFECGCFFKTPMTFAIIIEHGF
SSMVHIVGGSCLIIFLNKRMEILKVEEKTKKLQWNLKHLKPSVKRAIAKRGAKQSFILEI
LVLLVTIPIYFTISQLKQSLFLQMVVKYDKIFNMDHLLESEIQIFLLTLYVNKISEDLRR
LKHHKIVNNHLRTIISRQNTLKKHFLDNQDFVSESAVRYLCLIIIVLPFVFMSSSQRKVP
LGTAIISIMQFAILSIFTMYNGQRYENEWTHFYETLCTMPWYKWSLENQKTYLLMMTGSS
KILQIKVFDTTAINYILLLKDS
>HhamOR52
MDASKRAINDDRSILKHLRKQMIVGGFLPCFKIENWFLQKIYKCYVHSLGLCYITIVALM
CFDFICILPRGDMEKFTLNTSFLIPSILIIVKLFILKSGNAIKLLNTIIEEEHKIFSCES
ISIRNIYYSFVTYAQNCTKMFNIISLLSGSAVVIIMSLSGLFIEHLQKPVLILDQLSFNE
FNKQNHYGTYLIIQNIWAQRCTYYNLFFNFYILLLIFVKAQLKMLQYKFENFQRKSRPED
FDYVKKLLYNHHCQTIFYRLVNNVNKALKWWLFVEFAFSSISIASLIIQITLANHITFLG
FTLVVVITLFIQQFINDVAVEVTKFMAHGSIYLSEWYKTSTDTQ
>HhamOR53
MNSESLTPRVDQFIKFHKKYNIFIIYPCLIFWIIILLGELCFYVGVFDRKHLLDYPPFCL
LILYSVTINLLIFLKNEKFSLFYKKFNEIISPDLSNVELEMKKIIHASCNKVELLLVIYI
GINTTIVATSAYDGIFSEDTFQIQSFVLKDSILFRPVQGILYSFILIWNITPSALLPLYY
LIPMLYCKIKLLQLVDYTNKINHAGEIFDANDMLEIIKTLIEDRIKIKEYHQIIFGEFQF
YANIYVLNGVSTPLKVHGLLVCAGYVFYCYTIAEFAQEYSDSAVLCTKAVFSLKWYQWDV
KCQKSYLTMLIQFSQELKVPIFSFLDVDLDIFKKCVRTSYVTANFLYTLKHKNKYKN
>HhamOR54
VQLKSLILNVNFIFIVYHAWEFALNVIGTSFMGFFLYRQDITRKLAEFKEMRCWNVDSTE
RKIQKEIKRDARKKNAVFIWILILAVSVNITAGMFYSNFMNENYLLLKLIGGFSESRLVY
FVILNFAILDTLSVFSAYYFFYYFFHLKYQIQLFRDYITKIGFLQGTQGKLYYKNWLSAR
HTGEVKHVLKFLLKRHLELWGLYMRKSGQAIILIFIISGGFLAVCMGMAALFIAKYFQPE
THFLFSNIMLLTFIYIHLLAYYGQEFENMFNELYLTVITTFEWTNWNYENRRIYLFIIQN
MQQPVKLEIEELIALNFVNEIR
>HhamOR55
MLPNHSHSKYLKHVYNFLKFGNVLLPIIHFSIVYHLAIQLFQTYYLIKMFTFKLFLQYSG
FYFTNIFVTSMQLIFKKILKLFVEQLKTSPGLYNVNPRFETDLLLKSYDSKATKQCIVLL
FAICVCVFSDIFYETKCGDSIVITSLILHQYFARTSIVSLLYHNVFYLTEIVAITATVGS
VIYFLYMGFHVKAHVIMLTDYICVFNQEEMISESHICVELKVISRRYSEIKKYFFSSMLL
GEELLFFLVLTGMASGLMTISVISTLTLVLFSFIITDLLDYEFLSDQISTAIYDLKWYNW
TIKCQKDYLILSMQIQKGIRIKIMGLYELNRLYLKQVIKF
>HhamOR56
MLEYTPQIFRFLIKMLKVAGFWRQKISNILFYRMLYIFYRIVYQISFNSLGVGLIGKLID
IYYKGYSVEIFDVIMFVCTYCTLMSRNILILTHGMQRTILRVIINLVAKLFLMGTAITLF

VLFALNINQYINYQDNLNNKPLPSPTIWYPFDENEYYLLTLWISIAQTSCCCGIHVVGNS
TIVALMIYAKTELTVLNTCMRNLHLYSKTEDEILQNLRKLVIKHQRIITFVEKLSMMIRF
AFLIDFCITAIVSATSHTTLVGKLFQIFFVVSEWFQIFSVSWFANEIRIESTALSGALYE
TKWYNYSQRIKQLISIMMIRSQEPLTMHIGPFDVLTNNAVLRALRATYSYITLLLVMAPT
EEN
>HhamOR57
NFFKSLICKDILLRIEEKEQRIFQSNNEAFIKIYKQNSASNRNVVLFYTLMGTIGISLYF
ITPIISNVTMPLGFNNQTGIKNHYFIVFSWFPFDPNRYYTTAYLIQFFGCLIGYSYIVHC
GAFYISILSFIRAQLKILQQTVSHLPEYCITYKRQFNLNEQHSQIVVLRALIQEHLDIID
FVTKLNDTIRSFSLMNFIISSFQLSLVAYQIFQLPVLQQITVFSYFITLSTQLFLTYNAA
HQIIIESENIASSIFEGDWYDYSPETSKFLQMICMRAQKPLAMTIGPISDMKITALFQIF
KALYSYICLIMKA
>HhamOR58
IIPYFWVFFWALNTVNVTILFFINLNDRHYGFFTEDFAYTIGEWCTNLGCIFFLIYEKRW
RALFLSFENFQYFGKTSTFKRIMKFGNVIAIIFLIYMTVGSLAYIVFVLVDENNCEQIYG
STRICGVINPTWWPFKNPPTLLVKRFLLVYQILSAIVTCNTFFVSLLIHQASNVIGDKAD
HLGHKFKQLCLISNEDDQFENFKLWVENHENTIRMTNIGVAVWLSNWTECNPKLKRWIPF
ILLRCQTKIEFDALPIGKANHALLLIIVKTSYSYLTLLRKTMNKNV
>HhamOR59
MEEEIRKIINSVSFKVELLALLTGVISFATVVFTAYDLMFYEDRLFLQVFLGKDSTLFHI
ARICCCFFLLPAIIPTIALPMYFIFLVFHAHTNLLQLVDRIKKFDEKMSEGTIDDENVIE
NLKIMAQHYLNIKCFHNYAFEATHLYASVIGVGGFLLGLSPLLAMFTYNAPKGPALMLLS
NYILFLYALCDVTQEYLNIQEHLLDVLYDLKWYLWDVKCQKCYLLMMGQFSNELKIPVMF
VYYIGLEIFEKFIRLTYTAANCLLTLGKVIA
>HhamOR60
MGLLAISTFFYQLIGIWPIENNKSKLKHIFYRIYGNFLVIWVMVFNVFLALGFIKLIIKK
ESFGRLSNSLSILLTQLLMIVNITIFNQKKVAYLCQDIKIYEKNHLLNPRDPEISVIYEA
ILKKCKILNVFTLVTTYGTILLFIVVPLLLLYKTGPNFWKSDAPFMFELYVPFDRQNYYW
FLIIANIVTAWLNAVIYITGQTTFYSLFMYGTLRFQILQLKIDKLSTCDEENLFATLKNL
IIEHHNTINFIDILNEKISYAVMSTFMVNSIKLGSGVIGFMEIDIKDLPFSMIYLTLMLA
DVYFLGWTCNGIQDQSLRVAERIYAIQWNNKGKAFKFMVQMMMMRAQRPTNIKIGPLGVM
NMDTILSTLKTAYSVTTFLLKMR
>HhamOR61
MSEPDVVEITRCIAVANIVNVPMLMLFLIRQQPHFQKLFKEVFDIEEFFNNADDFKLKEI
YEDSVNNYINNAKILTIFCCGVAFSLVYKPFSTYRIDRQLSNGLIKVMERPLPLNSWFPY
DVQKHFWETYVFQVLWCVIFGLSVFFSYFVVFCVLFSAIGQLKIIHYILKNFQTYQDKKF
TFSNQFQSDPAEFTFKFCIRKHQQLIRFIDTTNDALNLIIIMDFLQNSIQMTCVLVEIYK
NELLINTAYCQINWYHESKHFKFMLNMFIMRCLKPMFIKIGPFGVIGLPSLISVRIQIYR
VPMTS
>HhamOR62
MSIRNSLFQVSNYFIIVSGLWKMPLHSNPIIQWFYQMFSHFCNMVFGAFVFSLCAEAALV
TMGMKNSADVFGTISAAGIMFDINFKSMVCLKFGISKCFSKLIEEEKLIFNSACKEVKHY
YKHQCNMYFKIFLIQFLTTSLTASSMILLNLLQVPLSAENFIFDLWVPVSINMKIQSIFK
LIICEYTIFLNTALRSALQSLIMFMTSQLGILQINIRNMSQDDLNNICVLRTFIHRHQSL
IAFVEDVNKSINYVILMEYVLDSVHIAAVMLQI
>HhamOR63
LILTKESFGRLSNSLSVLLTLLLMILNITIFNQKKVAYLCQDIKIYEKTHLLIATDLEIS
LMYQIILKKNKYLNIFTLLTSFGAMLVFFGMPLLLVYNTGPTFWESDTPFMYELYLPFDR
RNYYWFLIIVDSLMVWVTGIAYIACQTTFYGLFMYGALRFQILQLKIDKLCTYGGEDSLE
RLRSVIIEHHDIINFIDTLNEKISHAVMSTFMVNSIKLGSAVVANMDQNIEDLPFPMIFL
TLLFAEVYFLGWTCNKIQDQSLRVAERIYAIQWYDKGKDFKVMRPANIKIGPLGVMNMDT
ILSVRVFL
>HhamOR64
SEHNISDITTCIAVIFNINIAVLIIYLNRYCPYFRYLFREIYKTENLFENVDDHMVIKSK
IEKLKLLRYLFQLKKIYEESLHSYILTVKILIVLFSITCFDHILKPFYTYEHHQSDGTIV
IKRNLMLNSWFPYDTKTHFWSFWALALGSGIFFSYFVVFCLFFYAIAQLRIINYIMNNFK
TYQSKIAFKELQIDSAESMFKFCIEKHQHIIKFIGNVNKALGLVILVDFLQNSIQMTCIL

VQILESELLANTAYQLNWYDESQRFKFMLNMFLLRCRKPLFIKMGPFGSIGLPAIISVKI
LIILGI
>HhamOR65
MDTKIQDRTIHPGFLIPRVHNYLIFSEKYNKSLTYFFNAILTSFLLIEIYFYFSASNRIH
FSGYLPFYLCYLYILNTSLVQLKTTEIFVTLSDKFKDALRLNMDGVCGSVIIILQPYDIL
FYEDRLMLQSFLSKDSILYQMARIAICCFFMIAVFITVTCPTHLIYGLFNAQMHILQLLD
KIKKFDNRITNEKVDDECILKNLKIFGQNHLDIKSFHYEAFEANHLYSTLYSITGLMVGM
SSLIGMFFYNAPKGSALINFFGYISFLYVITAIVQEYEDALKHLSDALYGLKWYLWDAKC
QKFHLLLMGQVAQELKIPILFVIHADFELFKKLIRLTYTVANCLITLWQKNH
>HhamOR66
MKHVKTTMLLLGIWPVRRNGDFLEILYEIYFLTTFIYYIIFNISGLALAIQTWSANYLTT
ASSMGIVIEYVSNAYKVILFKTKTFKDILLRIEEKEQRIFQSNNEAFIKIYKQNSASNRN
VVLFYTLMGTIGISLYFITPIISNVTMPLGFNNQTGIKNHYFIVFSWFPFDPNRYYTTAY
LIQFFGCLIGYSYIVHCGAFYISILSFIRAQLKILQQTVSHLPEYCITYKRQFNLNEQHS
QIVVLRALIQEHLDIIDFVTKLNDTIRSFSLMNFIISSFQLSLVAYQIFQLPVLQQITVF
SYFITLSTQLFLTYNAAHQIIIENLAYRADETYSSISNVFYQMAQFFFHAILVALEVQIW
IKE
>HhamGR1
MKQMEIKDLADLYGEELDMKSIVKMRGSPRARAIAKRFALDENDGNILEEHDQFYRDHKL
LLILFRWMGVMPVERTIGRITFSWTSKPMLYAYGFYVITTVIVVLVGYERLDILLNKSRK
FDEYIYAIIFIVFLTPHFFIPFVGWSVAHQVCDYKNSWGKFQLNYYKITGRDLEFPYLST
LIGTISLGCLFLAVSFLLSLSTLLEGFELHHTIGYLHIVTMINMNCALWYINCRAIGNAS
NGVAESLRKDIDNYCVAYIIKHYRVLWLELSEMLQKLGNAYARTYSVYCILMMTNIIISF
YGFTSEVVDHGVKFTFKEMGLLVDAIYCLTLLFIFCDCSHKASANIAERVQWTLMEINLK
QVDEGTIKEIEMFLQAIHLNPPKVSLQGYTVVNRELIASIMGTITIYLIVLLQFKISLVN
LRS
>HhamGR2
MQSKTQFLGPYSDETNVIRRKSENIRIVTPATHRADHEPDEELLEKLDKYDNFYDTTKSL
LVLFQIMGVMPIERKKGKTIYRWFSPTTCWAYFIFLTETAFVSVVFKERLVLILKPGKRF
DEYIYGVIFISILIPHFLLPIGAWRNGSEVAKFKNMWTKFQLKYYKVTGTPVIFRHLTLI
TYSLCVFSWMVGILIMLAQYYLQPDMLLWHTFGYYHILAMLNCLCSLWFINCTAKGRVAV
WIAEKLQIALEASDSETAKKLADYRELWVDLSHMMQQLGKAYSGMYALYCLLVLLTTIVA
TYGCLTEILDHGLSFKEAGLFLIAFYCVTLLCIICNEAHAASRKMGPEFRERLLNVNLLT
VDNRTVQEVHMFLTAIDKNPPIMNLNGYANINRKLISSTITSAATYLVMLMQFRLTLMRN
AAIAARKANATMMMHNST
>HhamGR3
MATVVHLKPLLPIFSFIGVLPPINFNNNCVKLTKSYIIKVSALVLLFTIFSIASVIARTI
FLEHRPTPYKIVDGITTVLLWLLVILSLIYSCIHHQLYGKLFRQLNEIHKECHGDFRIKC
PMLLTRKFIFILICQFLIMLWYYKPPNWMWTERYWIGIICYFHEEAVFHFYATCMAFLVV
SLLKISKQDLKELRFKLQELRGLQIYEERPFLNQIKSKQKTFTKLHTITRILNHLFIPTF
LCIPLTCRTWLLQFFLYIIHMINDYSTTVEYQLASFCFGLMFVMVTFLILLACDEIMQES
KKLMITCYDLQDCISIYSEPYQELQRFTEKISKIKIKFSALGFFEVNKGLVFPIVGSVAT
YFFVIEQFWNRN
>HhamGR4
MKDISNDIRYDYGLTVSKKFYDNLKHLFFMNKISGLFPISVTRIEDHYRLQWSWLKVLLG
NFCVIIYATITLTGLSLSYKIEEVYNVRVKDLAALMSTLAEIISLLSTWFIGQISTALSF
KQFWKYLYFLTEFDAIMGTIEPTRKTPLVFFLVFLFTFTCLAMDIYMWAQIVVPIVGYYS
FLWKIVPFYFTYIFTMILEVNVWTTLYNISSRIKRLNTVVESYVNISTDIEINNKNERFN
DCGYHGNLIKIANGPKKTMRQFKMLTSSIVKDFIRAFNNITQANMALTKFLGPSMIILLL
NVISCLVITPYVLYQQILNAKKNYFLFTQSYWMTCHVLRLLLLIEPCHTGQIQGTELKKS
IVKMATLDLSKDTYKQVKILLLMVTENNIEFNASGMTFINRTLLPTIGSAVFTYLAILFQ
YKNNI
>HhamGR5
MDFNETWIHIVFTVSIVALSLPAFAAQLGVNLEVRTIITSYIITALIWECRRKVQILNDD
LLKANNTIDLNKIQKFHYVLTDAAKGVSEVFGSIIMVIIVISITEICHVILGITYIYYNS
RNGWKNYLIYSDFIAHVILLIINGELLKREADKTINICYEKLHKHNRNDDMNRHLLQLTQ
QVFQRRPRISAAGFFSVDHSLILFICTTTATYIIFLCQFESSKSTNE

>HhamGR6
MKINSTTKSLKKLIILGQIFVYFPILKNLTWKSLKMYYALFTITGSIFLCTMQVIKILNT
KKIDLDQIDYVLKYLSSAYVAVIFVRLAKRWPKLMHDWNNVEIWMRSYGFPMKLNKRIMW
LIGIFTIGFLVESLLHQASRIYRAWQCHEDDAEKAIKFFFGNLSLSHVFQHINYNIPSSV
ILQFWIFQTEFGFMYTDLFVMVISACLHSRIKQITERINLADKKQTVHEKIWENLRRDYT
KLSVLVQRVNEEISNIILVSFVPNVFTILTQVFATLKPKHNRLEAVYFYFSMIFLISRTV
MVCICGSLVNEESRKPLHILNCVSHTVYNDAIEKFINQLLSHELSLNGKHLFTIKKSTIL
DIAGAIVTYELVLIEFNQYYLNSWSKSTRCD
>HhamGR7
MKVLRKIKSETSLTIERDHETMCFNRQHKFHEDVKFVLLLAQCFGIMPLENVGKHQQDVA
FRFLSIRFVYALLHFGCVFATGIASVTKLAVKGFVIDETSVTSFYIFNSFASIHFMYLAA
KWQKILKDYSYVELSMRNYSCKRNVKKINLWTAVFSSEHILFILTNLDHSLSCKNFKFYP
VEIYFGSAFPQWFTLVTYSHWAGALVEFTNFISTFTWNFTDLFIILISISLREKFNQISS
RIEHSKNPPSKFWKEIREDYYKVSNLTKFVDTQIAGLVLISFLNNIFFLCIQLYNSIKER
EAVIDSIYFFYSFGYIVFRVVAVSLYSATLNEAARKPLKFLYSLPTENYTIDVSRLITQI
NYLPNGITGHGFFLITKNFLLQAAATVVTFELMIFQFSPVKTTAHQSFSNNSDSICIK
>HhamGR8
MSALLFNQFPMGSLFKSYFSARKSTTFQECITIPLRVLRFFWIFPVTDAGKSIEICSWKT
FHCFSCSLLQLSCLLLFYLQLLSRISLTTFLIKIVILMFYFPYLAFSWRNIIVQWKDVEK
TMSYNEPKFTIRRNICFIVVILFTTLIGNSGLQCGKIKVAMFQKVFIWIFRDILIAIISM
CFAYRLEQIQSRIVFLNTLRANCLTFINNIFLWKSLRCDYVKLTRLCFLLNRKFSWLIII
SYARNLECLLLDGYATINSYGSSSFAWNLCEIFLIIILRVILVSFFGGALHYKNEAILVQL
VTVPTRIHNKEVQRFLCHVSSKEIGFTAKNYFNVTRSLILRIANAVITYELVIIQFVEHF
NNIE
>HhamGR9
MLQNIPQQVRLFGPNVQLLKCFGIVPNINLKTNRLHGTIYSVIYSVLLIIVVGCMEIFIW
VHKFYMDYSDDSYLPVIVRITCELTGFIMFNCVVTWSYTKRKLWEKLFQEITSVYNQQTV
KEPQEKKRSVLRNANLYFVVITFLILLLYCMDIFFWQIEARSMNFILSYVYFYYNFLLSN
VIGHVALEIGSQYKYMTNALVASEYTNRENGRKILNDTKRLYIEMNKVIGTFNNLFGPIL
LLMAVQCSLQILDFGVFLVEKVLPNLKFEYDAFLVYVIFIIMDLTWFSVTQFRCEMAKDE
SQRLLTVCYNLLDNYNDGSELLQETHNLIDQIKNRPVQFTAADFFEIKRSTTFSIIGSTA
TYFIVYVELRSNDSSWTQNNGTI
>HhamGR10
MYSNNPPGTPLPPGLMSVLDNSLQGFDQNAFLDGSRPYYKTTKNPITKVAPAPPPQYMNG
DNTAIMEAFDDDGELKSKIFDTIKPVFATLRILGMLPLSRQGPVFDVTVKWIIYSVLVLS
GVAGFLGYLKYFKINVTESGEGRFEEAVIDYLFTIYLVPIVMNVIALYEARKQANVLTQM
IAFERIYSRKFKRRLPIDLGSKPMVIMLVLIILGSGIMVTTHFTMANFVIYLVLPYCYIN
VITYVIGGAWYIYCDLIGNIATSVSEEFQYALRNIEQTSKVADYRSLWMMLSKMIRNVGN
SFGYQMTFLCLYLFFVITLTVYGLLSQIQDGMGVKDIGLTITGVSAVVMLYFICDEAHFA
SSCVKNQFQKKILLIELTYMSEDAQQEINMFLRATEMNPTEMCLCGFFDVNRNLFKSLLA
TMVTYLVVLLQFQISIPAGENEMFISNSTMNATHPTPMTKN
>HhamGR11
MVPNFSRKSIISIQWLCNFCAIFGLMPFYSFANKRFKLKRTCFMVFMSLICIVITGTAAD
TIYRIVCIDEFRLVHFMVMVSEFFGLLIWLVAAYQSCFKHNTWQEIFNGIQYFEDHNNDR
IKERNIWKNTNLQFIVTVILVITILIYERITFNDSNQNVFLFKYFFKTFSSHTKLIFVLI
PVQVIYISSVLTLTLIIVFLGTRKVQDLYLQATTVIEYFNKLFGPLLFLMGLHCSALMLQ
FSVFVVYNMFDKEQQHDDYSLLIIGVVDIFLMLLWLGVTMFCCDNVVAESERIITICYKI
QLKHSIFSVSYRSINNLLYVVTKKKVKFTAMDYYEIQRSTMFGVIGTTATYFVVLIQFYD
GNKKRPTV
>HhamGR12
MTCSPDLTLLLECFFILTIPRWCKSMKSKIIWTTCITLIGIFLMLTVLVMINPMSQKMEI
NQSLDIQNFMFLIAACVANLGINFILNSRSADLNTLRNKKTFSVIIRITIIVWFLNFFSR
NIHIIGIFASHFNSFLLFKILVGNYLVHRLQAAILYLGLKLYTLDKDLCNLNKKLKSWQC
LNIVSELNIQHHHLINIFEKIGSITGIVVLFEFVFYIIELLKDFSFIIVSQQEEADIPFL
NYRLLWAIVSAITFSAITCIATFFFLSHAVQCLTDTATKTISICHRKIENLDRALILRER
LDLLYHYEIIAQETYHRSMNLNAAGFFTIDHSMILLLLSNAFTNLLALNQLKFTGNKSTE
PY

>HhamGR13
MGQQRSTSIRHKMWHVLCPAFSFYVIFTTEGSFTNGRRGAMEVLKLVTDYISPLSYFRQL
IFPQKEWKQLLRIIQEYSKLKDCMKYRKLIYEYTLVLVILHGMFIEMLVSKSMFIEESVT
KKVISIINIFGLYYEFMSAITFSMIVILGVKLKLMEMHKILRESDKKKLTSMSSLKRLKR
LYLCALDNYHLINNHFGIKLLVCYGCSLTRLLCGLIITADHWKEKLEANDNSESSYSFKQ
VNKFFFFLMCTVAPVLIITKACKELSENVTTTLMKCYLLQIKIPYKSYEYKELRILWVFI
FENRLWFTAAGFFTIQPSVLLGIAGSTVTYFLVLLQFN
>HhamGR14
MDINGNCLGSLTNPFTIVPFTDSLTLIITQNTYEPVIFSVIFSILPDSRTSIRNCKGYLL
KAKNLILLMWFFFCYIFSFYIILTTEGSFRNGRRGAMEILKLVTDYISPLSYIRQLTFSQ
KKWKQLLRIVQEYNKLMDEYRLVIYRYFLFLVVFHGISAKISFTILSLPNVSFARKAYFL
QCILGLYYECVSAITFSIIIILGIKLKLMEMHKILRESDKKKLTSMSSLKRLKRLYLCAL
DNHRCINNYFGVKLLICYGCSLARLLWGLLATADHWKRSTQPTASPQASSYDLTEINKFL
VFLFCITAPVLIITRACKELSQIVTTIFMKCYLLQSKVPYKSYEYEELRALRIFIFENRL
SFTAAGFFIIDPSVLLGIAASVVTYLLVLLQFS
>HhamGR15
MFFEKKSIKVNLNELRIYKAVHLRLIIYSLQVTPKVRWLIYAITFLVHIVTTTISMYGKF
YYFYGTILSSENLAKFMDAFTELILAASNLLMQTVYLKNIHLFEKLVDMTDNLGIGPQSI
SSKIVTFLLHILSISTMIINMTVLSQVFEYGKTQYMFFKSFEYVSFNFGFGLYGQLNGVL
KTDILAIFKKKETDLITNVHKSNYEQLYFIYLIKLKFVVKTYGELCDLLDEYNNKFKVSL
SLMFFSTILTFLYSLIIYLKYEIINPNEDSRRIFLVMSLSLIWITLIGHVTLAAWMGEQL
VEEGNNTTRICYRLLGNLPEDLPRNLLEPLEKQVSFLLKQSEMRKVVVHAGSFFAMDLSV
LGSLASAVATYSIVIIQFIVKK
>HhamGR16
MRRSPYKRSVNGVHSIGLKLTVLCQWAWIRKTYIMLGIGFMVFSYAYVAYRKIYHPAVLD
LVSLLILSVGNIIGTFTMSTYKQHEDLYIKVIDYISSKPEANVKFGIMSYTFWILSNLAL
VLNAYLFTTYGSFHDYLLYGLRDVHMCLMTMLTLTVAEITHALKEMFLDIQQKLSKACDH
FLKDYPHKTLFVLKTLATIQQKEEELTTFSNRLRRIIIDHNTLCQNVDEFNTAVGLSNLI
IVVAVVISIIWPITIFIQYNLPNLIHKNDGLDILEISAHILWILLTIHTDEAVFVVWHKN
MDEIRCLLTLLAKQADTRCPILSAYGLFSINLGILGAMATNATAFIIIVVQFILNNKH
>HhamGR17
MSRLFGVITPLNSNTKLKNVENVVPVPTYDKTDPWELKMLYAVSKCKRSLTFFRIVFIGT
NFVTFLYIAIGKTMLYNQQEFFTNLIVLIMSELSDLVLLIVMSVGAFKLANGTELLDVFR
FFHSNRHGTKQLKACASIHILRFLPLLPLGAQMYFLALRIGWQYFLPPDLWFYSLNWMIC
SSACIIAKFKTHFSTTNDHLEDLVTAYENHSPFTEEKLKYIILHYNKLCNNLDKFNDHIK
LFLVVSFASIITNILSNCTAIIEYGIMRKTVKGMTAQVGVLIVQTLLILTSFSQAGLCAM
LGENLEKEGEKTMNICYSFVDRTLGKTLKANSDHDKRLVNLINRLLELTKSRKICVHAGG
FFRLNWSILGSIVSTVATYSIVIVQFIFK
>HhamGR18
MLQADITPNSLNFLIKLGQSLLFLPIGFRNGRFSKFMWLSKRAMAANFLTAISSLQFILY
CNYFFKLENQLPASSELIFYFSALVYKILLINVAKSYAILMEKWQQIDTLMENYTETNNF
RLKIKLVSLLLFLLALGDHIFFIASLGIFFNSMSIKDEIEEYFATIFEYFFIVVPYNGVL
AIFLQLLNWLWTILWNYVDVFLMALCLCFTFRLGQIEQRVNILIENKVEEVYYWKQIRKH
YVQLTELCLELENCIAQLILASFFGKFYMALYRLLGLLDGEKYGIQSDSTKLFNKLYYGF
SLLFVLLRMMTVTICASTIDLKNKDIMFKLFAVSSDIYNIEIERFIMHIHVFPPTITAKL
FEMKRSLVFKIASSIIIYELVIIKFYKY
>HhamGR19
MFKNSKVIPNTTTQKAFKKRVYKMPKAYVRDIFLLLEKKRVIYPLTATLLLLALIGHIYS
LIGKPVIFHNQHVAVKTLEHVADFFLTASIVIIVLHLPKNSKWSFQFFTSLYPEGNKLFG
PLLAMKKHHFWRNLFICNVCIGSLIACEASFFLLHYGWNVYKYLIFRSLEFYFYNLILML
LLTLTRKLELRFAELNELLEYKTEHFLNNRGNNKCDIQMETHLSFILNSEHFYSVRTFRV
MHYELCKLVKRFNEMFGRIILSTVVFSIVNILAKVTFIIDFYITERSDEDINKWSTFIGI
LCFWIFVNLSQSFLFSFYGAQITKEGEKTTRICFYLLNKVSCQTKNLDEKLVQEELMAFA
FQSYCHIPSLSAGGFFDINFRVLGLMISSVTSYLMVIIQFLLRTQFPQ
>HhamGR20a
MFFSKVIFDNLNEVVLSIIHFLMIAPKRTEKYDIFLYAKVVVCCITNIMLFFTSFYLRQY
FKVIKSAIFLPLCNDFSQVLACINILITYRLYLKQKVWQRFYKHYNFVKVHERMQRKVLL

TIIINVTNITYSFYLNYLNAGVVYAGSLNIWLAITSFISSTIQTLYWTMEVTFMIMVVLG
IGNKVIETRKYLKSRPEDIDSHKKSFSIVYEGIEIFGELCGYQFLTITCDLIIYVLALLM
ILIEVVKPLGRLTSEAVIATIIVTGIIVIFSSFGPCAVAFACDMVATEADKLMAACYAAQ
EKFHYNSREYQELQSLGSILGSGVLQFTAAKFMEIKRSTLLSIMAAATTYFIALVQFY
>HhamGR20b
MNKFVRYLNKTFIKCVRVMLIAPKDQKKTCLLLVYAVMVVTLFATSIYLRYKYDSKDLSQ
EILIMYGLYGIAELCGCLTVLIILYTYRKTHLWQRLYTNVNILDEQQCHTVIIVAFFVQV
SVFFYVIMQWNVRLTNLPEIFFVILIDTPVFILFIYHLAVHTLYITIVLDIKQKLIIVKE
QLNSDLITMDIATMYASICQAIEGGKTFNCLFGYQLLIYYFQWFVFLVQVLINIVLMMNP
KKYNYPTNILENISLFACELGLITFGPCAVAFACDMVATEADKLMAACYAAQEKFHYNSR
EYQELQSLGSILGSGVLQFTAAKFMEIKRSTLLSIMAAATTYFIALVQFY
>HhamGR21a
MFPKVKLDYLNEVALNIIYFMLIAPKRTEKFNTFLYAKVIVCGFANIIVFSISFYIGGFF
SSITTITFLPLCQDFCQVVACTNILITYRLYLKQNVWQKLYKHYNFTKVHDRMQLKVFLT
IMVTVICILCNFFLILLNAKILYEGNLKLWLVIAFCITAAIQSLYWTMEVVFMIIVILEI
GNKVIETRKYLKSRPEDIDRHKKSFSIVYEGIEIFGELCGYQFLTITCSLIVYILVLLIF
LIEMVKPTSKFPSELFIAVIIVMGIVVTLTSFGLCAIAFVCDMVATEADKLMAACYAAQE
KFHYNSREYQELQSLGSILGSGVLKFTAAKFVEIKRSTILSIMAAATTYFIAIVQFY
>HhamGR21b
MNKFIRYLNKTYIKCLRVLLITPKNQKKTCLLLVYAVVIVVLFTTSICLRYEYNYKNFGQ
EILIMYGLYGVADLCECLAVLIILYTYRKTHLWQRLYKNINVLDEQECHATLIVAFFVQV
LLIFYAIIDWNIRFTSLPEIFFVILTAMSSSVLFIYHLAVHTLYMSIVLEIKQKLIIAKE
QLNSDLITMDVTDMYDSIYQAIEGGKIFNRLFGYQLLIFYFQWFVFSLNGLLNIVLMMNP
EKYNYPSNMLENISLFICDVGLITFGLCAIAFVCDMVATEADKLMAACYAAQEKFHYNSR
EYQELQSLGSILGSGVLKFTAAKFVEIKRSTILSIMAAATTYFIAIVQFY
>HhamGR22a
MVFSKVKLDYLNEVVLNIIHFLMIAPKPTEKYDIFLYAKVNVCCIANIVLFSSSFYFRQN
VMTSLIFLPLCHEFTQVLTCINILITYRLYLKQNVWQKLYKHYDFVKVHERMQRKVFLTI
IISILCSICNFHMNYLEARLVYAGIQNLWLAVVYFAFLNFQILFHNMQLTFMIIVVLEIG
NKVIETRKYLKSRPEDIDSHKKSFSIVYEGIEIFGELCGYQFLTITCDLIINILGLLMLL
IEMVKPQGNLIPEFHNEMVAIMGIILTFLSFGPCALAFACDMVATEIDNLMTACYAAQEK
FHYNSREYQELQSLSSILGSGVLKFTAAKFVEIKRSTILSIMAAATTYFIAIVQFY
>HhamGR22b
MNKFVRYLNKIFIKCLLLLLIAPKDQKKTRLLLVYAVMVVTLFATSIYLRYEYDSKGLNK
KILILYGLHETTQLCEFLTVLIILYSYRKTHLWQRLYTNVNILDEQQCHTVIIVAFFVQI
FLYIGAIILFNFLYTNFRKLYMIILANLSNCIHFFYNATLHTLLMSTILEIKRKLVIVNN
QSSSDVKIMDVTTMYASINQAIEAGKAFNRLFGYQLLIYYFRLFMFLLHIQVDIIVFVIN
PETYYYPSTVLGSVCLFTCCIILETFGPCALAFACDMVATEIDNLMTACYAAQEKFHYNS
REYQELQSLSSILGSGVLKFTAAKFVEIKRSTILSIMAAATTYFIAIVQFY
>HhamGR23a
MFFSKVKLDYLNEVVLNIIHFLMIAPKRTEKFNIFSYAKVIVCCIANIILFPVSFYIYQD
FDNFMTPIAFMQLCHNFAHVIVCIHILTTYLFYLKQNVWKKLYKHYDFVKVHERMQCKVF
LTIIVTVICIICTLYLLYLNTRSVYAGIRNVWIPIGYLIISVIQTVHLGMQLIFMIIVVL
EIGNKVIETRKYLKSRPEDIDSHKKSFSIVYEGIEIFGELFGYQFLTMSCGFIIYFLALL
MFLIEVVKPLGMLTSPEHIKIVIVMGIVVTLSSFGPCAIAFACDMVATEADKLMAACYAA
QEKFHYNSREYQELQSLSSILGSGVLKFTAAKFVEIKRSTILSITAAATTYFIAIVQFY
>HhamGR23b
MNKFVRYLNKIFIKCLRLLLIAPKDQKKTCLLLVYAVLVVVLFTTSTYFRYEYDYKNLSQ
GILIMHGLNGIAEFCGFLTVLIILYTYRKTHLWQRLYKIINVLDEQQCHTIVTVAFFVQI
LLISYVTIDWNVRFTSFPEIFLLILTGMSSYILYIYNLAVHTLYMNIVLEINQKLIIVKS
QLNSELITMNVTDMYSFIYQAIEGGKIFNRLFGYQLLIYYFQWFLFSLSGLINIVLIMNP
ENYNYPTNILGNICFFACVVSVITFGPCAIAFACDMVATEADKLMAACYAAQEKFHYNSR
EYQELQSLSSILGSGVLKFTAAKFVEIKRSTILSITAAATTYFIAIVQFY
>HhamGR24
MFPKVKLDYLNEVVLNIIHFLMIAPKRTEKFNIFLYAKVIVCCIVNIILFSTSFCAGQLF
DVMTSITFLPLCHDFTQVLACTNILITYRLYLKQNVWQKLYKHYNFTKVHERMQLKVFLT
IMVTVISIVCNISLFYLNTKLFYTSSLNIWLAIAYFTSSAIQSLYWKIEVAFMIIVVLEI

GNKVIETRKYIKSKPEDIDSHKKSFSIVYEGIEIFGELFGYQFLTMTCLLIIYILVLLII
LIEMVKPLAKFPSEVFIAMIIVMGIVVTLASFGPCAIVFACDMIATEADKLMATCYAAQE
KFHYNSREYQELQSLGSILGSGVLKFTAAKFVEIKKSTILSIMAAATTYFIAIVQFY
>HhamGR25
MFFSKVIFDNLNEVVLSIIHFLMIAPKRTEKFDIFLYIKIIVFGIGHIILFSTSFYLHQY
INDYGTSVTFIQLCHVFAKVIVCINILITYRLYLKQNVWQNLYKHYNFVKVHERMQLKVF
LTIIISILCITCSFHMSYLDAKLVYAGIRNLWLPIVYFMTSVIQNLYMTVQLIFMIIVVL
EIGNKVIETRKYLKSRPEDIVRHKKSFSIVYEGMENFGELYGYQFLTMTCVFIISFLALL
MFLIEVVKPLGMLTSPEHIEAVIVMGIVVTLSSFGPCALAFACDMVATEADKLMAACYAA
QEKFNFNSREYQELQSLGSILGSGVLKFTAAKFVEIKRSTILSIMAAATTYFIAIVQFY
>HhamGR26
MAISLQVKKLFQMFLLVCLFPVRYNKCVCEYNVLPLKGPYGVSLGVLTTIHIILIVILTE
HFGGNFHNENFKLSIKLFQQLLSLVIVWSCYFVKKTLKDTMNLFATIDDIFQKSGVNVSF
KLLFYFLSVKLFIVYAAIITINIVCYIYNDSGVVCVLYTFEHIYNYHILAQISMTMGSLF
FLAYQRMVILNFSAIDIVLKGRFTRVFSTEKIYYNKQAPQNTLRLLSVLHFKVVKASNMA
NKSYNFQLLLILGFCLLRMIETVHDLLNRNQTNSIIFLVESFWLVLIGIEFFILSYKATK
LQDMADRTAKIANVIFCNDNILLQRERDIFLLQSLHQRISLDVGGVFALHNSTYLKLWGS
AITFIIVMQGLNAY
>HhamGR27
MQWYSKRSVLCLKPIYYYLSLMSVVPFFDFDKMTTKLKKKHLIYYISLGPWGAFLTVLAF
KEILQVEQYRNSIIFESLLLIFSSICTCVGMTVSILRRDIWKQLIQELSLIENIQNLNTF
EEKESSFAKTVTLSNLFFFHVYRSMYTINIGYIIFNANILFNIIEDKYKGIENLLLQSKG
PMNTILIRKSRILYDRMQKVSRILNKLIGIPIFCIFFYCGYKAVYISAFNYKTYMKRRGL
DTKQLATDIIINGIIVIIPLFVLFFAHQTNSQVTKLQNTCAKLMEKLNEESERYLELNDF
YEQMKHQRVKFTAAKFFDLERSTIIRYLDVISTYFIAVIQFDISKK
>HhamGR28
MAQEIFTLSVDEDELHKGGENNIQADLRNFKYLQKQGRYMFLVPWESKRNFTYRLLGIVI
TLILIPIAYEIILLRLSYFRHLPITCLIMDTIHFSASLAIFFATQNFYEKMWQCLEARII
KTEFLLRTLNYVAPKGRFSLKFRIIHLLYFLIHGSQIYMWIKLYQINLLFSYTSFRVSHY
WALFSTFLIYEWIKFLSRRFKFLDEYLGKQFSKRVIVLHETAKELRSIGKIYKNLSDLVH
QSNKIFFYHLTSLTVLSLLITLHGLSFAVVQFYNVRSSLAALGFVTAYFINILVLVLAYE
DLKNTSQNIVKTCLFLEEGMEHTHLRAEMERLRKYVKTLGPKLSVIGYYEMDFQLISSYI
SFIFTYLIIIMQFNMTFV
>HhamGR29
MKQTSNTMPFENKIVRGIIGLSQFWGILPYFFHISHWTRHLLNMKTKLLMLCFTISCLIT
LYINLFNLNRFKHDNIDSLWIIRFTTSYLAPFAYIHHIFFPKHWNGLLKIVRESKKLMDY
QKYRKVIYFNLLFLLIFHLFFIEMCRARIYNSAIVPVLFFVILGCELYYDFVGSITFSII
VILGVEQKLLEMLRILRELHKKKIISMRSIKELKRLFMSALENHQIINDYFCLKLLISYS
IFFGKCLSGLLLIAYYSKPKPESSIRNEIIFNLNSIGRWFRFIFINGFSFISVLIITMTC
KQVSKSVTNVIMNCYLLQSKIRYNSYEYKEIRALWTFMFENQLSFSAAGFFDVHPSILLS
ISASTVTYFLVLIQLN
>HhamGR30
MVANLKLLKIFISVIAVCPSTLHKRKLVQLMNQYYIVIWMCIVLYFLIFRGSFLMLKTLN
FQILNVACRICEFALTITLTSVITKDQSKTLKLVMRLEDIWKRLKCDSKLFIHFGKFFVI
WLGIVVLKCTITLYLNNYSLSIFIITCIVYIRISWFWTTIVLTLFEFSNQWKMFNRKLKF
VTKLTDVNELQATQTKIFHVIDDFNDIFGKIIFWTILTGTVLVINNICQLFGFHKTIGSN
QHWMLTVDVFLVQMYVVAMVLLADRLALETEKTIEICHKKSQDVCIADRKQNLFIKIALQ
THFKNMKIHACGFFDVDNSTLVFIICTVCTYVTVIFQTISS
>HhamGR31
MALTTIKKFVYTEYNGDRIIFLKSVFKCLRFVGLVPNYDFQQKQIFNSHLYLYLGMVISI
CNCYIVIKAVLFYQNLNMLNLLKVSFILITFCQICFYTLSVLQTWFHADQWESYFKNILD
IEKNVKEISELRTEPQKYLFIFNRAFIGSFLIILSCLEFIVDIIMNCNYYSFDCIIIFPS
WISFIQMIGIIYLVSTIALYYKHNLEKITETLEASLYIMDSGKTIVTSRNIYLNIVDNVD
LFNCLFGRHMFYVMAMSTIQCLAIWLLSLNIKNNEFSNTIYTYYSNIIFNLEAIFFPYVM
TLCCDLTQGAGQRLLVTCYSIQNAFKTTSEEHKELELFANRIINRKIVFRAANFFDLDRR
TMLAIFFNVTTYFIALTQVESIKQMGKL
>HhamGR32

MAQIFSKKEGILTRLFKHKLVSNVAIGLIIIFHFSILIYSGNGKLQLYKKANRLTENGVI
LFVNILRNILLATMNTIGTIWIVKNYISSLPIANCRTRLLGRVTFFQMTGLVPLVFCIYF
IGDQVGWSIYKYFVPEELWFFVMHLMICKYIVVASRIKKNFNGVNRRLAKFMNNDDSSES
YSINIITFKNHQVMYDRIQQLNYRFNEFCNVLDKFNELFKGVVVMGMICIMMNILYNCTM
LIEHIGNPYYRYKVKLAAAVSQPLMAIVSGQAAAIAFIGESLQEEGEHTIRLCFMILNKL
NKNLQWDSNLSTKEQIRFLLNQSKSRKVCLHAGGFFKLNWGILGAITTTVATYSIVIIQF
LLKY
>HhamGR33
MASNCASLIVSGLNIFFILLPSTKGTLPKSHFLVQKVSCWSFLLITVVMAPLTILIKDDI
FCKVPLIHRVVYDCSVMVLFATNIYLSFLSIYKMKELAKLLQHISKNIYMSTDFLMHFSI
FVLLHLVNTLLILCNQLQRTQDLRYQLTTIFVDGYLTFRLILALFMIFLMLSDIRKKWQN
LNGNLTKQETVQKFQYHHNQLCDYVMHLNVLFGMVFLLIIFYFITASLRHAVFVSVSTQT
SVTTVRCLWIVSYTLQIAYLANAGEKITSEAQCTVAICYKRLSKVRHEKWVWLAQQAYMR
TPKISAAGFFNVDNSMILFMATTFLTYFIVVVQLLVK
>HhamGR34
MTVSYVKVFPNKKNELIFETSKKIKEPSLCPCKILRMAFLLNRFTFNFPFTWKHVNGRCT
FKLSTPWRIYYLIGTIVLSVWASFVMPSIKLDVTNLPLFLDSVTNLLSMIFVCVSSLYMI
NRRVWIDFIKRLDYLNKAHLFCFKSKNNIRKYHRFFLAILLGLSVLQNLTFIYFKLFVTP
ELPYHVLFFRNLQVLPQFSFQIWFIYIQSQLTCFENVIVNYLNYRPIHPHKEIKITQNDY
KIGGISTGMSICDEKHYLPLHLSNLSKPEIIEYLRIKHQRIYEEIHLLLKISSPQLLAYL
LIEVLIQILHLYSIIVHIIYKFDNPTAGSTIILNCISIVWHGNGVYYLCWNMNILNEMVE
RLRRILTLCKFTTEDAEEHRQFRIFINKLGHQKSILINDYFEIDLGIIGPVVGNILTYVL
VALQFKIPPQKTN
>HhamGR35
MPFEDKIVRGIFALSRFWGILPHFDFHISHCTSHLLKAKNRLLMLFFFICSIFSLYTIWT
TENIFSSGRQGSIIILNFLSSYLLPLTYIPHIFFPKQQWNRLLKIVKEYKKLMDCEEHRN
LLYWYILILVLYHAAVVGLCIFNMHYNSIIEMLLHIVGVFGIYYEFVGAITFSVLVMLGV
DGKLVDMLRILQDLHNKKIISLRSIKELKGLFITIFENHQLINDYFWVKLLISYGSFFGR
FLCGLLMMANTWKPKVDSFKTKPVDNWDDINGWVMLIFLGFVSSVIVIITMICKQVSQTV
TKVIISSYLLQSNIHFKSYEYKELKALKHFISENQLSFTAAGFFDIHPSILLSISASSVT
YFLVLVQVK
>HhamGR36
MPFEDNIVRGIFALSQFWGILPHFDLYISQCTSHLLNAKNKLLMLCFIIGPLYSLYLILA
TQNATKSGRKGSIEIIRFAIGKLAPLTIIRQIFFSKQYWNEVLKIVSESKKLMDYQNYRK
LIHIYLLLLVILHAILIEIYVSQSYTESSMRLILSIIGGCGFYYEFLGAITFSIIVILGV
EQKLLEMLRILRELHKKKLISMGSIKELKILFMSALANHQLINDYFCLNLLASYGVFFGR
CLSGLLLIAYYWTSILETNIRNESMYDLISFEKWFKFMFISIFSSISVVIITMTCKRVSQ
NVTNVIMNCYLLQSKIRYNSYLTFNYETPCIHEAKVI
>HhamGR37
MPFEDKIVRGIFAWSQFLGILLLFDFHVPHWVGHLLKTKNKLLMLWFIISSLVTIYVSLI
IHNRLRSGRIDCLKFLRFINSHLAPFAYISQIFFPKQYWNGLLKMITECKKLIDYDKCKN
IIHINLLLIAIFHVAIVKVYFSKFYDGLNWSTLLYSTSIFAIYYEFIGSITFSMIVTLGV
ELKLLEMLRILRELHKKGLISMGSIKELKILFMSALANHQLINDYFSVKFLLSYGTFFGR
FLTGLILIENSWKSKLKSSIQRESKFNLNSHDGWIRFIFFNVFALISVVVITMACKRVSQ
NVMNVIMNCYLLQSKIRYNSYEYKEIRALWTFIFENQLTFTAAGFFDVHPSILLSISASS
VTYFLVLIQFE
>HhamGR38
MPFEDKIVRGIFALSQFWGILPHSDFYIPHYTGYLLNAKNKLLMLCFIIGFILALYVSFT
YDFGWNIGSNNDLWQIRFISNYLSLFAYIPKVFFPKQHWNGLLKIVKESKKVMDYEKYRK
FVYFYLLLLVIFHIIFITACVLKFNNNPIIKTTLSLISGMGVYYEFVGAMTFSIMVILGV
EQKLLEMLRILRELHKKKIISMRSIKELKRLFISLFENHQLINDYFCIKLLFSYGCFLED
ISVVVIAMTCKQVSQHVTNVIMNCYLLQSKIRYNSYEYKEIRALWMFIFENRLSFTAAGF
FDVQPSILLSIFASSITYFLVLV
>HhamGR39
MPFEDKIVRSIIALSQFWGILPHIDFHISHCTSHLLKAKNKLLMLWFIICTILSLHLTLT
TQNTFNSRRKQSMEILGWFISHLLPLACIPQIFFPKQHWNKLLKIVKESKKLMDYQEYRK
LIYFYLLLLVIFHATIARVCVSKFKHDSIVIIILDVMSVFGIYYEYVDAITFSMIVILGV

EQKLLEMLRILREFHKNKIISMKSIKKLKRLFISVFENHQLINDYFCLKLLLNFGCFFGR
FLSGLLLIEKFICVCVFSSISVVIITMTCKRVSQNVMNVIMNCYLLQSKIRFNSYEYKEI
RALWGFIFENNLTFTAAGFFDIQPSILLRISASTVTYFLVLIQFN
>HhamGR40
MPFEDKIVRGIIGLSQFWGILPHFKFHIPHWTGLLLRTKSKLLLLWFIICPLISLYMFVV
NQDKLPSGGIDFLKISRFITSHLAPFAYIPQIFFPKQHWNRLLEIVRESKKLMDYQKYKK
DIQFHLLLLVIFHTFIVKLCFLKFDDDSIWGILIHITSICGIYYEFIGSITFSMIVTLGV
ELKLLEMLRILRKLHKKKLISMGSIKKLKRLFITIFENHQLINDYFCLKLLASYGIFFGK
CLTILLLIANLWLRLVIITAISLISVVIITMTCKRVSQNVTNVIMNCYLLQSKIRYNSYE
YKEIRALWGFIFENNLTFTAAGLFDVHPSILLSISASSVTYFLVLL
>HhamGR41
MKRIMSKNKRCRVDFSIIIIWYNVAKLLSFTPISIPLHGGITKKFKLIPYLYPSILLIVY
ITMTIHSFHTRFTTIYISFNISQKILDILQAITESCFITHVVLSSILKKSLWLKLRDNLT
RTERKFSDIMTLSPEQQMPDASLALIRVRIIIFHMGYVFAHLYDSITYWTWASYTLTFII
FRITTYYIMFSTLFIIFLCQWLEHRYIYLNDLLKNSIKDHAWPAGFLLLPSEIGRQQNLQ
EFSRHYRNLYLIVDNVNGVFNTCFFFITICTALEILNAVNYGIPTSTKLDYIEILDHVIY
LVLYMSCYIHLVTACERVRKSAATIGHTSLFLLTKIDMPHIKQELLTLNGYIQGKRYG
>HhamGR42
MSLIQSSKKQIASFFRYCLCLNPPLRNSRLNYIYYFYITLAFFCYAYIEIQYVYQSIISN
SINLSLKISRILLVSYSIIVKVYLTFILIYKRKNVQNLKITIKSMDRVENKHKENYSMKI
YKLICRISLIFRAIFTFKFINDLSFSKWLILFVNEATSFRIECTILELTDLLYVLKKTVR
LINKQLPGICVLGTLKKFRRLSNVLLNCMGTFEKTYGGFIACFVISTITLCLFCILVYFN
RTSYTNVGLIVWTFCRFFTIIVSIIIEVSTDIRQILILNEYDALVNEYKQIMEEIHQRQP
LLNAGSFFTVDHSMILFVLTNIVNYFITAYQFMF
>HhamGR43
MNKLVRFLNKTFIQCVRVLLIAPNKQKNTCLLLIYALVVLVLLSISLYCRLRNFHQYSNV
KIAIADNSSNVTQACGCLTVLITLYTYKKTHLQKLYKNVNILDNDEQQQCFTIIVVGFFV
QILLNSYKTFMIYFLNFIHDSIDINNFHFLILLNLSYCIHFIYKAAVCTLLMILLFELKR
KLVMANEQLNSDLISIDVTAIHTYVNQAIEGGKTFNRLFGYQLLIFYFQWFMFIMMNHIC
IKAFIHPEKYGYIHHINILEKLILLAFESILIAFNPCAVVFVCDMVAAEADKFMAACYEI
EEKFHYSSHEYHELQTLICILANGVLQFTAAKFIEIKRSMMLSLIVSATTYFIALEQYF
>HhamGR44
MNKFVRFLNKIFIRCACVLLVVPKNRKNTCLLLFYVLVLLALLSTSFYFRYACLYQDLTL
GRLIIYILCDVTQTCGCLAVLNILYTYRKTHLWQKLYENVNVLEYNEQQDHAIIIIITFFV
QIVLYLYKVFMIYLLWYMSISVHNLHFLLLMWLSYFINFIYKTVVYTLLTIIILDIKRKL
MVANEQLNSDLISMDVTAIHAYVNQAIKGGKTFNRLFGYQLLIFNFQWFLFFLVILLNII
VIINPEKYDYPVNLLENLNIFACEVILMTFNPCVLAFACDMVATEADTFMVTCYEIEEKF
HYSSREYHKLQTLTSIVGNGVLQFTAAKFIEIKRSMMLSLIASATTYFIALVEFY
>HhamGR45
MKQISKKMPFEDKIVRGIFALSQFWGILHLFDFHIPYWIGHLLKTTNKLLLLCCIINPLL
TLYLIWNTQNGTPSGSHELLWIIRIISAHIPPFAYTRQIFLPKQHWNGLLKIVRESKNLM
DYQKYRKLLYFYLLLLVIFHVAFIRVAVSKFYNNSIMEIFISTINGCGTYYDFVGSITFS
MMVILGVEQKLLEMLRILRELDKKKIILMGSIKELKILFMSALTNHQLINDYFCVKFLFS
YAIFFGRLLSGLLLIANLWKPKVFNIRHEIIFNLNFNDTCFRIMYVGVFTSISVVIITMT
CKRVSQNVTNVIMNCYLLQSKVRYNSYEYKEIRGLWGFIFENNLTFTAAGFFDLHPSILL
SIFASIVTYFLVLI
>HhamGR46
MKQISNKMPFEDNIVRGIIALSQFWGILPHFDFHIPHCTGYLLKAKNRFIMLCFITSPIC
SLYLVIVHQNAVKTKTHEVLWFLRFMFSYLAPFAYIPRIFFPKQHWNGLLKIVSESKKLM
NYQKYRKFIYFYLLLLVIFHVILIKVCMTRIYTFKSIIIEILSVTENCTICYELIGAIIF
SIIVTLGVELKLLEMLRILRELHKKKIISMGSIKELKRLFMSVFENYQLINDYFSVKFLL
CYASFFGKCLSGLLLIENSWTSKLQSNIRNEPKYIISSNRWFRFIFMTIFSFISVVIITM
TCKRVSQNVMNVIMNCYLLQSKIRYNSYEYKEIRALWTFMFENQLSFSAAGFFDVHPSIL
LSISASSVTYFLVLLQFNFSLL
>HhamGR47
MPFEDKIVRSIIALSQFWGILPHFDFHIPHCTSHLLKAKNKLLMLFLIFCSIISLCIILN
PQNRLQSARNESTMVLRYITGHLAPLAYTPQIFFPKQHWNRLLEIVRESKKPMIYKKYRK

VIHCYLLLLIIFHAFVISTLVSKFNDYSNIPIIARIVGGYGIYYEFVGAITFSMIVIFGV
EQKLLEMLRILRELHKKRIISMKSIKELKRLFITIFENHQLINNYFCLKLLFSYGSFFGR
FLTGLLLIENSWKPKLESNIQNESLYDLISFEKWVKFMFVSVFCSISVVIITMTCKRVSQ
NVTNAIMNCYLLQSKVRYNSYEYKEIKAFRTFIFENQLSFNAAGFFDVHPSILLSISASS
VTYFLVLLQFN
>HhamGR48
MNKFVRFLNKIFIQCVRGLLIAPKKQKNTCLLLVYALVVVVLFSTSFYCHYIILYEDLTL
GMLIIIVLINVTEICGCLAALIILYTYRKIHLWQKLYQNVNILNNDEEQCHTISIVAFFI
QILLYFYSLIMWHWLDEPIDNLLLLILINLSPSLQFIYNAAAHTLLMSIILEIKRKLIMA
NEQLNSDLISMDVTAIHTYVYQAIEGGKTVNRLFGYQLLIFYFQWFVSLVINLVNILVMI
NPDKYDYPSNTLENINIFTCDVFLAAFGPCTIAFACDMVATEADNFMVYCYEIEEKFQYS
SREYHELQSLTSILGNGVLQFTAAKFIEIKRPMMLSIMASATTYFIALVQFY
>HhamGR49
MCSCSVNCTKKEKNTCLLLIYALVILVLFLISFYFRFINTYHDLPLGMIIMIVIVDVIEI
CGCLDILIILYTYRKTHLWQKFYQNVNILNNDEQQCHTIIIAALFFQILLYFYTTIMWQL
LFEHDRNNVLLAILRTLTPALQFIYKAVVHTLLTIMILEIKRKLIMAGEQLHSDLVSMDV
TVIHTYVYQAIEGGNIFNYLFGYYLLIFYFQWFLFVVINLINVIVIINPEKYDYPANILD
NINMFTCNLFLAMFGPCTIACLCDMVAAEADKFVESCYEIEEEFHYNSREYHQLQSLTSI
LGNEVLQFTAAKFIEIKMSMMLTIMASATTYFLALVQFF
>HhamGR50
MSIILRKSSQIVLMFLMLYPPHLIKRKIFQIYYSVYIIIGITVLVSLSLYGAFVVTIESH
MIIFLRKILGIGENIVTLVITLLATNTLREQHKIDSLLIFFRNVYQYENRWIKSPGRKIK
IKLVVITWVVVAVIKYLVIKIYMCSPLICLRQIIHYRNVVFLFVICLLLYELKLKFMALN
SRLKKIKKVEELITLQYFQKHLFMVMDNINTMYGLFILFTILGVIILILLNIITILSRNF
TATNGRMMPVFINGLYIQLHVFIIIVLANNLSEEIHNTIDVCYNMAAKCDSSLEIQKKNL
YITMAERCHIRNIQLNAAGFFVIDNSAFIFIVSTVATYIIAILQIQAI
>HhamGR51
MSIILRKSSQIILMFLILYPPHLIKRKIFQIYYSVYIIIGITMLILWYLHGICVGLLEVN
LVTIVRKIVASCESVITLIITVLATNSLKEQHKIDSLLMFFRNVYQFDSRWIKSPGRRIK
MKLIIITWVVVVVIKFVVTRVFQCSPIICVRRILHHRNVVFLFVICLLLYELKLKFMALN
SRLKKIKKVEELITLQYFQKHLFMVMDNINTMYGLIIFFAVLGVISLILLNITTALSENY
SATNGNKMPLLINGIYIELHVFIIIVLANNLSEEIHNTDVCYNMAAKCDSSLEIRKKNLF
LTMAERSHIRNVQLNAAGFFVIDNSAFIFIITTIGTYTTAILQTKAV
>HhamGR52
MNKFVRFLNKTFIQCVRILLIAPKKQKNTCLLLVYALVVVVLFSTSFYLRFLDNYYYYDV
TLGVTIININILYDVTEICGCLAVLTILYKCSKTYLWQKLYQNINILDNDEQQCHTILIVAF
FVQILLHILALFLCNEILIIIILINLEPSLQFIYKMAVHTLLTIIILEIKKKLIIVNEELN
SDLISMDVTAIHTYVYQAIEGGKTFNRLFGYYLLIFIFNGLCSFTNIPLIYSKILTFLHF
DPCVLAIVCEMVAKEVDKFIEASYDIEEKFHCSSHEYHELQSLTSLLGNGVLQFTAAKFI
EIKRTLILSIMVSSTAYFIALVQFY
>HhamGR53
MNKFVKYLHKIFIKCLRVLLIVPKNQKKTCLLLIYGAVIVMLSAISFSFRYLLNYKSSSL
GILIMFSLTEAEQFCGCLAVLVILHSYRKTYLWQNLYKNDIILDDEDQRCHTFIIIAFFI
QIFLYLYSLLLWYGMAIRVDHFFVIILLNLSSSIHMLYTVTVHTLYMNIILEIRQKLIKA
NKQLNSNIVTINVTTIHTYLNQAIEGGKIFNRLFGCQILIFYFQLFLVLINILLAAVVTV
DIETFVPFTGLFEIINFFYLALFFGPCAVAFACSMVGAEVDKLLADCYETQENFHYSSYE
YKELQTVILILGSRVLQFTAANFMEIKRSTFLSIMAAVTTYFVALVQFC
>HhamGR54
MKQTGKRFPLEDNIVRGIIALSQFWGILPHFDFYIPHWVGHLLKTKNKLLMLCFIVCSLF
CVYKIAIMQNRLQNENIDFLRAIRFTINHLAPLAYTPQIFFPKQYWNRLLNIDRESKKLM
DYQKYRTLIYMYLLLLVIFHAIIVKICVSQFDDGSVVAIMVNITAQFAVYYEFVGSMTFS
IIVILGVEQKLLEMLKILRELHKKKIISMKSIKELKRSFMSALTNHQLINDYFCPKFLLS
FGSFFGNCLTGLLVIENSWKPKLESSIQSGFKSNLNSGITWFRFLFVSIFASISVVIITM
TCKRVSQNVTNVIMNCYLLQSKIRYNSYEYKEIRALWMFMFENQLSFSAAGFFDVHPSIL
LSIFASSVTYFLVLIQFE
>HhamGR55
MQQRYRVVFKVVVIILLIWGYIYSVEGRVRHIYPHIKVVTLLCDLGTFSFLSASVITFLT

ILHNGKSEITRFQLPFSLNIPDGEKPTYHYFKVFPIALLIGIAPFTTLYSDLVTLKYFFV
MNFDFVIFAYAMDGVTSLLENLANNFAYLNGLLKKSCQNITFQANNHYWNVKNVLIRKET
LTIIKFVAVSHDRLCGCVEEFNAVFGLVIILSILYILLGMMYVMVCLVDSTTYINFISDN
WWLMPAYVVWLIFLIVTTLLLANVGNAIEKEILNTQKTCFDNISHYLQMTTNETSKILVE
LLRLHDQISTAKITVNAAGCFHMNLNLIGTMYSALTTLSVYAVEFTLYYS
>HhamGR56
MSFEDNIVRSLFALSRFWGILPHFEFHIPHYTSHILKAKNNFLMLCFIICTLISLYISRF
ITSHLAPFAYIPQIFFPKQHWNGLLKIVRDYKKLMNYQKNRKLIHIYFLLLVIFHVFIVR
ICILKFDNGSLLATLVNITSICGIYYEFIGSIMFSIIVIPGVEQKLLEMLKILRELHKKK
IISMGSIKELKILFMSALANHQLINDYFCLNLLASYGVFFGKYIGALLMIANSWGLKLES
SIQNESAFKLNTHDKWIRFMFLNVYTLISVVIITMTCKRVSQNVTNVIMNCYLLQSKIRY
NSYEYKEIRALWTFIFENQLSFSAGGFFDIHPSILLSISASSVTYFLVLMQFNYH
>HhamGR57
MFFPKVKLDYLNEVVLNIIHFLMIAPKRTEIYDIFLYAKVIVCCIATIVLFSISFYFREN
VMTSSIFLPLSHDVAQVLACINILITYLLYLKQKVWQKLYKHYDFVKVHERMQCKVFLTI
IISILCITFSVHMNYLHTKAMFAGNRNLWLPFGYFTFSAIQSLYTVMQETFMIIVVLEIG
NKVIETRKYLKSRPEDIDSHKKSFSIVYEGIEIFGELCGYQFLTITCGLVLYILALLVFL
IEVVKSLGRLTAGSNFEPIIVMGIVVTFAS
>HhamGR58
MFFSKVKLDYLNEVVLNIIHFLMIAPKRTEKFNIFSYAKVIVCCIANIILFPVSFYIYQD
FDNFMTPIAFMQLCHNFAHVIVCIHILTTYLFYLKQNVWKKLYKHYDFVKVHERMQCKVF
LTIIVTVICIICTLYLLYLNTRSVYAGIRNVWIPIGYLIISVIQTVHLGMQLIFMIIVVL
EIGNKVIETRKYLKSRPEDIDSHKKSFSIVYEGIEIFGELCGYQFLTITCGLIIYVLALL
MILIEVVKPLGRLTSEA
>HhamGR59
MECEMQVLKDLATFGGKWMLARHSQKTGLQLKIWKVVGTFNILLVSVLSAFCVTSRLIYI
HRENVPKMMLFLLLVKTVAATLLCVISIRNSCFGHIGQFNEIQDGISRNCDLRVFSGATV
SRLYPWLMKLNIVSHVFVKIIFILIYKQENDELFPFLVNCFPEIYAMIVITFQLYMAFAW
IQYLKRRHEYVLKTTRKLKSSRFPNYLVLIHIRRLEKLIRFLYKFFMLSKEMFGITIFLS
TMMCIFGVFYSIAVIIVNGTLETNKNVQICDMFSDCVILMVFIVTITLSLDKLEIIAKKL
PKSIYLVRNQFENEEIRRELRFFAKYSKELSPKFTAGCFKLDRHLLSSSLSTLTTYIIVV
IQFSVTTINTSED
>HhamGR60
MNKFVRYLHKIFIKCLRVLLIMPKNQKNTCLLLVYGAVIVVVFLTSFYFRYTNDYKNTSL
AILIMYTLFEAKELCGSIIALVILYTYRKTHLWQNLYKNDIILDNEDQRCHTFIIIAFFI
QIFLYLYSCLLWSWMTIEVDNLFVIIILNSSSSIHFIYSVAVHTLYMNIILEIRQKLIKA
NKQLNSNFVSINITTIHTYLNQAIEGGKIFNRLFGYQILIFIFNFFYFFSLFSFILIIDP
EKYKFFSGLFENITIYMSNFFGPCAIAFACSMIGAEVDKLMVNCYAVQENFHYYSREHKE
LQSFALILGSGVLQFTAANFMEIKRSTILSITAAATTYFVALVQFY
>HhamGR61
MSMILRKSSQIVLMFLILYPPHLIKKKIFQIYYSVYIIIGMTVLVLLYLYGFCVVAIDVH
LSTIERKVVGSCESIISLIITLLATNTLREQHKIDSLLIFFRKVYQYKNRWIKSPGRKIK
IKLVVVIWIVVAMIKLLVTRVFLCSAFICLRRILHHRNVVFLFVICLLLYELKIEKIKKV
EELITLQYFQKHLFMVMDNINTMYGLIIFFAVLGVIAMILLNILTILAKDYNATNGQMMP
VFINGIYVELHVFIIIVLANNLSEEIHNTIDVCYNMAAKCDSSLEIRKKNLFLTMAERSH
IRNVQLNAAGFFVIDNSAFIFIVTTIGTYTTAILQTEAV
>HhamGR62
MGLKLKWRIFPKVADCLVCAFNVIVKFSLFNSECPHTNPAIAKPRIYLGKSATFSIIVSL
LVASVSWADNLYQWINNEKENDILCMFRFFQQTWTISIVLSLSLNRHKYLIFYNGILKLF
AHRKRYGIATLFTVKDIEKFQYTSFYIFLVSTFSTLYILIINILKADYNIMNLIHACAYF
VNVFSGTISTLHTIIWLKVYQSLFMKMHIEMKRVCEERRKNLSLDLADRLRLYNSFYTAC
VINYENQTLVFTTIPYDVIHFIYVIVALGFSVLYLSFLLIHQDMEVKNGVIGGVLQSAFM
CLTSYYMTERSTYIQNTVSELRIYKCSDNLISYLLKYPITKLNPNEANVVEDLINDLIYY
KPILFPGHIFIIHKKLVPTMISHVITYSLMAVQFNVQQYFY
>HhamIR8a
MQVRYFEDNLSSYEDVKVKLLKVKLLEDEADDHYEQICDALSHGFTLILDFSWTGSEVAQ
EMVANLSLPYLHADVSIAPFLTLLDAYLDKRNSTDVLVIFDKEEYIDQSLYYWLDSARLR

MVMAGNLDKLTGNKIKKIRPIPASFVIMADTINMNKLLLQAINEDLLRLPDRWNLVFTDF
QVQNFNRALVENQSVSLLYLNEQLCKDFVHKDYCPKSFVLIDRFLDFLLLTIKKILNVMS
ESDIEFPQDFQCKQSNFPLETRDKFEETLQSTVEDNSNVLALNEHSFQVKVWGVVEKVAD
DSNFQVVAQYEEGRLNLAPGKRFEPIKAFYRIGITYALPWSYQERNPETDEMVWTGYCVD
FAQKLAEVLNFDFEFVEPSFGTFGEKINGTWNGVVGDLALGDTDIAITAITMTADKEEVI
DFVAPYYEQSGITIVMRKPVRKTSLFKFMTVLKVEVWLSIVAALILTGFMIWFLDKYSPY
SARNNKQAYPYPCREFTLKESFWFALTSFTPQGGGEAPKALSGRTLVAAYWLFVVLMLAT
FTANLAAFLTVERMQAPVQSLEQLARQSRINYTVVQNSETHQYFINMKFAEDTLYRMWKE
LTLNASTDDTRYRVWDYPIREQYGHILLAINDSNPVANASEGFRITDEHLDADFAFIHDS
SEIKYEISKNCNLTEVGEVFAEKPFAVAVQQGSHLQDDLSKAILDLQKDRFFEELQAKYW
NHSAKGDCPSTDDNEGITLESLGGVFIATLFGLALAMITLAGEVLYYRRKAKNDKIKMKK
SKVIPTAVNLEKFNVGKTVTIGHSFKPIDRKSDLTKELENINISHISLYPKSRNRITRMQ
>HhamIR21a
MLMKLTVTIFPLIIFNQILGNVEKRALQKSHEKSRIEKLSKTFLENKVSYSKVPNMSLIS
LLNTIANDYLLDCTTVILYEESTKEEDYVFLKNFLQSYPHIHVHGSISIDYSLQLTNITN
EKDTTCVHFIIFLKDLMKFQNFERKRNEKVVLIAKSTKWRVREYLQSEYSQKIANLLVIV
KSGKLDWKEEEESYTLYTHKLFSDALGSSLPVVIASWFGENFSKNVTLFQKNLFKGFVLN
RQKNQNDEYEYTGIEVKLISLLAQMYNFSTDFKEANDVKTLGSNEAVIEAMKMRSINLGI
GGVYITENRYNSLKFLWHNEDCASFISLASTSLPRYKAIMGPFRWTVWLALIAIYLGSIP
IFSYSDKLSFKYLLKHPSEIENMFWYVFGTFTNCFTFKSKSSWTNAQRNTTKLLIGVYWV
FTIIITACYTGSIIAFVTLPVYPSVIDTVSQLLEEGYDIGMLNKGGWPSWFNNLTDESSN
KLLQKVDYVSDVESGLKNVTKAFFWPYAFLGSREELKYIVKTNFSLEDRKSLLHISQDCF
VTFKVAIVLPMYTVYGEMFENGLQKILQSGLNIKIKSDIEWEMMRSEVGKLLAANSMSGN
LKLTSGEDRALTLTDTQGMFLLLGTGFTLGFLVLLTEVFGNCFRFCKKHKKSTSSASSIA
SNPRFHERQTF
>HhamIR25a
MKNLQKIFYLFTISFTLTNINCQTTQNINVIFANEEGNNVADKAIDVALNYIKKTTKLGV
SVDMKWVIVNRTDSEKVLDALCHVYQQMLDANTLPHLVLDATRAGLASETVKSFTAALGL
PTVSASYGQTGDLRQWRNLEPNEEEYLVQISPPGDIIPEMIRNLVTNQNITNAAILFDDS
FVMDHKYKSLLQNVATRHLIDEISSDVNKIPDQLEDLVKLDLKNFFVLGSLDNLQKVLEA
AEKKNLFNRMYAWHVLTKDPSDLKAPVKNATILFAKPMVNTLFQDRLRNIGTTFQLSSVV
PEIEAAFYFDLALKAVLSVKEMLLDGSWKKNNVTVYRTCDEYEPKNSPKRFNLNLRSYLQ
KESTEPPTYGPFNVGANGQSFMEFSMSLTAVYVRSGASDKSLPLGTWQAGFDNNLTLFNA
KDMKNYTADVVYKVVTIVQKPFIYRDESAEKGFKGYCVDLINEIASTLHFDYTIEVVADG
MFGNMDEKGNWNGIIKDLIDKKADIGLGSLSVMAERENVIDFTVPYYDLVGITILMKLPE
TPTSLFKFLTVLENEVWLCILAAYFFTSFLMWVFDRWSPYSYQNNREKYRDDEEKREFNL
KECLWFCMTSLTPQGGGEAPKNLSGRLVAATWWLFGFIIIASYTANLAAFLTVSRLDTPI
ESLDDLSKQYKIQYAPVNGSSTMTYFQRMADIEGRFYEIWKDMSLNDSLTDVERAKLAVW
DYPVSDKYTKMWQAMIEAELPSNLDAAVERVRNSKSSSEGFAYLGDATDIKYLEMTNCEF
TIVGEEFSRKPYAIAVQQGSPLKDQFNTAILQLLNRRELERFKEKWWNKNPEKKHCEKSD
EQSDGISIQNIGGVFIVIFVGIGLACVTLAFEYWWYKYRKNTNITSVTEAPKLRFRRDIV
KDDTGFVAGHAGNNDRELSLKITKLYNK
>HhamIR40a
VFTLFIYDLVLENFLVGHSNTNQCLAIVNGDEVTPHSLPYMVALESKINGHSVRCGGSLI
KVDQVLTAAFCVYNATTTTITLGAHNLEEKEDTQVTLESSNYTIHEEYNWTSHTNDIAII
RLGQSVNLTNENIDIRLNNGGDLITAIYDFFRGFPERKITIAFERTDPKLIKHLVRKLLS
GRVSVMLFNFSLIDSQEKYFEYLSKQTENHWSITTLFFGSPKLYEHVLLMIHDKNSIRRN
LVYIFHWGRESFNRKHFLRNIHYAMRVYAITNPRNGTFRLYYNQGTSYREHHLEIIKWWN
DDKGLFNHPNLMTQKKSVYKDFHGKVLQVPVLHKPPWHFVRYNSNNESFEVSGGRDDRIL
NLLSKKLNFRYNYSDPPERIQGSVTNDGGSFNGVLGLISQRKADLFIGDMGITSERFGVV
EYSFLTLADSGAFVTHTPSRLNEVLALLRPFQWQVWPFIGVVCLTVGPFLYGIIVLPNVW
HPRFRVRSHARLFFDCTWFTVTILLKQSTFNVILLLFEYSECEFLAGKEPSNTHKARLLI
ILLSISATYVITDMYSANLTSLLARPGREKAINNLYQLRNVMKSKDFKLFVERHSPSFGL
LENGTGIYGQIWELMEQRQSKYVVESVEEGVKLVRDNRNVAVMAGRETLFFDIQRFGAKN
FHLSEKLNTAYSAIALQLGCPFIEEINKILMAIFEAGIITKMTENEYEKLGRQQKIPEAK
IRENEDTTSKIEIKRIKPTEDDKLQPINLKMFQGCFYLLCLGNIFAGLILLTEISLHKYH
LRRRKTRKPRFLLARKLWGRLKYCCRNTRNKLVILYRNFMRDAVILTLDYVE

>HhamIR41a1
MSFPIEILLNILLNTYFSSSRCILVINPNNLSIRANVPTLNLDKSIRIKENLFNYIGCTD
FLLDVAHPVTDLQLFEREIKFHPDRFNNRKYIFISRNPQDFKLFHLQEIHYFNNLLLISC
EKDLFSFWTHKYVGTSGNNDPYLLDKWFINNASFLHNNNLFPDKLTDQQGRTLKVATITY
IPYSIVGNDTYFYMGSQMITMKLFADYHNLIVMPVINEKDYWGELWENWSGNGLMGNVVL
DRADIGGASLYIWEFVYTYLDMSMSTIRTGVSCLVPAPKLLDTWLTPTYAYNPYTWIVVA
VTFVASFLTLYLIMEFQANRRNFFKFQVKLFIRALMIVVKPFIMQNVPKTEVIKGLLGRY
LMGLIFMTTLILSIMYDSGLATTMTIPYYQKPIDTVEDFVDSGLPWTATQIAWIMSIEDA
KEPNMIKLVSKFKVFSEETLRMYSHGDQMAFAFERLPNENYAIGPYIQKDVIENYHMMRH
DIYWAHSCLVLRKHSNLLQILDMFILKVFEAGLINFWQNEAATKYINPYVQKVVKYLNHH
SRQEYVVKLKMMHVQGAFGILIFGSILAFLVFLSELFHYKFYKLDVVVV
>HhamIR41a2
MELLLKILIQQYFSNCMVLVGDGIYEFSPPFGTLISNVNLDIFSDFNLKIPCAHFVVTSS
DPGKTFEIIEKKIRTQSDVLSQRKYILLAPSGSNISQIFQRKAMNFVSNVLVIESQPQRK
ITKNKPFHTIFRIWTHQFVGLKDNNKPYLLDQWFSANSSFLHGKELFCDKLKNLHRKTLR
IGCLIARPYVMEVQDSGKMTGLDIKLITTILEKLNATLEIVSFQKNGLWGHIFPNGTSDG
IKGAIYQDSVDVGIAGFYSNQETTQHFAVSVAYALGEITCLVPKPILASPWMSPYYAFSS
KSWLLLVITYTVLALMVYKIYMYQNEKKSKIEICAVIVKPLISQPIAKKDYYYRPLMSLV
LVATIALTVAYNAGSASAMAKPLHENSIKTVMDFLNSNIPWFSMQAAEWLLTWKNDSQEI
YRRAARRHYEKRPEELRELSKDGRHAFKIEKVHGRHNIDPYIKPDIIEYYQPMKENLHTE
YIAFLLRKHSIYLHMFNSVTNRLMQSGITKYWAQETFDHEEFHIRKYLQTLNGNLREVVI
LKMSHFLGAFSIYLIGNFIALIIFILEIFK
>HhamIR60a
MFKIFFSVGLHIFLSNCFIIHIKGPTHRQYALTCLEQITKQAYSFTTGNLKFHQQFLLTY
VVTDNLNSPSYEVQRDLLQKLHKQQIWSIEILQKYRKNFQSYAENCEDNKQCFKRHVMLH
KTSLYVIIAENLDIFNEKLEILTRTSTFNNDALYIIYFSELTVPFIAEAILSKLFEYTIR
YMVLMVPQNLKIFNFSILRLENKRGVNCFSNLSSNIDVPLRCIEGILTKKTFNIFTEYKV
TNFFKCYRRVQALPYEPFVINKKDGFETEILREIGRVLNVTFKFNVYQNATLHLGNKSSD
GLWTGFLGPIFNLWHLGIGNMPADPTLTEDFSYSVAYEWAQIVYIVPIAALIPRWRTLMS
IFTLQMWAICLSAILGFAVAFFIFKTKTEASLYKNLQSCINAAFQIILAHPVSIQPRSNF
TRIYFIGFAYLSIILNSIYSTSLIYYLQNSIREHQVSSQAEIVKYHLPIGGSPKYKAMFE
DFDNEEMKALRDSYQTVSEDMDSDIYWLTKVATQRNISTLAIKLNVFYMMRRNNRVVTNR
HGHPKVFIISKPIRTQPVAIIMHKGDFMEEAINDVIQKLILEAGLIEKFKTKYTSGFLKT
ATGNDTNDEQEFHEKGEAISTHHLEGAFAIFALGQVVAMIIFLLRFHIGNCIIYFFK
>HhamIR68a
MKCLKYCLLPFVLWNVLLNATANRTIRDDISFSLFETISSETLDLLLQEIIIKTLHSYKC
MAIISDSIYMDIFQKQWFRRFENFLSYILIFVKDSDDLLAPSNDIQMSLGIAKMNGCQMY
IILISNGLQVGRLLKFGDRYRVLNTRYNYIIMFDNRLFEKKLLYLWKRIINVVFIKKYSG
RKTEKNNSKSDWFELTTVPFPINFEDVLIPKRLDIWTKSKFRKASDLFKDKTYDLKNQTL
NIATFSHIPGTVKSSDSTFKQHVRANIKTANNFSFSGTEIEILDTISKVMNFQCGLYEPE
NADIELWGRKGIGFYTGLLGEMTRAKADLALGDLYYIPYILNIMDLSIPYNTECLTFLTP
EALTDISWKTLILPFDSVMWGGVIGCLLVTSAVFYCLAKFHLFKAKIRKLEKKTKGNAEE
RRKRLLKLSIYSQIIKMDYDIKYSLLKEQYKHCKEDHEPKGLYQFSDPKNSILYNFSMLL
LVSLPKLPTGWSLRVFTGWYWLYCLLVVVAYRASLTAILSRPTPRVTIDSLQELIDSKLT
YGGWGEISMEFFKSSNDEFINRIKGDFEVVNNSDNAVSRVADGSFAFYENTNYSLTGKNM
TNNTEKISTPKEINRNLHIMKDCIINMPVSIGLQKNSPIKPRVDKIIRRVLEAGLIEKWL
NDVMQKNLNKLYGALVILAIGYFLGTLTLIAELIYYDHKVSKHP
>HhamIR75a
MRVISLFLIECTVLAIGTSEMNVDSQMNFIFDVVKERKPHNVILMQLCWSKDKVIELERK
LLTNNFKYQFIKNAKSIPYKTHHYTIVGDTECNFHLLYEYAVENFLLPYPNLWIVFGNAT
NLKETNYFIPINSLWIMPTAFGVKTLYKINKKLQDYEETISGKVSDIDIIHNRIDFKKHP
IRVTYVMNNNETFAHFKDYRILGGDINEVSSCCNNKNSFNSRNTTVNNFSKIVYLLYQHI
FLFLNATEIQVFQDTWGFETKDKNRVFAPGLFNDLHSDRSDIAGSVAFTPPNRLKYFTFI
FAPIRGASITMVFRAPPLAYNKNIFLLPFDQFVWISIGVIFLFYNFMLNIVYSVERKREK
SENGNKVGFFDIVMLQLAITCQMDVDWMPKSLSGKLSVFFLLIFYTFLYTAFSAKILLLL
QSNTNAITDIAGLYKAGFDFAIENQPYNIYYVTVPTSRSNEYWRKKIYENKILTPQGPRF
INALTGIQLVRDTYLAFHVASLTVFPEMEKTYTTTQACSVRLVDSFFKSDIPHIALPKNS

SYSKFFLLSYRRLEEIGIHSREHQRCFKQKLHCSGRTNTVVSVGFREVYFPFFIYIAGFL
LSVCIFAMEHLIKMSSKYPINCCKKINILTNQ
>HhamIR75b
MTTLKNSKYDIPVNSYIMVVEPGISTESLLISSLYNLKDKSKYFVNTVALWKAQDGFLYF
HTIIPSRDRWNFHGMPMTVSYVITDNRSSSDSNIYDNRYKLLDKTTVINLVLYNHFVDIW
NMTHKKIYRSDWGLQFDPIKHFYSGMMGDIYNNHADVAGTIQYTPSDRLKYFKYLVSTTR
HIQVCIIYRAPPLAYSENLFVIPFDDQVWIGCGIILTACCIVIWLIMRWESNVRNFKANR
HESLENAPSFLDIVMMNIGALCQMDYFNEPRSIAGKIAIFSLLFSFSYIYTAFCARIVIL
LQSTAGNFRDFSDALYEANMGFGVEKTPYNVYYFTVPNPRINEEWRRRIYETKLAPDGKP
PIFYSSAEGMKLVQNSFFAFHVEQTTAGDLISATFTEQEKCSTRKIPSLFQGDVPYIACN
KNSTLIEFFMIGFFRLFETGVFERESRRRYLKLPKCLGNKGNFFSVGLIECYFAFTIFLI
GLALSLLTFILEIIISKTMMGKLLLRHFHDKRI
>HhamIR75c
SVDTLNRFHARLLEYCRSYHNFSIDFIGVTNSWGYYQPDGTMDGVVGALARKQIDFGYSP
LVIKKERAKFITFGKGTWSLVKLILRMAFVFRNPNLKRSFEIFIKPLSFEVWMCIISSSI
LLILAQKLGVQLDYAMAHLTSKSLDGDTSWSSCILCSLAGFCQQGVFLIPFSLNGRTISL
TILWLGLLIYQFYSASLVSFLLNVPVTPITTVQGILDSDFGIGYENVLYAKSILKATTSN
EGQEIYRRVSVNNESGFLKRDEGMMRVKKGHYAFHVELVTGYPYIDRHFDLPMICELKEI
PLFPRMYMYSGYEKWSSFKDVIDICLQRFEENGVIARELQFWHPRKPKCVRTPATIKINT
SLEEFYPALVIWIFGVAASVGIFLLEMIYHRIKENTTFEFIN
>HhamIR75d
MNYPRIATVFMTSILIANGFIDSDLVLSFFQFHQLTHNIIISCNNQFKLLKNLTKEPNKL
IMVKPVYKVNILNDLVSNYKRIGVILDGNCNETGQLLIRCARYKVFDARHFWLVLFNSEN
FMDIFQKVNLNVDCEVKVALPVPGHNKYLIKTIYNPAYGKGGELKVSKIGIYDIDNGYQV
NGIDNKYFKRRNFTGVHFNSAIVLAEKYERSLENYLITDKDKQVDTLNRYHARLMRHYGT
MDGVVGELERKRADFGSTPLIAKKERVQFITYGRNAWPLRMAFLLRNPNSKKSYQIFTKP
LSFDVWMCIVCSALLLVFVQKLGFKFDHELNKYVDTSWSITTVYTLGAFCQQGITIFPQC
ISGRMAALITLLLGSLIYQFYSAILVSFLLNVPVSVIKTVEEILENGFEIGFENVLYATS
LLKAATSKTSQDLRKILSAHNNSGFLDRDTGMNYVKRGHYAFHVELVTAYPFMEKNFDAS
MICDLKTIPLFQPMYMYANYQKWSPFKDVMDVCLQRLGENGVIARELLFWQPRKPECMRS
ASTITFNTGLEDFYPALVILAMGMTLSMFILFLEILHHRI
>HhamIR75e
MKLLCCWLNLVIFLAFSTADNYQNDSKWMFLKDFTTKYGHLKINFYGCLKQVTKCILERQ
LLKAYPVRYIRSVENLKTSHAELLIADGSNCNLTSYLHKLDEYNMFLIPMKHLILTSDEN
SMVNLFAKLKIPLTGEIILAIFSDESVTLKFLYKIGRNNYLVKEQLGTWTPQSGLHLWPA
NAPLRRTNFQKQPINLGVVIFHEMKVLQSNLDDIPETTEIFFQQDMPLIYTLIQNLNATM
NMIVYDKYGYEVVNKSTGEIVINGMVRDVYDGKLDISESVYWSPARRSRLQALAPIDFWE
IIFFLRKPSLSYITNIYFLTLSRWVWTVTFAITILIITTIYITFKWEKTVIPKRKSVTGS
DMILLSFEALCTSLEPMRIPGRFLLFLFFLMVMFLYVAYSAGILVLLKSTAKINNLNELL
ESRYEVGGWNENFIKEYFTSPKQGVLRKLYVKKIHANSYFTVADGLEKVRKGNFAFFTST
TLAYKYFSKNYTNYEICCLQELPGYLNMKLYPTVPKNSGYAEIFKIGLLKIKEIGLNTRA
KHRVVTKPQCNNQSGIFQSVRIYDCLFVIVLFIVGTSLSVAILILEIYCSVITHLRNKIL
FAVIHPELHCNSILVLSQKAFFLAADGDYELVLGSWDFFKWISYLYIVCDFNLVASNNL
>HhamIR75f
VYTLPFDTKVWLSTFAIVVIMTFVLLLLIKWEYAKQKLTSEIEKKENQIELRESFGDIVL
VTFGAFCQQAAAALPYSTPGRVATLCLFVSLMFLYVSYSANIVALLQTSSNSIKTLEDLL
KSRIPVGVDDTVFNHFFFSTTTEPTRRALYLKKVAPPGKSPNFFKIEDGVKKMRQGLFAF
HMETGAGYKIVSETFKEDEKCGLQEIQFLQVVDPWLAIQKNSSYKELFKIGLRLIAENGI
EQRENNLIYTKKPHCASKGSAFFSVGLVDCYLALVILLSGILGSFIIFIIEIYAHYNLSK
PIHFLEYFRNK
>HhamIR75g
MLATIFLLICLLTVTFQSQSQDRVLLSSLRFYQPSLVVLFTCWKRHDRIRVHKRIISEGL
DVRLVFLDVSLGGGAATAKVNMKEYEKITFFLDASCENSEEILLEASKLDIAGLFQFLYT
WHLLTVNVNSIINIFHKIKTRMDMDVKLYQLSKDDIEILEIYNPGINVGLKIRRLGKLKN
DKIILEPFESYYESRKNMSGVLIRSANVIKYPFTGTFHEYMVDTQLMKHDIYSKFHYQLF
LGLVEIHGFAYNTTLSVSWFGNTSRGEDGGVAKLLWDEDIEISSAGCVIRLLLNDRIDYF
DFIMPYYKFRSCFYFRNPGLVKPNFQEVLKPFAKQTWFVALYVTGIICVCIEISYFIHSN

RTWSSLVSFRSIFYVIAAFSQQSIDVPTQLASRIIFLHLLICSVLLYNYYTSSLVSSLIS
TEPEVMKTIKELVESNLKVGLELQPYLITFILDRVKEDPDIDLLNKTKFYERNELNVFSA
EEGVRRVHEGEFAYHLESVTAYSLISDSFEQESICDLAEIPLIQSDTSLMTKKKCEYKKL
FQISLRKMWQSGIIKKTVQNMGNGQA
>HhamIR75h
MKLRLLVFYISCNVAFEGWHQGIKGDEIPYVKKLEKLNCSTKFSREIIQLIIDYASWRSI
SMLYLLDDSINAGCNMKMLSLTLECFNENSLRIALLKKPPNQCHLTSNIQKTLIVSFVPV
EINLDYYNKQLSLLNNENFAWFHIHLNSNNISDYLLTTKLSNSAISLSSDLVLAIGTEKL
QINHLVSIDNCKRPRYHPVKQMDFPEKKLFLSYNGNITFPQLCGTHNNSLGNSLCMLQLY
KIRASSNSTMIIRPLGYWNYETGTVKLKRYQSVEFRMHFYGTAMVFGRKQKPQDVDDTKE
IDGIIPEADVDVQNLDGIAEYIVSYLNATKEEKNYPNLGLKTSNGNWSGLLGAVVTQEVD
IGLDMSIKSSDFHHDMTFTHNIMVTDRNIYLKPEQSNNARNIFMAPFDPELLMCVLIAGL
IFALVMAIYIVTDMKRHKLHSQTYLTHIFALFDSIFWIVGVFSMQGTQIQPRKFSGNIIV
VVSLMFALIIYNSYSAFITSMLSFKLPSIRTVSDLLKSTYELGYTKNSQDEELLRTMNDT
QLTQIYLRGFLHIQTNPIKNITDGLLRATRGNYGFFATGHLARKEFLRISGYKCKFDITE
ILAPYTRHMVAFPVSKKSPYRKVINLCLIKMKETGVHDYVKSQIAPNLPKCDQQSSYQSA
RINDIATALQLFLLGVLLGLFICLMECSWKNRNPILAKLRKLAIHPNCMDSKECERKWLG
RVRFQDVELFRWFAAFSNSQMVPKKEGQRMKSGLARELSKEEFGLNVESASNMKNGPNSL
VTVESKKKVKSANGHCLLVGPSRPNETFNIKCHYFSRTMGMRMRRKKILSNIRKQAK
>HhamIR75i
LLGTLFFGYGNIKESLKWIPIFRYRVLSNIMTKLNYVILQHLLNLVNATSLEIFKNLDVD
MNKEANSGNFDIAGTPLRIFSENISEFNILGLTYQVDRKFIFRATPHSYTSNVFTLPFDS
YVWYSCFGLMGIIFIIVYLVVYWEWNDPIFKRYIIPNISLRPDPVDVFLMEVGTAAQQGF
EAEPRSNAGKIAFIFTLISFMFLYTSFSASIVALLQSTSENINSVDSLIKSRITIGFKEN
VSMDYFNDSKGLTYSDLQKPFFASLENGVRKLREDFFAFYTDSYEIYHYVNHWFLENEKC
SLREIPIKNTIEHYDLLLRKSYKHKDSLRVGLCTLNERGIRTREYKRLVPRKSICDGIEG
NFESVGLIDSYGAFMGKKILITKTNQLNNIAVMGSGLLCSFILFIIELLWLKYKTALENV
TLNIE
>HhamIR76b
MSLSEILLPTLTTLCLNYTCTDNVNYNEYKRNAPLEKLKEELKNETLVVTTLLNGLLSGY
KNVSGTLVGTGVAFDVLHILQKEYGFNYTIKIPEENVFNEEGNDKGIRNMLLKKVADVAA
AFLPQQYSDEIEYSRNLDTAQWVVLMKRPGESATGAGLLAPFTQQVWGLIIASLLGVGPI
LWVIILLRARMCKDDNDIVFSLPSCMWFVYGALLKQGSTLNPRTDSSRILFSTWWIFITI
LTAFYTANLTAFLTLSKFTLPIEKPGDIIKKHYKWITNKGNGIIEQLSSGNGYAKGLYDE
IGYPVSQIDPDKEDILTKYVTRWEYMYIREKTILETIMYDDYKAKTKANVDEEHRCTYVI
TLFPVCVFARSFAFRPGFKYKPLFDLTIQHLSESGITEFKQRELLPDTTICPLNLRNRER
KLRNSDLKMTYLIVGGGLIISTTIFAIELVIHFTKMHRCCKNRLHPDNGFNRSDTLFTIS
KNYNNKLLSNKKDFVTPPPPYHTLFGQPSVAPGMEYRKRTINGRDYWVINDKFGAKSLIP
QRAPSALLFQFTN
>HhamIR93a
MLLNVVLVLACMEFAKGDVFPSLLKTNATMAVVVDREYLAEFYGNITMEIERYLDYAKRE
FLRHNGLNTQFFAWPAINLKRDLSILLSITSCLETWKLFAGAETENLLHIAISEQDCARL
PPNSAITIPIIERGQETPQLLLDLRTMDIYKWKEIVIMYQDNIPDDLLTRIIKSITRKLA
KTTSSGVSLIKLTTKTFEDINYVYSSIRASMLDINPKTIGGNFLVIVNLDLVEKIMEFAK
ELNLVNTQNQWLYLISNTNNRVFNLKRFKKLLREGDNVSFIYNSTVVNTTCDEGTMVCHI
RESLTGVLKALDDAIVEESEMADQVSVEEYEAIRPTKSERKRYLLEKVQKYLTEYGSCNN
CTKWKLEAGETWGREYQTPGENSIADIQLVGTWRPSDGPSMTDVLFPHIAHGFRSITLPL
VSFHNPPWQILVTNSSGEVIGFQGIVFNIIEELSKNLNFTYSVEVIKNYQAESNNSAFQT
YNNESVDFINMAENSYIKTYGVPQIVLDMVHNKSVALGACAFTITEENKRMINFTDPISI
QTYTFLSARPRELSRALLFISPFAGDTWFCLATTIISMGPILFYIHKMSPVYEYKGIRVK
GGLATIQNCIWYMYGALLQQGGMHLPYADSARIIVGAWWLVVLVIGTTYCGNLVAYLTFP
KIEVPITTLQELIDHKDSVSWSYARQSLFEALIKDSTENVYKTIYEKARDISNKRIMIES
IKAGKHVYIDWKIKLQYLMKQQFVESDVCNFALGIDEFCEERIGLIVSTDTPYLIKINEE
IRKLHQVGLIQKWLEDYLPKRDKCWKKKRSVEVNNHTVNLDDMQGSFFVLLIGKIM
>HhamIR100a
MSLNLRNFLILVHWMVVSSKLTLTDLRGCYCYSFQHFEIYMKHFTLSQHVLITRILDSEV
DYGSADILDKFFEINIQFLDKPIQMKTVHIPGNLSKTTDREIESDLFQFENTTTSTVTFR

QRMLYTDMDLVRIFIGKDPDMLLTYFGTLKIKSFAKFSRCLTVLLFSSLITNKTSIQELL
LFLWKTFSILNVIVHFPCSPETRDYIVTYKPFEMVINGTSCGQMRIFHYSNILAHPELLV
NDVSNLKGCPLKVSLFERSPTALFRLPNHLKHSNIYQKIPFTSTFYGADGIAVSELSLYM
NFTVSLYDDLFTNSYGSTYENGTASGSLKFVVDRIVDLQGNSRFLMRYDVDGYEFTHIYH
FDKLCVVVPKAKKISKWLGTLQILSGKVSICSITVVLLCGILNKFIRSNSVSIYGSIEEV
YSTLLGQSILYRTVKDQLISRRVFLGSFFIYSIVISTTFSAALLSVFTTTKHLPDINNLQ
EFDDSGLTMKSNVNPFKYSDLPLYQRLSKKITRKTTNLSSMEIAATYHFGGFERFMDARL
MIQTKYTDKTGDPLLHIIPECPNSYFLAYIVPIGSPYLKIINKFIIWVNEIGLKTKWFED
FTEGLISDNRLKRLNENVSQESNPYRSFSFEDVKGMLIIILLFGYCVAFFIFLLEISSFRF
K
>HhamIR100b
MWHVKLLLIVFGISWGKLSKPKYPSCFPSPSQLIYEKYVLNATLTNIINLKFKSGRSADL
YSVSGGHRIFQTIQIQDVLVQKFPNFNQTRLNVEIIYMKDDYSIQEPHQMYLYYDQNMVQ
VYRGYDPEVFWQYLHGTDIKEFEDTARSLHALFFLNPETEIRDAAEILTYLWTKFGILNA
IAQIPCSPKYSRYIAVYKPFQLNSRNTLGKVQLHLVEHVFKYPSSIRNNVGNLHKYPVKV
SIFKRYPTALPELPRTLQGLKIYQKNIPFYGMDGMILSEIVQKLNFTLKIVSTPETQRYG
FVAPNGTIIGSLQLIAARKVDFQAVARYIDIYPISMEYSWPITFDYMGFLVARSGRLPNW
LKIYEIFLDNTNLLLTSIWVVCCLVNLLFQPDAGKAFTEIYCIAIGHSQKSIVNPGTSLS
KSIFIGSCLIISLVLITLLTAALIKTLSSESWCPDIQTLEELTQSKIKIKSSANILKFCN
STLYGKLSKQIQIFDSNLSDEDLVTEYKNLAVLVRVKDTQLKIGTHYIDINGTPLLHIIP
DYIANNFVAYAFPKGSPYLLIINNILTRLQEAGLDTKWYKDVAIAFETENRKKESAKIEK
LSIEALQSALYILLIGYLISIMVFSFELAFSCH
>HhamIR101
MPNNFPTIVWSLVVSFSALSLSKLSTFNSLETEIRKSLLNAIFENETFICHIRDTNDIQP
ELLLNGFIKKPSLVLNFDNVTTWSNREDWECNGYIISTTNLQKFWPNININNKLLNNEKM
RILIVSNKSFSSNVVPILGGGDVLGLEIKEIYRILRFESNRLLFEWHSNSSQYRIDKWKF
AKNPKRHPLFNRRNSTFKLAIFNCEPFVHLSPNGTIISGSEINLIKEMTKGMRLEKVFDS
SRNWRNPWLKVFKQVEQGRSDLAGCSQFLKNTLHSNVDVIVIQQQICETFLVPKARLIPA
EYFLIHSFDPYVWLIFSVTILIVVFLSFRFSKIFTITNIIRIYSSGAIHKSPKTIVNYSA
RLYFSSLLWHSFLMATYYSAGLSSSSAIPRLTHQINSFEDMFENHITFQEEDDDIYQSFM
TINSSLFTNLATLFRYGSRLNTTLDGTDALRLKTIENSYVTDLDGFDSDRLRQYKVLKEC
LNNDYICFVLQKNSPFKQKFNNIAQIMAESGILVKWLNDEIRLHRKSQEHYFTSFIVNLD
FATICMEQLSGVFIMICCGHIVACIIFVFEMFRF
>HhamIR102
MTIKCNPTEHKTLKNVFNAIFQNSTFICYIYEQYDILQANNMIKFVKKPIVFIDLSSSTL
DMTAQDCTGYIVNIVNESLIKQHYLDLKPHRRIVIFYQYGKVEPHFPELLGIRAADVLEF
EIPNLRPPFKITTGWNKLIVQTYRVVSLADEQVIFEWNVTDALYQLDERNFWQRHWNPNK
DLFKRHRYVFKFSIFNCKPFVYVNDNGTFGPEFNLAREMTRGFPLKIRFVNRICLNTSNG
ANTNKEKNLDFTCNQNQICNTFIVPMAQPLPETYFLLQPFEKEVWICLGISCLVILISIS
GLRSVLKTIGLDYFANDYGQLLNNIIKLYTAGNVTVQRAAMLSSTIRVYTIVLLFHSFLL
TSYYNAGLSSNMAIPRLTGKITTLQDMVKYQITFQDRPGMKGELLSINSPLFLEIAKLHR
TGSRNKTKLDGTTALTVKTLEHAFVTDLDGFSWEELRRYRALKDCIGNYYMGFSLQKNSP
FKKRFDSVAITLMESGLVTKWFKDEILLYKKMQDNFFLGNVNYVKYTTINGKRIYGGFLM
LLLGYIISGLVFFGELKISKRNARKLTIYPAQSNKIPPKNFVKLIRLK
>HhamIR103
MNLKQIIVLLFMIEVAKWNCVVSPLKTDSQLFKKVIAKLLYGQVCFSQDNEFEDLRASKA
VSIINFDINKFPPWIYCDSYLFFLKEQNLRRISQHLLPHKQVIVVPNNQMNEFNVDSELI
FERAIQMIIIDQRNDSTLNVTWILEKRSELVFDHENIELKQQKWDPKEFFKRTGRKIVVT
TFNCPPFVEIQNNKFEGIEFKIMAELLKEWPVKYEIIEDQHKNKLVNKFLIAMERVKNKK
SDIAFCFLWQRALMERNLDYSSAMFRTCVTFLVHRPVPLSNYTFLFQAFSNMFNFFIFVL
SVLLLESLNKYFLLEKNIEIKKKCENFKIEKLSSKICIISTATFYFLFFSYYSSQLTKLS
SFPKFSPNYINSFNDMVKHEIQWAEPANDIQTWMKATNDSTLVGISKNFKIEEDKDVMNM
KLRMETQAILVKRFTTTLFSGVEDLDDYGRLYLRSLPGCLATFYSSLAFPKHSPFTFHMN
TKLSGIFEGGLSNYWEKITSRKPEHRSMTNFRTLYISQMEIAKFNISKLAGVFYVLIFGY
VLSLLCFIKECLKLH
>HhamIR104
MCYALLSITENRNENLTKCIELKEEYVEHSIHSVSVVSPIQNISENLQYIVQDLAKNKRK

LVSIFSINRIKNMNSFQSTDAVVIEIPNLNYFKNSLKRLQEANILHTHFHLIMPTCFNNS
TLVAKKILKKFIKYSLFNVIFLMRNSDNETYSIYKLSPNKEILIGECNKGNYMFNTTWPI
HEILKNFKFSQITAVYLNIPPYAFDVKTNTIITNEESKWYGIEVTILINIMALLNITVKF
VDGHALGEVFANGSITGSLKLLAQGNAHIAFGGYSQNTYRCMRFHCTFPHYFETLNWAVP
QEYLSRMQLENIAMIIRPMVWPFIIFYMTISTLLIVLIAKNKKLERKNSIFTTYITSILS
SNQLIEKYSNMDDIVYYNLEVYISPNSERFFRNNESKSYFLMQNAKICEVDDYKKCLTKM
VMHKNVSFLGQKGLLDFMKSAFVSKSENILIRVLPPVLSYPINFIMIKEFWGIARVNDLL
LRTSASGLIQKWKKLPRKSRLKECQVKFQDISDVSSLSSIYYGGVMV
>HhamIR105
MYLKIILLIYVKFGHAMNFSEYFSTLKTMEHNKNSIAIVNSHLAQDYLKFLKGVSIEIFN
FDGFRNEFSLKYTTKYEYAIIFIEKFGEITRITQKFLQEPSIWYSNIHYNFIVLNDTIKS
KNLDIHLKLLWNTYRIYHFHLAMYNSTLNDLHVFTYDVFKDILNDFHSSKSIDMNGKAIK
GNVIIVDPTEGEFNNNICVWGSVCIALNAFLKKLNATFKSIETEDTAGIYKFSKQIVVDG
FSDISVGRHWLTTEHLMYASYSIEVSTVVGIVHNAKLRSVLANLYYMFDIYVWSLTAIFK
SLFACLQYIGVKKAIFVNANILSIVTETAFQSAIITALTSPKYEKNINTVEDLCVKGYTA
RGEDDWVFMLNEKLKFEYLSYVEVGDKLWRFKLGSKEVYLSTDYWAEQLLMNRSNSDIFY
SSYYHIIKEPLGMGHLTYYLKRNFQLTQYFNRILLLMHDHGLNSKFAFRKYSFKKDRKVQ
KLNLSHVYSAFLPLVCGLCLSTLAFIIELACQSKKINKSY
>HhamIR106
MFSSKSESKLQYERFPTSNTTLDCITAVVNNLIKDFEDVILTNIWELALGKPLIRFDTQT
FIKCLENKCPIIQLFILDIQNQTINELLKYLDTNSFLNPRAFFIFVHCNIDFYVLSSLSK
AYINKAIIINEFGEIQTYVPYKYENINKPYLTPYKIGYCNDDFNFDFSLIGWKPNTWKNS
ILNVMLRITTPYVTGHNTGIEERLLSLFQDRLKCKMNFTYMEFGTTTLPYFQPKKLLHSH
DADIFGGHITILPSDVLDSDITPPYLYDSIRFVTPKPKEMSFWIRSFTIFPDSFWILLVL
TIAISSVLTNLLYNISYQDKLFAFIEILVEHPVCLGNTQLLSKRIFFMSWFFFSMVISTL
FRNSLIIINATSKKTNAINTLDDVANSKLPIYKTFDLKSYYITREEQEKIQYVSMKQCTN
LALCFRNVAFNQNSITIGGADILNNYVIPLYFVENGETLLHIADEMIYGFHIHLLFSKGH
PLFEQCSTIIISTLSNGWFVKEYKKMKYELSLKLYQKYPIKSKILSMNELSNTFHLWFFG
LLLAFSVLSLEIICYYGLVTFKKKV
>HhamIR107
MCLKILLGLLLLPIIAPINGEINYLQTQLNHFLTRDSLYPAIPLSVGVVHCGKFKETDFV
NNCLKNRHFATQPVIIYCNQHLINETLKFSHVLMFVTGDLFKHFQVFYQQGSLWTSQTRF
TFIFLRDEDDNLMKQVQKEFWNRFQVLNYIFVYVTGNGTFIVQSYNPFNSFFKNFVNDSD
LFEDKLQDLHGYKIKAVLSRSVITNLTQGIIPDDNKLFGYFVAQYLNLTLELIVCDRYNY
QIHADVMNKGQVDLDLNVGNISNVEYGFGIAYNGPLITLLRKPSQYFRFDMYKVFDLHVW
FLFGVSFLILNICKLIALKSKHFPNIFAIDQVVLTFCFVNFSQTVLTSVLALPLTQTYME
TIKDLMKNNITIYMSPNTVAFISERVDRRNIKVIAMADNFNKILDRQLMHKEAVVFWPMS
FEKMKEASPKTIDNYYLLKEAIGILSYGRHLKYPAPLKEGFERAFVRFKETCFSPIFCVH
VKVRMQNEHLNFHTLTVYDILHVFNVLFAGLGCGLICFLSEIIVFQLHNIKCLLHFFKN
>HhamIR108
MYLKILLGLLLLLISTPINGEMNYLQAQLNHFLTHDSLYPAIPLPVGVIHCGKPKETDFV
NNCLKNRHFPRQPVIIYCNRQLINEKFKFSHVLIFLTDNPIENFQVFYQQKSLWNSQTRF
AFIFVIAQDDNLMEQFQKDFWSRFQVLNYIFVYVTENGTFMVHSFNPFNGLFKNLVNNSE
LFENKLRNLYGCKVTALWAPTGMSEITNGMVKDDNELFGYFAAQYLNLTLHMIISERYKP
QVHAELVHKGIVDLDLNLNALRDVKNKFGITYNGPLITLLLKPSKSFQFDIYEVFDLHVW
LLFSATFLILNICKLIALKSKHFPNIFAIDQVILTFCFVNFSQTVVTSVLALPLTQIYME
TLEDLLKNNFTIYMTRNTEPFIDEGMDRRNIKILSLSDNLRKILDRQLLHKEAVVIWPKS
YEKIRASSSRILDDYYVLKEPLGVMIFGRHINSWSPFKVAFEKVCVQLRETCSIPMVCVN
VLLQVRKEDLHFQTLTVYDMFHVFKMLFVGLGCGLICFLGEILTFHLYNMKYQL
>HhamIR109
MVIIVGKTLIRPRILLILLIIPQATCHLNYYEQLEKFLIHSALYPAKRLPFGIIYCSEDL
DFLNSYLKTIRFSVIVSNCNESLQYKLTHVVVFLQKIEDFHKQSCLWNSQTKFVFIFLTP
PLDISRIIKDIWIKFKLLNYIFVYANTKGRLTVSSYNPFTNIVTAKNKYNYNLFEDKLKN
LEGYKIKAVLTPVAITKLTHGLISDDNILFADYFSKYLNVSFEYVIEEKYNQSNHLELIN
EGIVDMDINIGVLNERNIPNSFGVTYKGPLVTLIMKPSDSFTFNMYRIFDLYVWLGFATT
FLIVYIFKHLNYNLFKIFSINEGLLRFFFESLLQIVATAVLAWPTIGIFMESIEDLVKYN
FTLYYSTNSQQYVTPYIDEKQVLYLRTMDILKKIIHHEFLAREAVVIWPITYEKLKMTNP

SDLDEYYLLKEAMGVIVYAKFIERNSPFQNSLEKATQQFTETCIHRLSCLHMMYHTKKET
LDFRRLNVHNSFHLFKLWIGGLGISCLIFFVN
>HhamIR110
MGVLLMDNSLVGNDILTNWLKNLRHIDIEGVQILSKIPVNSTKPAFTDSIIFLEYCQRDY
INSTLSTLTIQAGIWLKDSRYVFVITEICKEDLNSYVKFLWNPLHILNYILVCFTEISLE
PQILAYNPFFDEILNYTDVMPEEYFPNKLQDLNGHNFTISYFNFPPYININPEGEVRGPD
YEFAKTFVKQLNASLKEIRCVDWVDLGQAFSVADLSAVHMFLFEEFLKIVAYPHAFLDVV
IFVKKARPVSALVTVLYMFDVYTWLASLSVLLTFNLVKKIWMKVFLVASRLNQTHLSLRL
LIFSISIFNIIFSQTFQSWIITFLIYPKQGRNINNIDELIESKLPIYSEKTWKAFLTHRL
DRQYIFFDGMKYNITVQIDKFYESSWAYVMTSDIAQTMFNNPLIARYAEDLHWIREPVGT
GYVMFTLGMNAPYKAMLKQYIFKSLAFGYSSRIWGVLPSVSKELSDAVKKLQLSHVSGIF
VLWLIGIVLSITLLIFERMWYRYKRVSEVFDYVN
>HhamIR111
MWLKMVVGYLVVQTVEFYKINISDSSREIHYNFLNCLPKDMLLVILLEDQTEFENLRKAA
SKEQRPFYNFKFQGNDEEFMENLYNLTVKNFILIVDSIEHLNKQVVVKYSFHFTVLQIYH
NLDDSNLHLKISRATFFKTYFVEHSGTVNFFNNCLEITSLLVPPWNTVEKLRLTYFKSSA
AFEDNHVLQGFDVNLMKLLLHYIHMPAVYITPLDRDTFGNNINFSGAFGQVARGEADMAV
NSRIYSANNRLVNYIYPRIRDDVIIIVPKRDVEYNLLNLTKPELGLILAVFVIFLLYTRI
FNNVPLGIILLDIILILTGKPIQITSYLKLKWFRLTYITVMGLAYFAYILFSIQLFSQKA
QKSSTVKVDTMEQVVSSDIKILVRPGLISDVENALQYHPLRKQFLSKVTSFPFGPNELVT
EWQLMVNCDQAVVCRKSTFQRFTKELHRKFRKICYDMVKEPLIPSFNSYIISFENPFFDR
LNKGLIIVSETGLLFNKTALRPEKEITFYDIYGKKFQIADKQFWIYYCTSMAVSVVVFVL
ELLMGLWQFKRISGRKMWHY
>HhamIR112
MRIFGVNILLFMVLQPCNSKIDPPKTSNTLFSSQFIKRALFFIENEKNHVVGIVTRPDKN
YKENDFLTELLLKIHSRKSFGIKIVDNTFKMVSGKEMATFTDSIIVVESFESVNLTKFVN
LLFNQNKLWKCENRYTILVIEMVTPTTGFPIDQLKSLWINSQLLNCLLVFHINSSTSDNN
DFQMLSYNPFSNTIDNHSSNRELSFQDLFPNKIKNLYGYTVIISLCSINPYTYKIEGKYQ
GMDYDFIMAFLKSINATYEIIERKYYAEIRHDLKYSKVHLSGMGLLYIPIDDEIGYPHGI
MELVAMMNKPPMQNHFIALFQVVDRYTWILIIVILSTVFAVRKVFLKLGQQDQKEWTGLN
ILNFNISVFLLIFSDIFQGWITIYLIYPPTEKAINTVDELIESKLPVYSLRSWKDFLTPK
LSGRYIYYSDHGLQLQTSDNSVAAFTVTYDFARFILTSRKYQSIKRDLYTLQGSLGTSFM
AHHLIRNSPYRVALQKATLRGLSYGYYSKEWGVSRKVLNDQQTAKTLTLSHVSGVFSCYI
I
>HhamOBP1
MELYVCFTSQDFYIDLLQTIFQMNEQQMKAAQKLIRNVCQPKTKATDDQIVAMHIGNFNQ
DQNGMCYLSCVLNYYKLQLPDNSFDWETGVKVVETQAPPSYAPFVVEALKQCKDAVKNLD
DRCKAALEISQCVYEANPEVLNWR
>HhamOBP2
MIMKVDILLLLIFYFAVYTQALECGVSKLNTEQFKKVLTECVKDNETLSKIRDFTGLMSEE
EDAPLTTPAKEDESDQDQENEEQVPITRGRNISNIASKNVKLSKNRSKRASTRISSPRIT
INNKRLPGSTTPQTTTQENGTGANEDQEEKINNVDSNCVLQCIFDKLELADSNGLPDHKK
FTAALKTSTAGKQVNDFLQESMDQCFQEVDQSDNGCEYSTNLINCLGEKGKSNCEDWPAG
NLPF
>HhamOBP3
MKSLVLFVSFLVGINALDQSLIEEKKQQIIEWGLECAESEKATPEDIEALKNHQPPVSHQ
GRCLLFCVNKKLQLMNEDGSINVPHTTWLDKVKADDSELFEKLSKVYHSCIDKVTPKSDG
CDTALDLVSCLKEEGEKDGLNKIFHPDRK
>HhamOBP4
MNSILLLSFGLLVGVSALNDDLLAEMKQKVAQIGLECAESENAPEDDMIALLNKRPPKTH
EGKCILFCAAKKLGIMAADGSFGKGDDEWVAKAKSDDPDFVNKLIAAFEICKPEADKESD
NCEKAYVLSLCNYKEYLKSGIFKYF
>HhamOBP5
MMIFQAFVVVLLMALINCKDQRERVVDYHRDCLDEHGVHQDSLVEALEGNIMENEDFYKH
IFCTAKKAKVIDDDGVVNTDNVEDELRGHVDEHNIANVASIIRKCLVQKDDIMTTIKEAT
HCFYNEEHKLK
>HhamOBP6

MLSFGAIILLCGIVGGSAKVTLPPELQEYVDDLHALCLKRGSLTEDDHQSYDIHDKDPKM
MCYMKCLMLESKWMKPDGTIDYHFIESSAHPEVKEILVSALGKCRQIENGADLCEKSYNF
NFCMFEADPMNWFFV
>HhamOBP7
MAFFTFFFIFALVSVSLANPVQTRWNTVHQNCQADKKTYVPDEIFEQLKRGEKPTLPANF
GLHANCMLVQLDLQDEHGNVRLDGLRVAAGRQHTDSNHIDRIVNECAVNKESKETTAIGL
FKCLSKNHVDIGQFLKKND
>HhamOBP8
MFVFKIFFFIFILLTFSCNVLTADPDSNNETPLPAMFSEITSEYLKQCHEETGVSFEEVR
QKHESHKEPSEQDLCFKKCLMTKSGILDENGKTNWDKIKEKIPDNLKNDMQTCLQKAEPI
IECKDIENMKKCYRH
>HhamOBP9
MKLILVIIFAASLTKLQQTEAAIDNPRLLQWFKNCQLESGASNEDYETVKLRKVPTTPEG
ICMVQCLFTKLHIIDNGRFNERGFVITFSPVARGDLRKLGILKEIASECQKEIVDVDVDT
CNITEKVLHCFARNKNKLDLSRRN
>HhamOBP10
MIVIQLFYVLLLLLVPKTLGISEEMQELAKQLHATCVSETGAKEDDISNAVKGIFSEDEG
FKCYLKCLMSQMAIIDDDGTIDVEAMVAILPDELVEHATPIVRKCGSIKGSNACDSAWLT
HQCYYREGPEHYFLF
>HhamOBP11
MIKTIVIFCVFFTLILCETPDYITEHRKCQNIPELHLDHSVFENLHKNHSASKPTNFNKY
MMCVAKGINVLDDAGNINEAGVKAQVELVEKDAQKAEEITKTCSGLQKPTVDETINQLWR
CMFEKNIFRELKSDHSDESSSSESHEHKQPTI
>HhamOBP12
MWQPKMLFFLTVVYCINIASTRMTEKQFEAAVKLVRNMCIGKTKVNPGEIDKMHNGNWDV
DNNAQCYMWCSFNSYKLMRKDNHLDKKSVETQLALLPENLHDYVVNCVEKCENAPQNYED
KCVAAYEYAKCMYFYDPEVS
>HhamOBP13
MKFFIAILLSLAFAMVMGNASDEAKEKIKAANQKCQGDPKTAVDKTTLKAYIDSKGTAPA
PENIGAHALCVSKELNWQNADGSVNREHLKERISAHVDDASKVDEILNECAVVKDNEEAT
ARHLFRCFYKYAKDHGH
>HhamOBP14
MMNTILVPVWVLAFFATIIKAELTIEEKVKAIAFSRACLADTGVNPDLIKAARQGNFSND
SKFKDYVFCMSKRIGFQNDAGELQNDAIVKKVGAALGDMEAAKKLAASCVVVKENKQDTA
VASFKCYYDNTPNHLSIL
>HhamOBP15
MPSYLLKLSFIFAVISMISCQDFTEEQRKKIIKNRQECIEETKVNPDLIEKADLGDFAED
PALKCFTKCFYQKAGFVNEAGEVQRDVVEAKLPPQADKKKALEIVDKCAVKGKDACETVY
LIHKCYFEHTHPEPEKGAQESAPAKKDAKKA
>HhamOBP16
MYKLLLFFSFLFSTNQAHSIQDGIEKAQKECDALPPLPLDKLQQHAKHEDNVNIAVNFGA
HAYCMFQKLGLQNIEGHIQSGTLKEVVQRHISKPEQVDVVVKKCSLNGGTKEETAVKLLM
CLKDHHVGV
>HhamOBP17
MKYIVCVLVCALVAGALAKPEISASRLEESRNRMRNAHKTCQGNSATAVDEAALKTMLQN
PKAPRPENIGPHALCVSKALGWQNEDGSVNRDEVESRAKALYGDNERLQQLVNECTAPQS
SPEEAALKLIGCYRANAPRPTNAS
>HhamOBP18
MNIAMVKIVFLLGVCVFAVSGALLKNDNTTESINVNRCEIPTAAPKKIEEVINTCQDEIK
IAILSEALEALNVNEHKVSREKRSAFSEDERKIAGCLLQCVYRKMRAVNEYGFPTVDGMV
SLYTEGVTQKEYVLATLQAVTKCLVKAQKAYSLPTGVFQASKACDVAYDVFDCVSEEVAK
YCGQTP
>HhamOBP19
MVTKPLVLLFILVAAAVKSEMTAEQRERIFKMINSCMEETGTTDEMMRHAIKGDFPDDQR
FKDQMVCVGKKAGFIDDDGKVLKENLREHLKQNFGDDGKVDHILNNCFHEQDNTEDTVME
LAKCFYNEFYV
>HhamOBP20

MPYKFVVIALAITVTSIRCFTGEDLTNDLKFIKVCNLSSPIGAYSMNDVLENKNLDNTKS
RPFKCFLHCLLTKYGWMDQDGGFLLHDIRETLEQSDIQLGTMEYILYSCTATKSIDRCVR
AHFFTDCFWKKMEE
>HhamOBP21
MYNFYKFLLPLQVNALSKEDAEILRNVHESCIKETSVDPEIASKAIKGEYVDDEKLKEHL
LCSNKKLGFIDDDGNIIEDVTVTKLVKHFPNEQLVRSAVQKCAIKQDTPVETAFQFVKCM
RSLAPKDMNLDSFF
>HhamOBP22
MKLLIVLLSIIAITLADHHQHAGDDPHGLHAAHVKCHGDPNHETHSIELEDDHNEGDSTK
SHPPGHLGHLQCVEKELKLLNDDGKVNVEGVRTHVGHVITDATKIQEVVNECGVDQANSD
ATVLHIWRCLRKNQVFGVISKGHESREEEGHQHHHDHDHSH
>HhamOBP23
MKLLLPFFLIFLVTLAQEDPLNFETVHAKCQSDPETGIYGSAVDDFENDHSKTTLPENFN
KHVQCMANGVNIIKDNGKVNVEGIREQAKKVIHDTEEVEKVVKECAVDFATNHETLHQVW
QCLHKKGVLQSKNKHE
>HhamOBP24
MKFLLVGVFLFVALVQVKALSDKQKELLTQHYQQCIEVSKVDPTVLQKARTGDFANDDKL
KQHIWCIAKKSGFQNDAGVLQRSVIKTKLKEALKGDEVKTQELVNACAVANADPKIQAYN
AFKCIYQKAKINLL
>HhamOBP25
MTGRKQIISFTIIVNLLVVFNVCSFPKNCLLFFQFMFQTYVPNVSDKIRDFCIDNTGVTH
EIVEALLANPDKEMINEESCYVHCVETGFLAENGEINIKQFEHLKGNKFSDIDLNCLKSI
QVLEHCNETMLLRTCNA
>HhamOBP26a
MMKSLIFFCFVNVFLFQFVRSRPYHFETSVKKIIQECLDEVKMDRSIFNDVAVSNDAIII
PQDDIYKKFLVCSYKKQGFLSSDGKRMVYENLFEFLGYYYNKFLLRQLEVCKRIESDDPG
ELCFKNLECILKVLSGLKSLSTNMGENEIAR
>HhamOBP26b
MMKSLIFFCFVNVFLFQFVRVKCLDEVKMDRSIFNDVAVSNDAIIIPQDDIYKKFLVCSY
KKQGFLSSDGKRMVYENLFEFLGYYYNKFLLRQLEVCKRIESDDPGELCFKNLECILKVL
SGLKSLSTNMGENEIAR
>HhamOBP27
MKFLVVGVFLFVALVQVKALTDKQKELLAQHYKQCVELSKVDPTVLQKARSGDFANDDKL
KQHILCIAKKSGFQNEAGVLQRSVIKTKLKEALKGDEVKTQELVNTCAVAKPDPKLQAFE
AFKCIYQKAKINLL
>HhamOBP28
MASVTLLLVFLVKLLIFQPAEGTCDRSSILLGAEEVCWSATVKISDRIQYTTKSVLNFAK
EKLGLGSEEPFHPQQCDYYECIFKEMKMLNENGYPNYDKMIEWIDNNVIYNHAKIQYDKI
RDCNAALTASIVSDKYFSGDLIPHNEDERLQTKCDVAMEFMKCIASNGTECVIFTYP

Supplementary file 2: In silico transcript abundance for *Hypothenemus hampei* chemosensory genes

A coffee berry borer (Hypothenemus hampei) genome assembly reveals a reduced chemosensory receptor gene repertoire and male-specific genome sequences

Lucio Navarro, Erick M. Hernandez-Hernandez, Jonathan Nuñez, Flor E. Acevedo, Alejandro Berrio, Luis M. Constantino, Beatriz E. Padilla-Hurtado, Diana Molina, Carmenza Gongora, Ricardo Acuña, Jeff Stuart, Pablo Benavides

Transcript abundance calculated using Kallisto. Abundance are expressed as Transcript Per Million (TPM). Sleuth pipeline was used to estimate differences at the TPM abundances between female and male (see Methods in articule main text).

Odorant Receptors

OR name	larv1	larv2	larv3	fem1	fem2	fem3	male1	male2	male3	FDR-adjusted p-value (female-vs-male)	Larva mean	Female mean	Male mean
HhamOrCo	0.331939	0.196716	0.308879	2.96884	3.39498	2.40113	0.676527	0.502577	0.540144	0.000537098	0.279178	2.92165	0.57308267
HhamOR1	0	0	0.0568553	0.075037	0	0	0.0218087	0.0392837	0.0435171	NA	0.018951767	0.02501233	0.03486983
HhamOR2	0.601357	0.455178	0.255287	0.336862	0.545616	1.01906	1.29216	0.934455	0.664219	0.055643568	0.437274	0.633846	0.96361133
HhamOR3	0.00933164	0.054468	0.0285152	0	0.0304729	0.0258771	0.360955	0.46301	0.458339	0.001548075	0.030771613	0.01878333	0.42743467
HhamOR4	0.764557	1.08212	0.584173	4.48519	4.88065	4.91482	3.25861	1.68743	2.64177	0.020083522	0.810283333	4.76022	2.52927
HhamOR5	0.0941884	0.183251	0.230248	0.379857	0.123028	0.26119	1.01571	0.59661	0.969299	0.021878006	0.169229133	0.25469167	0.86053967
HhamOR6	0.779631	0.369946	0.581035	3.90038	3.62626	3.26455	4.36596	4.1342	4.42693	0.025685813	0.576870667	3.59706333	4.30903
HhamOR7	0.0772764	0	0	0.311646	0.315413	0.214293	0.679365	0.673074	0.81332	0.018658966	0.0257588	0.28045067	0.72191967
HhamOR8	1.13496	1.30322	1.30767	1.95096	2.43035	2.0122	1.22129	0.64818	0.60924	0.013942404	1.248616667	2.13117	0.82623667
HhamOR9	0.866478	1.36309	1.35208	0.148702	0.481634	0.255615	0.691491	0.817399	0.819267	0.024299087	1.193882667	0.295317	0.77605233
HhamOR10	0.0375584	0	0	0.0757355	0.0613235	0.0520757	0.0660362	0.118951	0	NA	0.012519467	0.0630449	0.0616624
HhamOR11	0.291418	0.212641	0.166979	0.293824	0.297403	0.404056	8.66751	11.69	11.7576	7.09E−05	0.223679333	0.331761	10.7050367
HhamOR12	0	0	0.0573836	1.13603	1.16515	1.04151	0.264145	0.218076	0.131767	0.006426538	0.019127867	1.11423	0.20466267
HhamOR13	0.0953768	0.0742217	0.116573	0.153858	0.373731	0.317383	0.335393	0.24166	0.356917	0.03241474	0.0953905	0.28165733	0.31132333
HhamOR14	0.0926131	0.036176	0	0.220368	0.178442	0.202028	1.44927	1.31211	1.24992	0.003638527	0.0429297	0.20027933	1.3371
HhamOR15	0.0377931	0.0367642	0	0.152417	0.123412	0.157204	0.132898	0.0997463	0.13259	NA	0.024852433	0.14434433	0.12174477
HhamOR16	1.9502	2.53702	1.87165	0	0.064522	0	3.45803	2.58505	1.56004	0.000278589	2.119623333	0.02150733	2.53437333
HhamOR17	0	0	0	0.0840857	0.204218	0.115646	0	0	0	NA	0	0.1346499	0
HhamOR18	0	0	0.117684	0	0.0628821	0	0.0677199	0.0813244	0	NA	0.039228	0.0209607	0.04968143
HhamOR19	0.0187211	0	0	0	0	0	0.0658316	0	0.131359	NA	0.006240367	0	0.0657302
HhamOR20	0.112675	0.0730734	0	0.302942	0.735882	0.416605	0.198109	0.218076	0.395301	0.03305299	0.061916133	0.485143	0.27049533
HhamOR21	0	0	0	0.572149	0.521228	0.639259	0.685895	0.505406	0.622147	0.032712086	0	0.57754533	0.60448267
HhamOR22	0.038762	0.0377033	0	0	0	0.053745	0.159027	0.143232	0.0906583	NA	0.025488433	0.017915	0.13097243
HhamOR23	0	0	0.0684012	0	0	0	0.0787659	0.023651	0.10475	NA	0.0228004	0	0.06905563
HhamOR24	0	0	0	0.101165	0.163732	0.139163	0.176962	0.0265342	0.117702	NA	0	0.13468667	0.10706607
HhamOR25	0.0375584	0.0730734	0	0.0757355	0.0613235	0.0520757	0.330181	0.237902	0.0439223	NA	0.036877267	0.0630449	0.20400177
HhamOR26	0.866591	1.01153	0.706101	0.776637	0.754585	0.587429	1.64785	0.36596	0.810731	0.159128371	0.861407333	0.706217	0.94151367
HhamOR27	0.092744	0.07218	0	0.299228	0.0605728	0	2.26114	1.70288	1.64858	0.00852072	0.054974667	0.1199336	1.87086667
HhamOR28	0	0	0	0	0	0	0	0.316595	0.174204	NA	0	0	0.16359967
HhamOR29	1.12422	1.55813	1.05307	3.5724	2.78176	3.4481	2.39397	1.99571	1.59367	0.031800802	1.24514	3.26742	1.99445
HhamOR30	0	0	0.0600497	0.244362	0.128347	0.0949205	0	0	0	NA	0.020016567	0.1558765	0
HhamOR31	2.26973	3.46149	2.53677	2.80093	2.43426	2.25454	2.30289	1.2515	1.54513	0.041719207	2.755996667	2.49657667	1.69984
HhamOR32	0	0	0	0.631468	0.566117	0.323852	0	0	0.113276	0.009588886	0	0.50714567	0.03775867
HhamOR33	0	0	0	0	0	0	0	0.0281403	0	NA	0	0	0.0093801
HhamOR34	0	0	0	0	0.129891	0.0744486	0.0949026	0.0864991	0.0547622	NA	0	0.0681132	0.0787213
HhamOR35	0.0228179	0	0	0	0	0	0.0267511	0	0.0533626	NA	0.007605967	0	0.02670457
HhamOR36	0	0.0700394	0.0549986	0.290329	0.352645	0.349339	0.105471	0.0569933	0	0.016311023	0.041679333	0.330771	0.05415477
HhamOR37	0.0933164	0.254184	0.114061	0	0	0.103508	2.66888	3.80259	3.49211	0.000540225	0.1538538	0.03450267	3.32119333
HhamOR38	0.0566897	0.0367642	0	0.228626	0.123412	0.0524012	0.088599	0.0199493	0.13259	NA	0.0311513	0.13481307	0.08037943
HhamOR39	0.017733	0	0	0.214556	0.115828	0.196695	0.0415694	0.0187187	0.0414765	NA	0.005911	0.175693	0.03392153
HhamOR40	0	0	0	0.282422	0.380966	0.388469	0.109486	0.123285	0.163793	0.019936262	0	0.350619	0.132188
HhamOR41	0.0629877	0.285826	0.0641385	0.423346	0.479808	0.291122	0.492259	0.2217	0.491039	0.034187951	0.137650733	0.398092	0.401666
HhamOR42	0.0333167	0.0648468	0	0.201561	0.163235	0.230966	0.214766	0.246156	0.311713	0.028623198	0.032721167	0.19858733	0.257545
HhamOR43	0	0	0	0	0	0	0.0266505	0	0	NA	0	0	0.0088835
HhamOR44	0.0223954	0.0435426	0.136802	0.812779	1.02341	0.55896	0.682638	0.756832	0.419001	0.039756672	0.06758	0.798383	0.61949033
HhamOR45	0	0	0	0	0	0.116855	0.0246988	0	0	NA	0	0.03895167	0.00823293
HhamOR46	0.26303	0	0	0.957438	0.892093	0.54635	0.304897	0.251521	0.700254	0.050013893	0.087676667	0.798627	0.41889067

HhamOR47	0	0	0.0556595	0	0.0594806	0	0.128091	0.038454	0	NA	0.018553167	0.01982687	0.055515
HhamOR48	0.0941884	0.0366501	0	0.151943	0.430598	0.138446	1.25705	1.36984	1.18179	0.007176052	0.043612833	0.240329	1.26956
HhamOR49	0.678336	0.45244	0.236872	0.390806	0	0.053745	18.8788	18.5178	19.1742	0.001280463	0.455882667	0.14818367	18.8569333
HhamOR50	0.269744	0.0437037	0.411928	0.0906442	0.146744	0.0623376	0.948708	1.40248	1.41938	0.003375183	0.2417919	0.0999086	1.256856
HhamOR51	0.0695027	0	0.0707523	0	0	0	0.733361	1.19698	0.812696	0.000164603	0.046751667	0	0.91434567
HhamOR52	0	0	0	0	0	0	0	0	0	NA	0	0	0
HhamOR53	0.102143	0	0	0.0890511	0	0.0596324	0.0770833	0.0862789	0	NA	0.034047667	0.04956117	0.05445407
HhamOR54	0	0	0	0.0935394	0.0757075	0	0.0815888	0.048998	0.054249	NA	0	0.05641563	0.06161193
HhamOR55	0	0.0421439	0	0	0	0	0	0	0	NA	0.014047967	0	0
HhamOR56	0	0	0	0	0.0653745	0.0555249	0.070413	0.0641892	0.187316	NA	0	0.0402998	0.10730607
HhamOR57	0.729458	1.42536	0.87925	2.27707	2.59252	1.98782	2.98231	1.64991	1.67953	0.050241463	1.011356	2.28580333	2.10391667
HhamOR58	0	0	0	0	0	0.0745303	0.0674688	0.0567821	0	NA	0	0.02484343	0.04141697
HhamOR59	0.0575826	0.0559116	0	0.348238	0.37561	0.239553	1.1142	0.730179	0.336625	0.037714825	0.0378314	0.32113367	0.72700133
HhamOR60	0	0.0365367	0	0	0	0.104151	0	0	0.0439223	NA	0.0121789	0.034717	0.01464077
HhamOR61	0	0.0963171	0.0756631	0.199845	0.32345	0.0687257	0.406782	0.209409	0.289757	0.034308175	0.057326733	0.19734023	0.30198267
HhamOR62	0	0	0	0	0	0.0794724	0.0336036	0	0	NA	0	0.0264908	0.0112012
HhamOR63	0.0244469	0	0.0746487	0.295731	0.558433	0.203398	0.229311	0.20658	0.0571711	0.034769326	0.033031867	0.35252067	0.16435403
HhamOR64	0	0	0.0986287	0	0	0	0.0394573	0.052138	0	NA	0.032876233	0	0.03053177
HhamOR65	0.228574	0.484957	0.190437	0.502782	0.678392	0.345744	5.1641	7.43801	6.41494	0.000230028	0.301322667	0.50897267	6.33901667
HhamOR66	0.0520466	0.0949146	0	0.602793	0.908135	0.284706	0.445727	0.48407	0.471514	0.052361059	0.048987067	0.59854467	0.46710367

Gustatory Receptors

GR name	larv1	larv2	larv3	fem1	fem2	fem3	male1	male2	male3	FDR-adjusted p-value (female-vs-male)	Larva mean	Female mean	Male mean
HhamGR1	0.116931	0.0650254	0.255287	0.336862	0.163685	0.509529	3.70027	3.36756	4.4151	0.000689496	0.1457478	0.336692	3.82764333
HhamGR2	0.162554	0.316415	0.198752	0.590063	0.849565	0.631057	0.552493	0.54901	0.228132	0.043760749	0.225907	0.69022833	0.44321167
HhamGR3	0.0194433	0	0.178224	0.313649	0.190462	0.107836	0.250704	0.0821103	0.0454748	NA	0.0658891	0.20398233	0.12609637
HhamGR4	0.20155	0.0489572	0.0769197	1.32062	1.06868	0.349357	6.02628	9.31467	10.8406	0.002171555	0.1091423	0.91288567	8.72718333
HhamGR5	0	0	0.111029	0.146789	0.35602	0	0.384511	0	0.0851487	NA	0.037009667	0.167603	0.15655323
HhamGR6	0	0.0356541	0	0	0.0598403	0	0.021478	0.0193437	0	NA	0.0118847	0.01994677	0.01360723
HhamGR7	0.287951	0.593398	0.517709	1.16136	1.21713	1.17424	2.95803	3.00359	2.37712	0.004579313	0.466352667	1.18424333	2.77958
HhamGR8	0	0	0	0.160962	0.130318	0.276708	0.374296	0.0632116	0.0466747	NA	0	0.18932933	0.1613941
HhamGR9	0.281688	0.43844	0.344302	0.378678	1.47176	1.09359	0.264145	0.594754	0.790601	0.152788953	0.35481	0.98134267	0.54983333
HhamGR10	0.181411	0.353198	0.184869	0.121952	0.148164	0.293439	0.30119	0.223356	0.353618	0.033231341	0.239826	0.18785167	0.29272133
HhamGR11	0	0	0	0.131937	0.214024	0.23139	0.203805	0.0689116	0.0765038	NA	0	0.19245033	0.1164068
HhamGR12	0.040857	0	0	0	0.0666962	0.0566506	0.0239472	0.0647092	0.0477777	NA	0.013619	0.0411156	0.04547803
HhamGR13	0.0218295	0	0	0.0880286	0.213776	0.121074	0	0.0461043	0.102105	NA	0.0072765	0.14095953	0.0494031
HhamGR14	0	0	0	0	0	0.0505069	0.0853942	0	0.1278	NA	0	0.01683563	0.07106473
HhamGR15	0.0188377	0	0	0.227914	0.246056	0	0.132484	0.039774	0.044059	NA	0.006279233	0.15799	0.07210567
HhamGR16	0.203597	0.198	0.186601	0.492633	0.59825	0.508138	1.38425	1.26832	1.38089	0.0130726	0.196066	0.533007	1.34448667
HhamGR17	0.350279	0.143483	0.16901	0.594809	0.541839	0.408984	2.80918	2.7052	2.75964	0.000799348	0.220924	0.51521067	2.75800667
HhamGR18	0.0372117	0	0.170566	0.450222	0.48607	0.20638	0.915966	0.373195	0.435171	0.049957809	0.069259233	0.38089067	0.57477733
HhamGR19	0.481429	0.405857	0.477326	1.15627	2.78943	0.80681	7.44335	7.1354	8.0159	0.002192129	0.454870667	1.58417	7.53155
HhamGR20a	0.279483	0.288723	0.0863329	1.03338	1.15921	0.740102	0.802666	0.86791	0.410279	0.050227496	0.218179633	0.977564	0.69361833
HhamGR20b	0.200857	0.284545	0.358705	0.11931	0.243239	0.246568	0.254315	0.124305	0.284768	0.040298138	0.281369	0.203039	0.22112933
HhamGR21a	0	0	0	0.0823826	0	0	0.0247005	0	0	NA	0	0.02746087	0.0082335
HhamGR21b	0.0223503	0	0	0.421882	0	0.0647463	0.0759558	0	0.0511894	NA	0.0074501	0.16220943	0.04238173
HhamGR22a	0.0226948	1.78E-09	0	0	0.08626	0	0.0613863	0	0.0529483	NA	0.007564934	0.02875333	0.03811153
HhamGR22b	0	0.0449343	0	0	0.0836245	0.221122	0.122581	0.0479068	0	NA	0.0149781	0.10158217	0.05682927
HhamGR23a	0	0.197338	0	0	0.1922	0.110816	0.0843796	0	0.22474	NA	0.065779333	0.10100533	0.10303987
HhamGR23b	0.104616	0	0.0639172	0	0.27323	0.117825	0.13374	0.0883726	0.218474	NA	0.056177733	0.13035167	0.1468622
HhamGR24	0.105102	0.124714	0.0624099	0.0823826	0.0666962	0.0630422	0.073613	0.0676154	0	NA	0.097408633	0.070707	0.04707613
HhamGR25	0.575318	0.728944	0.55793	1.06379	1.29425	1.32737	3.62205	5.90656	5.44936	0.000732302	0.620730667	1.22847	4.99265667
HhamGR26	0.231829	0.0751666	0.236117	0.857027	1.38782	0.910746	2.17397	1.91724	1.62664	0.019571443	0.181037533	1.05186433	1.90595
HhamGR27	0.212169	0.123781	0.0648117	0.171119	0.415582	0.117675	0.0746167	0.224038	0.0992409	NA	0.133587233	0.234792	0.13263187
HhamGR28	0.495959	0.408219	0.64115	0.923151	0.249154	0.264486	0.357752	0.24166	0.178459	0.068850163	0.515109333	0.47893033	0.25929033
HhamGR29	0.0767858	0	0	0.0774173	0	0.0532331	0.318563	0.34453	0.256755	0.012579009	0.025595267	0.04355013	0.306616
HhamGR30	0.0863828	0.251964	0.0659656	0	0.0704972	0.179665	0.430385	0.798164	0.808094	0.013030433	0.1347708	0.0833874	0.678881

HhamGR31	0.388334	0.32382	0.904135	2.01362	1.44931	1.38454	1.14879	0.39043	0.692018	0.041103881	0.538763	1.61582333	0.743746
HhamGR32	1.41692	0.931652	0.975553	2.01202	2.67153	2.21366	1.63754	1.60136	1.63361	0.026680599	1.108041667	2.29907	1.62417
HhamGR33	0.0219086	0	0.133837	0	0.214548	0.425294	0.077052	0.208222	0.0512373	NA	0.0519152	0.21328067	0.11217043
HhamGR34	0.0487661	0.0316415	0	0.0655626	0	0	0.457236	0.463227	0.494285	0.002703233	0.026802533	0.0218542	0.47158267
HhamGR35	0.0433951	0.0381911	0	0	0.192312	0	0.322188	0.331646	0.229587	0.026796531	0.0271954	0.064104	0.29447367
HhamGR36	0.547716	0.639001	1.33837	2.73876	2.50306	2.06571	4.4947	2.54494	2.10073	0.0893633	0.841695667	2.43584333	3.04679
HhamGR37	0.0390121	0	0	0.078665	0	0.0540918	0.731674	0.864944	0.547457	0.003696396	0.013004033	0.04425227	0.71469167
HhamGR38	0	0.0897299	0	0	0	0	0.0541155	0.024374	0	NA	0.029909967	0	0.02616317
HhamGR39	0.0212916	0.041405	0.130078	0	0	0	0.454886	0.224829	0.448156	0.000655581	0.0642582	0	0.375957
HhamGR40	0	0	0	0	0	0	0.526003	0.467578	0.694686	0.000272618	0	0	0.56275567
HhamGR41	0.305396	0.1584	0.248801	0.410527	0.531778	0.338759	0.429596	0.580418	0.523786	0.028107453	0.237532333	0.42702133	0.51126667
HhamGR42	0.0442987	0	0	0.178634	0	0.0614241	0	0.0467812	0	NA	0.014766233	0.08001937	0.01559373
HhamGR43	0	0	0.0619922	0	0	0	0.0475726	0.0214247	0.0474573	NA	0.020664067	0	0.0388182
HhamGR44	0	0.16001	0	0	0	0.114072	0.0241102	0.0434335	0	NA	0.053336667	0.038024	0.02251457
HhamGR45	0	0	0	0	0	0	0.203809	0.26515	0.225922	0.000912485	0	0	0.231627
HhamGR46	0	0	0.0575619	0	0	0	0.0669725	0.019887	0.100514	NA	0.0191873	0	0.06245783
HhamGR47	0	0	0	0	0	0	0	0	0	NA	0	0	0
HhamGR48	0.0415589	0	0	0	0	0.0576241	0.0487179	0	0	NA	0.013852967	0.01920803	0.0162393
HhamGR49	0	0	0	0	0.071003	0	0	0.0229691	0.050869	NA	0	0.02366767	0.0246127
HhamGR50	0.126414	0.0819481	0.0643613	0.339856	0.137564	0.0584273	0.172891	0.0667422	0.147825	NA	0.0909078	0.17861577	0.12915273
HhamGR51	0.0845708	0.205583	0.0645857	0.259496	0.278575	0.0591904	0.0998104	0.178603	0.0988942	NA	0.1182465	0.19908713	0.1257692
HhamGR52	0.0229043	0	0	0	0	0	0	0	0	NA	0.007634767	0	0
HhamGR53	0.0209959	0.0816647	0	0	0.137088	0	0.0492259	0.04434	0.0491039	NA	0.0342202	0.045696	0.0475566
HhamGR54	0.0381507	0	0	0	0	0	0.658191	0.60676	0.624605	0.000178269	0.0127169	0	0.629852
HhamGR55	0.230156	0.4883	0	0.168753	0	0.116046	1.00564	2.01048	2.25097	0.006205781	0.239485333	0.094933	1.75569667
HhamGR56	0	0	0.0628332	0	0	0	0.0241102	0	0	NA	0.0209444	0	0.00803673
HhamGR57	0.0290681	0.0564461	0.0887034	0.937554	1.20842	0.403094	1.2597	0.640801	0.434129	0.084237819	0.058072533	0.84968933	0.77821
HhamGR58	0.0310054	0	0	0.124997	0.131303	0	0	0.102842	0	NA	0.010335133	0.08543333	0.03428067
HhamGR59	0	0	0	0	0.0632842	0.10749	0.0681544	0.0409233	0	NA	0	0.05692473	0.03635923
HhamGR60	0.106084	0.0412604	0.129623	0.171119	0.0692637	0.0588374	1.59182	4.90644	4.31698	0.001015797	0.092322467	0.09974003	3.60508
HhamGR61	3.08865	4.18719	2.19248	5.69747	7.45275	6.15198	3.59468	1.90643	1.67868	0.007338934	3.156106667	6.43406667	2.39326333
HhamGR62	0.124496	0.103827	0	0.502102	0.522757	0.493184	0.208459	0.300382	0.49918	0.030976745	0.076107667	0.50601433	0.336007

Ionotropic Receptors

IR name	larv1	larv2	larv3	fem1	fem2	fem3	male1	male2	male3	FDR-adjusted p-value (female-vs-male)	Larva mean	Female mean	Male mean
HhamIR8a	0.0388147	0.0302702	0.0712668	0.657697	0.837641	0.667239	0.973122	0.638556	0.853612	0.031745054	0.0467839	0.720859	0.82176333
HhamIR21a	0.0793081	0.0579603	0.0909842	0.15997	0.226847	0.274874	0.255536	0.303245	0.255113	0.029802843	0.0760842	0.22056367	0.271298
HhamIR25a	3.66189	4.1347	3.86376	1.88544	2.05281	2.20465	3.44009	5.35456	5.61372	0.002128689	3.886783333	2.04763333	4.80279
HhamIR40a	0.0326176	0	0.0501328	0.0327527	0	0.0223274	0.339991	0.300643	0.390482	0.002862858	0.027583467	0.01836003	0.34370533
HhamIR41a1	0	0	0	0.23546	0.267077	0.453177	0.068396	0.123159	0.0819285	0.019813359	0	0.31857133	0.09116117
HhamIR41a2	0.0237144	0	0.036265	0.143491	0.077503	0.197265	0.18062	0.050037	0.166426	0.047637693	0.019993133	0.13941967	0.132361
HhamIR60a	0	0.019773	0	0.122794	0.033167	0.112533	0.285317	0.117728	0.0949466	NA	0.006591	0.089498	0.1659972
HhamIR68a	0.0659358	0.0734356	0.0288184	0.303998	0.246347	0.28729	0.640094	0.60604	0.661093	0.020140074	0.056063267	0.27921167	0.63574233
HhamIR75a	0.0317717	0	0	0.0854456	0.207703	0.11746	0.210952	0.268119	0.22298	0.032390331	0.010590567	0.13686953	0.234017
HhamIR75b	0.120407	0.0303519	0.0409099	0.0539647	0.0874315	0	2.73092	3.26811	3.4416	0.000584224	0.0638896	0.04713207	3.14687667
HhamIR75c	1.24131	1.2768	1.51744	1.07278	1.2162	1.03265	1.20551	1.04825	1.16134	0.03159361	1.345183333	1.10721	1.13836667
HhamIR75d	0.0737457	0.0718279	0.150358	0.396636	0.200835	0.102242	0.316854	0.129674	0.115008	0.067186744	0.098643867	0.23323767	0.18717867
HhamIR75e	0.0910119	0.019707	0.123743	0.856687	0.628067	0.30843	0.947876	0.78934	1.44309	0.067480139	0.078153967	0.597728	1.060102
HhamIR75f	0.0487878	0.147089	0	0.110923	0.240112	0.137548	1.85368	2.55291	2.1172	0.002472329	0.065292267	0.162861	2.17459667
HhamIR75g	0.0598729	0.163294	0.109871	0.338126	0.234808	0.199219	0.224504	0.290565	0.25211	0.032867595	0.111012633	0.25738433	0.25572633
HhamIR75h	0.0701365	0.0607741	0.071542	0.880319	0.535103	0.95071	3.38715	2.6956	2.4978	0.001820267	0.0674842	0.78871067	2.86018333
HhamIR75i	0.324358	0.0675957	0.113298	1.38262	2.3952	0.935356	1.36147	1.86259	1.95663	0.07890864	0.168417233	1.57105867	1.72689667
HhamIR76b	0.319019	0.353705	0.772627	2.46853	2.48888	2.0912	1.91025	1.179	1.67306	0.031808605	0.481783667	2.34953667	1.58743667
HhamIR93a	0.530604	0.608524	0.334293	2.48696	3.16373	2.81254	1.55406	2.11483	1.84382	0.021873977	0.491140333	2.82107667	1.83757
HhamIR100a	0.0100787	0	0.0308328	0	0.0988384	0.139728	0.0472358	0.0850494	0.0943138	NA	0.013637167	0.07952213	0.075533
HhamIR100b	0	0	0.0323399	0	0	0	0.0247745	0.0446083	0.0247322	NA	0.010779967	0	0.03137167
HhamIR101	0.0117879	0.0688944	0	0	0	0.0653706	0.42819	0.932702	0.909994	0.002192129	0.0268941	0.0217902	0.756962

HhamIR102	0.0114747	0	0	0	0	0.0318169	0.121009	0.111018	0.0810992	NA	0.0038249	0.01060563	0.1043754
HhamIR103	0.0856884	0.143076	0.0748753	0.0987576	0.360042	0.203655	0.616797	1.03319	0.744529	0.017635023	0.101213233	0.2208182	0.798172
HhamIR104	0	0	0	0.0523443	0	0	0.0304129	0.0136918	0	NA	0	0.0174481	0.01470157
HhamIR105	0.329364	0.102638	0.201436	0.212568	0.12915	0.146124	0.617539	0.430926	0.339002	0.025549899	0.211146	0.162614	0.462489
HhamIR106	0.07199	0.0467478	0.0733921	0.387196	0.352909	0.299421	0.267128	0.22785	0.336814	0.029477484	0.0640433	0.34650867	0.277264
HhamIR107	0	0	0	0	0.0826833	0.0350788	0.0148245	0	0.0295939	NA	0	0.03925403	0.01480613
HhamIR108	0	0	0	0	0	0	0.0449439	0.0269778	0	NA	0	0	0.0239739
HhamIR109	0	0	0	0	0.087625	0	0.0157125	0	0	NA	0	0.02920833	0.0052375
HhamIR110	0	0.0259985	0	0	0	0	0.0156431	0.0140852	0.0312263	NA	0.008666167	0	0.0203182
HhamIR111	0	0	0	0	0.118813	0	0.0142003	0	0	NA	0	0.03960433	0.00473343
HhamIR112	0.0378735	0	0	0.101849	0.082511	0.070011	0.340251	0.452872	0.413449	0.016291772	0.0126245	0.08479033	0.40219067

Odorant-binding proteins

OBP name	larv1	larv2	larv3	fem1	fem2	fem3	male1	male2	male3	FDR-adjusted p-value (female-vs-male)	Larva mean	Female mean	Male mean
HhamOBP1	7.20665	6.02799	6.40559	13.1866	14.6404	9.29062	8.03848	3.86152	5.27056	0.023699054	6.546743333	12.37254	5.72352
HhamOBP2	7.92892	7.05161	10.8824	66.0574	50.1864	39.6776	18.4841	13.027	10.5056	0.003308059	8.620976667	51.9738	14.0055667
HhamOBP3	92.973	81.3854	120.487	281.139	246.241	220.214	302.837	187.006	206.259	1	98.2818	249.198	232.034
HhamOBP4	9.71037	6.92706	8.94947	74.9959	66.272	61.6659	74.8649	41.2824	45.192	0.427695657	8.528966667	67.6446	53.7797667
HhamOBP5	0.690369	0.828802	0.261392	72.92	75.1729	49.3284	0.50711	1.09992	1.81309	0.000124264	0.593521	65.8071	1.14004
HhamOBP6	0.489831	1.25558	1.48457	142.258	128.302	108.955	27.4365	15.6883	13.7243	0.000480644	1.076660333	126.505	18.9497
HhamOBP7	5002.5	5050.57	4521.54	2128.33	1491.12	1668.08	3240.96	2014.09	1607.43	0.248151233	4858.203333	1762.51	2287.49333
HhamOBP8	54.2896	51.3219	94.27	196.795	143.381	151.087	170.758	81.9086	87.6829	0.245186678	66.62716667	163.754333	113.449833
HhamOBP9	0.65515	0.841115	1.32529	3.80947	2.36136	2.0197	1.96686	1.23569	2.04022	0.039214652	0.940518333	2.73017667	1.74759
HhamOBP10	29.3082	22.7574	29.6913	108.418	123.541	99.4411	54.2014	34.5836	37.1699	0.001233988	27.2523	110.4667	41.9849667
HhamOBP11	304.593	407.902	325.662	3.20825	1.9401	2.02639	70.8137	51.3979	34.1259	0.000147577	346.0523333	2.39158	52.1125
HhamOBP12	2.67668	2.64908	3.24722	50.4825	44.1386	47.9536	38.3683	27.5142	33.2198	0.032739448	2.85766	47.5249	33.0341
HhamOBP13	4156.18	4801.46	3109.5	1972.02	1490.01	1189.03	7406.64	4075	3144.87	0.009798352	4022.38	1550.35333	4875.50333
HhamOBP14	5.33535	3.32045	5.94754	17.684	11.1911	10.2316	80.0212	64.4586	87.392	0.000400942	4.86778	13.0355667	77.2906
HhamOBP15	2.14851	1.68263	2.03867	10.272	10.6791	11.3621	126.723	250.249	295.638	1.01E−04	1.956603333	10.7710667	224.203333
HhamOBP16	4.2639	3.06988	7.80051	25.7317	25.3065	21.9379	8.97923	8.39914	7.67204	0.001562455	5.044763333	24.3253667	8.35013667
HhamOBP17	883.506	1472.09	658.008	39.5599	45.8104	41.6058	845.495	1436.25	1283.3	1.39E−05	1004.534667	42.3253667	1188.34833
HhamOBP18	2.03098	7.01635	1.32473	4.28618	4.72009	4.42649	27.2435	41.6168	47.9201	8.39E−05	3.457353333	4.47758667	38.9268
HhamOBP19	14.0663	14.9184	16.2063	75.0034	64.2742	71.598	62.5773	41.1553	41.0966	0.081522855	15.06366667	70.2918667	48.2764
HhamOBP20	0	0	0	0	0	0	0	0	0	NA	0	0	0
HhamOBP21	27.6404	27.0405	36.8642	205.808	171.868	159.126	82.1754	35.5877	38.2561	0.006353898	30.51503333	178.934	52.0064
HhamOBP22	573.194	544.521	539.778	180.341	164.325	125.23	799.987	636.385	548.381	0.000314708	552.4976667	156.632	661.584333
HhamOBP23	58.3996	41.2005	47.6125	124.484	89.5408	84.2587	111.876	60.801	27.648	0.330272672	49.07086667	99.4278333	66.775
HhamOBP24	1110.75	1453.83	1160.85	1064.34	978.655	840.867	1906.18	594.182	680.688	1	1241.81	961.287333	1060.35
HhamOBP25	0.476954	0	0.24099	15.0365	8.24491	9.9251	3.82946	2.78462	3.34107	0.007023134	0.239314667	11.0688367	3.31838333
HhamOBP26a	42.2083	42.9717	53.3137	115.425	75.5218	97.6021	109.677	132.8	138.157	0.078176718	46.16456667	96.1829667	126.878
HhamOBP226b	0.822457	0	3.00945	0	2.18346	0	1.22943	0.395855	0.761511	NA	1.277302333	0.72782	0.79559867
HhamOBP27	7765.96	9508.24	6514.94	2510.33	2461.92	2062.21	3725.32	1248.82	1348.24	1	7929.713333	2344.82	2107.46
HhamOBP28	1.77158	2.01457	2.3778	14.4833	14.5726	15.6095	11.2542	6.45549	6.45371	0.022166028	2.05465	14.8884667	8.05446667
